# Neuronal Endothelin a Receptor Mediates Experimental and Clinical Vascular Pain through an Endothelial‐Neural Axis

**DOI:** 10.1002/advs.202512375

**Published:** 2025-08-14

**Authors:** Zuo‐Jie Jiang, Di Mu, Su Liu, Peng‐Bo Jing, Bin Wu, Xiao‐Mei Yang, Jia‐Yi Ge, Qing‐Yi Li, Hao‐Hao Chen, Feng‐Ming Zhang, Bing Wang, Ying‐Ying Zhang, Linnan Qian, Zi‐Yi Zhu, Yu‐Sen Ou, Shi‐Yu Sun, Lin Luo, Yu Feng, Changyu Jiang, Zhuo Sun, Yong Chen, Hai‐Li Pan, Bin Wang, Fen‐Fei Gao, Jie Wu, Zhigang Lu, Tong Liu, Yan‐Gang Sun, Xing‐Jun Liu

**Affiliations:** ^1^ School of Pharmacy Nantong University Nantong Jiangsu Province 226019 China; ^2^ Pain and Related Disease Research Laboratory Shantou University Medical College Shantou Guangdong Province 515041 China; ^3^ Department of Pharmacology Shantou University Medical College Shantou Guangdong Province 515041 China; ^4^ Guangdong Institute of Intelligence Science and Technology Zhuhai Guangdong Province 519031 China; ^5^ Department of Anesthesiology The Affiliated Hospital of Xuzhou Medical University Xuzhou Jiangsu Province 221000 China; ^6^ Institute of Pain Medicine and Special Environmental Medicine Nantong University Nantong Jiangsu Province 226019 China; ^7^ Xuzhou Medical University Xuzhou Jiangsu Province 221004 China; ^8^ School of Integrative Medicine Nanjing University of Chinese Medicine Nanjing Jiangsu Province 210023 China; ^9^ School of Design Jiangnan University Wuxi Jiangsu Province 214122 China; ^10^ Department of Rheumatology and Immunology The Fourth Affiliated Hospital of Soochow University Suzhou Jiangsu Province 215004 China; ^11^ Department of Pain Medicine Shenzhen Municipal Key Laboratory for Pain Medicine The Affiliated Nanshan Hospital of Shenzhen University Shenzhen Guangdong Province 518000 China; ^12^ Center for Excellence in Brain Science and Intelligence Technology Chinese Academy of Science Shanghai 200031 China

**Keywords:** endothelial cell, ET‐1, ETAR, optogenetic or chemogenetic activation, vascular pain

## Abstract

Vascular dysfunction causes vascular pain (VP), the most common clinical manifestation of prevalent vascular diseases, while the mechanisms remain elusive. Mouse models are developed by mimicking peripheral vascular diseases and combining multiple strategies to demonstrate that primary sensory neuronal endothelin A receptor (ETAR) mediates experimental and clinical VP through an endothelial‐neural axis. Endothelial cells (ECs), but not macrophages or smooth muscle cells (SMCs), release endothelin‐1 (ET‐1) and directly activate primary sensory neurons by binding to ETAR on sensory neurons, resulting in VP. Mice that underwent vessel ligation exhibit long‐lasting mechanical hyperalgesia, but no heat hyperalgesia or cold allodynia, without inflammatory cell infiltration into or gliosis in the nervous system. These mice also display a moderate decrease in blood perfusion in the affected hindpaws, without evident ischemic injury or tissue necrosis after vessel ligation. Activating ECs with optogenetics or chemogenetics elicits spontaneous pain‐like behaviors and lasting mechanical hyperalgesia. Blocking the increased endothelial ET‐1/neural ETAR signal reduces pain‐like behaviors caused by vessel ligation or activation of ECs. Treatment with oral bosentan alleviates VP in clinical patients with tourniquet‐induced VP. These findings suggest that targeting the endothelial‐neural axis and ET‐1/ETAR pathway may represent therapeutic strategies for VP.

## Graphical abstract

The authors develop mouse models by mimicking peripheral vascular diseases, combining multiple strategies and transgenic mouse lines, to demonstrate that ECs, but not macrophages or SMCs, play an active role mediated by endothelial ET‐1/neural ETAR signaling and beyond endothelial homeostatic angiocrine by driving protective pain through the endothelial‐neural circuit to alarm the CNS in vascular diseases.

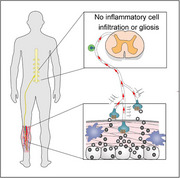

## 1 Introduction

The clinical manifestations of various vascular diseases are different; the most common symptom is not ischemia but unpleasant sensations, including pain or aching, throbbing, tightness, heaviness, swelling, muscle tiredness, itching, cramps, burning sensations, restless legs, tingling, and claudication.^[^ ^1^ ^]^ Many of these sensations are also involved in pain sensation, which is referred to as vascular pain (VP) and is associated with decreased quality of life, substantial morbidity, and high medical costs.^[^ ^1^, ^2^ ^]^ The term VP comprises a heterogeneous spectrum of vascular disease‐associated pain that is mainly characterized diagnostically by dysfunction of the blood vessels and symptomatically by pain. The classification and definition of VP are dependent on the type or anatomical location of the affected vessels. Based on vessel type, VP can be divided into venous pain, arterial pain, and microvascular pain;^[^ ^3^ ^]^ based on anatomical location, VP can be categorized as cardiovascular pain, cerebrovascular pain, peripheral VP (PVP), and visceral VP.^[^ ^4^ ^]^ However, except for angina pectoris and migraines,^[^ ^4^, ^5^ ^]^ other types of vascular pain have been overlooked by patients, clinicians, and pain researchers.

Endothelial cells (ECs) are multifunctional cells that form the inner layer of blood vessels and have a crucial role in vasculogenesis, angiogenesis, immunomodulation, nutrient uptake, and vasoreactivity, as well as various vascular diseases.^[^ ^6^ ^]^ Endothelin‐1 (ET‐1), an endogenous vasoactive peptide, is mainly derived from ECs and also produced by inflammatory cells, smooth muscle cells (SMCs), fibroblasts, glial cells, neurons, and other cells.^[^ ^7^ ^]^ ET‐1 is ubiquitously expressed and involved in a variety of physiological and pathological processes by binding two specific G‐protein‐coupled receptors, endothelin A receptor (ETAR) and B receptor (ETBR).^[^ ^8^ ^]^ These receptors are widely expressed in various systems throughout the body. In the peripheral nervous system, ETAR is expressed in nociceptive primary sensory neurons, whereas ETBR is mainly found in satellite glial cells.^[^ ^9^ ^]^ ET‐1 and ETAR play an important role in peripheral pain signaling, whereas the role of ETBR in pain is more varied.^[^ ^10^ ^]^ ETAR is expressed in the peripheral afferent nerve endings, nerve axons, and nociceptor cell bodies in the dorsal root ganglion (DRG), suggesting it is synthesized by neurons and transported to the nerve terminals.^[^ ^10^, ^11^ ^]^ ET‐1 also contributes to mechanical allodynia in a variety of pain conditions through ETAR.^[^ ^12^ ^]^ Therefore, we supposed that ETAR might play important roles in VP processing.

The pneumatic tourniquet is widely used during limb surgery in the clinic to minimize intraoperative bleeding, improve operative visualization that provides a cleaner field for cement penetration and fixation, and reduce intraoperative blood loss and operative time.^[^ ^13^ ^]^ However, tourniquets are frequently associated with severe tourniquet pain characterized by a gradual onset and dull, tight, aching sensation at the site of the tourniquet.^[^ ^13^, ^14^ ^]^ The potential mechanisms of tourniquet pain are thought to be related to nerve compression, nerve ischemia, muscle damage, ischemia–reperfusion injury, edema, metabolic changes or spinal receptive field expansion of nociceptors,^[^ ^15^ ^]^ as well as some potential pain signaling pathways;^[^ ^15^, ^16^ ^]^ however, the exact cause and pain pathways involved in tourniquet pain and methods for addressing this pain are poorly understood.^[^ ^17^ ^]^ We supposed that more microcirculation disturbance is involved in tourniquet pain, which is a type of PVP. It is well‐documented that nearly all peripheral blood vessels are accompanied by peripheral nerves, and larger blood vessels are innervated by these peripheral nerves, including nociceptive nerves.^[^ ^18^ ^]^ This configuration provides anatomical evidence for paracrine communication between blood vessels and the surrounding nociceptive afferents.

In this study, we mimicked lower extremity PVD to facilitate animal model establishment and phenotypical and mechanistic characterization of PVP. We provided solid evidence that primary sensory neuronal ETAR mediates experimental and clinical vascular pain through an endothelial‐neural axis. Activated ECs release ET‐1, which initiates the ET‐1/nociceptive ETAR signaling cascade, further excites the pain pathway, and leads to VP, and showed that blockade of this pathway alleviated VP in mouse models and clinical patients.

## 2 Results

### 2.1 VP Shows Distinct Behavioral Phenotypes Compared to Classic Pain Models

**Figure 1:**
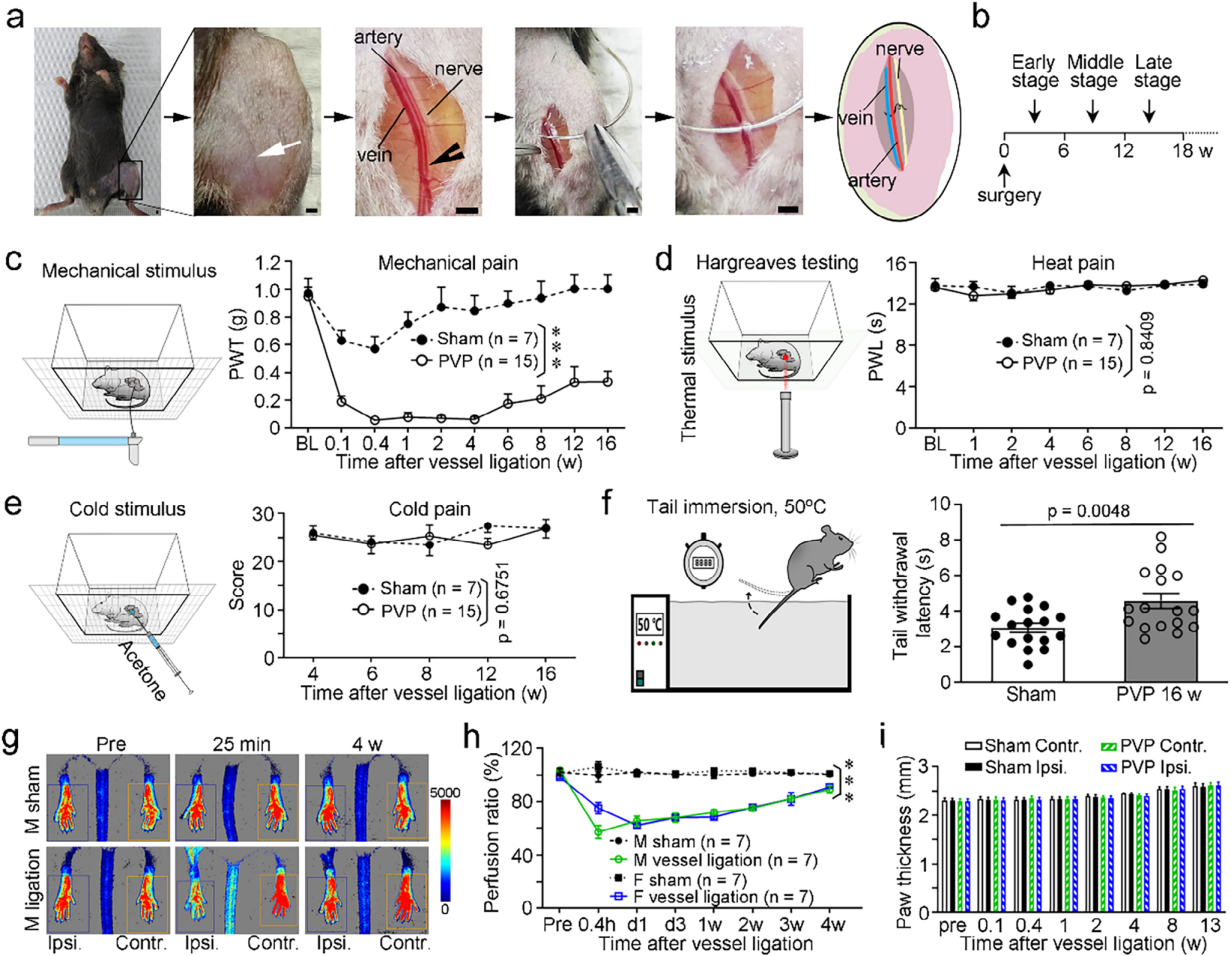
Behavioral phenotypes and blood flow alterations in the hindpaws of mice with PVP. a) Schematic of the vessel ligation‐induced PVP model. White arrows and blank arrowheads indicate the vessels. b) Arbitrary divisions into early, middle and late phases of PVP. c–e) Phenotypes of mechanical (c), heat (d), and cold (e) pain. f) A longer tail withdrawal latency in the late phase of mice with PVP. Schematic of tail immersion test (left) and a result of tail immersion test (50 °C) in mice with PVP at 16 weeks (right). g,h) Reduced blood perfusion in the vessel‐ligated paw and the subsequent recovery time course. Representative images indicating a reduction in blood flow (g) and a gradual recovery within four weeks (h). i) No edema in the ipsilateral paws, compared with that in the contralateral paws, of PVP mice. Scale bars, 1 mm. BL, baseline; contr., contralateral; F, female; M, male; ipsi., ipsilateral; pre, preadministration; PVP, peripheral vascular pain; PWL, paw withdrawal latency; PWT, paw withdrawal threshold. *n* = 7–15 (c–e), 17 (f), 7 (h) or 12 (i) mice. The data are presented as the mean ± SEM; ^***^ *p* < 0.001, statistical comparisons were conducted with two‐way ANOVA with Sidak's multiple comparisons post hoc test (c–e,h,i) or unpaired two‐tailed *t*‐test (f).

Leg vascular diseases are common in the clinic;^[^ ^19^ ^]^ consistently, pain models are often performed on their hind limbs to facilitate pain testing in rodents.^[^ ^20^ ^]^ Therefore, to mimic PVD of the lower limbs, we ligated the left great saphenous veins and saphenous arteries of mice to induce PVP (**Figure** **1**a; Figure S1, Supporting Information). When using an operating microscope, we observed that there was a distance (0.5–1.0 mm) between the blood vessels and the accompanying saphenous nerves (Figure S1, Supporting Information). We carefully ligated only the great saphenous veins and saphenous arteries while preserving the accompanying nerves. This was evidenced by the lack of activating transcription factor‐3 (ATF‐3), a marker of injured neurons, in lumbar (L)2‐6 DRG neurons; In contrast, sciatic nerve ligation led to the majority of neurons expressing ATF‐3 in the ipsilateral L2‐6 DRGs (Figure S2, Supporting Information). We first characterized pain phenotypes by subjecting these animals to behavioral tests to examine sensory responses to a range of mechanical (as measured by stimulation with von Frey hairs), heat (as measured by Hargreaves or hot‐plate testing) and cold (as measured by acetone or cold‐plate testing) stimuli applied to the glabrous skin of the ipsilateral hindpaws during the arbitrarily defined early, middle and late phases (Figure 1b). We found that mice with PVP exhibited a specific phenotype with profound mechanical hyperalgesia (Figure 1c) but not heat hyperalgesia in Hargreaves (Figure 1d) and hot‐plate tests (Figure S3a–d, Supporting Information) or cold allodynia in acetone drop (Figure 1e) and cold‐plate tests (Figure S3e,f, Supporting Information); this phenotype is distinct from those of chronic inflammatory pain and neuropathic pain that usually manifest with mechanical hyperalgesia, heat hyperalgesia and cold allodynia (Figure S4, Supporting Information).^[^ ^20^, ^21^ ^]^ Then, we aimed to confirm the heat pain threshold with the tail flick test (Figure 1f). Unexpectedly, PVP mice displayed heat hyposensitivity to tail immersion in hot water in the late phase, compared with sham control (4.59 ± 0.43 vs 3.08 ± 0.26 s; Figure 1f), suggesting that plasticity adaptation in the spinal cord was induced by ongoing pain signaling inputs. Moreover, we did not observe obvious spontaneous pain‐related behaviors (including paw licking, flinching, or jumping) during the whole PVP. In addition, the rotarod test (Figure S3g, Supporting Information) revealed impaired motor coordination in the late but not the early and middle stages in PVP mice (Figure S3h–k, Supporting Information), which is consistent with the clinical manifestations of patients with PVD of the lower limbs.^[^ ^22^ ^]^ In contrast, the open field test (Figure S5a, Supporting Information) shows an intact locomotor activity indicated by normal total traveled distance, mean speed, maximum speed, and a crossing number in PVP mice (Figure S5b–m, Supporting Information). These results indicate that mice with VP exhibit a distinct pain phenotype from canonical chronic inflammatory pain and neuropathic pain, and that the vessel‐ligated VP model, featuring pain and motor impairment in the late stage, shows similarities to the clinical manifestations observed in patients with PVD.^[^ ^22^, ^23^ ^]^

### 2.2 The VP Model Is Distinct from the Classic Hindlimb Ischemia Model in Lower Extremity Ischemia, Necrosis, and Walking Ability

The classic hindlimb ischemia (HLI) model is surgically induced by ligating the femoral artery and excising a 2‐ to 3‐mm portion of the artery,^[^ ^24^ ^]^ leading to a reduction of more than 70% in blood flow to the affected paw and usually leading to the loss of some digits due to ischemic necrosis. Therefore, the HLI model cannot be used to test pain behavior due to the ischemic impairment of leg ambulation and digital necrosis. However, in our model, we observed that the ipsilateral paws and the skin around the ligation site in the vessel‐ligated PVP mice looked normal during the whole PVP, namely without red swelling, pigmentation, pallor, ischemia, edema, skin or digital necrosis, self‐mutation or deformation in the ipsilateral paws and legs, compared with the contralateral counterparts of the PVP mice and the ipsilateral counterparts of the sham control mice (Figure S6a,b, Supporting Information). In contrast, HLI model mice exhibited skin pallor, flaking skin, digital necrosis, and deformation in the ipsilateral paws (Figure S7a,b, Supporting Information). We further utilized a laser speckle contrast perfusion imager to measure hindpaw blood perfusion just before surgical ligation and at the indicated time points following surgery and observed an immediate decrease of ≈40% in ipsilateral paw perfusion, which gradually improved over time, leading to nearly complete recovery within four weeks in the PVP mice (Figure 1g,h). We also estimated paw edema by calibrating the paw thickness with a caliper and found that the ipsilateral paws of PVP mice did not exhibit any edema, compared with the contralateral paws of PVP mice and the ipsilateral paws of sham control mice, during the whole PVP (Figure 1i). We also investigated the ambulation of HLI mice via open field and rotarod tests and found that these mice exhibited ambulation impairment during the first week of HLI (Figure S8a–k, Supporting Information). Notably, even during the first week of the most severe reduction in paw blood perfusion, motor function and locomotor activity were not affected in PVP mice, compared to those of sham control mice (Figures S5 and S8, Supporting Information). Combined with walking ability, which remained intact even in the early stage of obvious blood flow reduction, these results suggest that the PVP mouse model is distinct from the conventional HLI mouse model in terms of lower extremity ischemia and tissue necrosis.

Therefore, in the subsequent experiments, we evaluated VP by focusing on the time points after four weeks of ligation, as there was no occurrence of ischemic stress after four weeks.

### 2.3 ET‐1 Is Upregulated in ECs But Not Macrophages or SMCs during the VP Process

**Figure 2:**
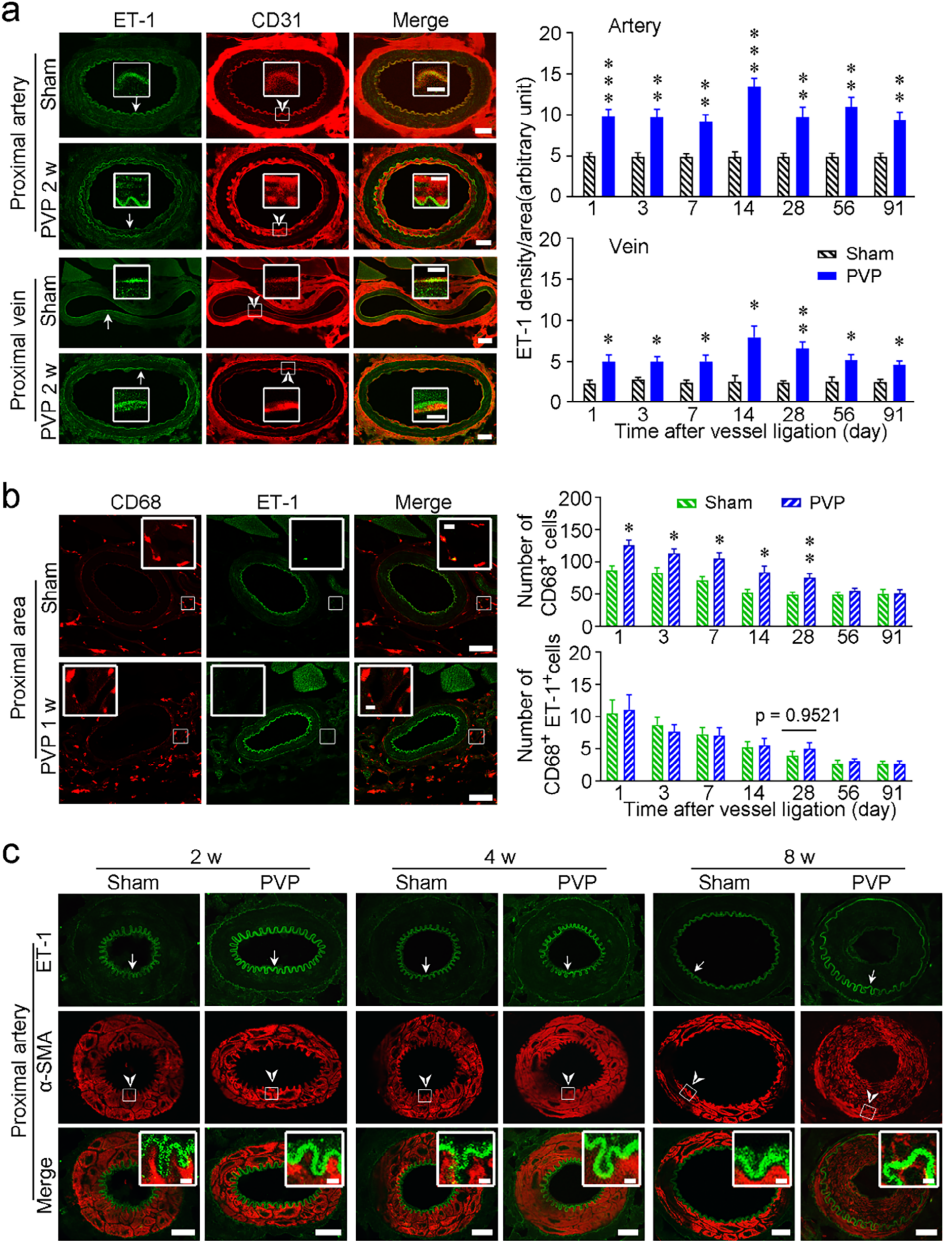
The expression of ET‐1 in the blood vessels and macrophages surrounding the tissues proximal to the ligated site during PVP. a) Increased expression of ET‐1 in ECs of the ligated arteries and veins proximal to the ligated sites in mice with PVP. Representative images indicating ET‐1 expression in ECs (left) and quantification of ET‐1 expression (right). Arrows indicate ET‐1 expressed in ECs; arrowheads indicate CD31 expressed in ECs. b) There was little expression of ET‐1 on the macrophages surrounding the tissues proximal to the ligated sites in the mice with PVP. Representative high‐magnification (40×) images indicating low ET‐1 expression in macrophages (left) and quantification of ET‐1 expression (right). c) Increased expression of ET‐1 was not detected in the SMCs of the ligated arteries and veins proximal to the ligated sites in mice with PVP. Scale bars, 20 µm (a, c), 50 µm (b), 10 µm (b, in boxes), and 2 µm (c, in boxes). α‐SMA, α‐smooth muscle actin; ET‐1, endothelin‐1; PVP, peripheral vascular pain. *n* = 12–19 sections from 5 mice. The data are presented as the means ± SEM. ^*^ *p* < 0.05, ^**^ *p* < 0.01, and ^***^ *p* < 0.001, statistical comparisons were conducted with two‐way ANOVA with Sidak's post hoc test.

The endothelin system has an important role in cardiovascular pathophysiology,^[^ ^25^ ^]^ and endothelin receptor antagonism is an established therapeutic strategy for pulmonary arterial hypertension (PAH) and scleroderma‐related digital ulceration in clinical practice.^[^ ^26^ ^]^ More importantly, ET‐1 has been proven to be involved in various pain processes and contributes to mechanical allodynia.^[^ ^10^, ^12^ ^]^ Here, we hypothesized that ET‐1, which is secreted primarily from ECs and is a potent vasoactive peptide that plays a prominent role in peripheral pain,^[^ ^10^, ^12^ ^]^ also plays an important role in VP. We next investigated the function of ECs by analyzing ET‐1 expression via immunofluorescence in the proximal regions closest to the ligation sites in PVP mice. We found that ECs expressed more punctate ET‐1 in the proximal arteries and veins in model mice throughout the PVP process, compared with sham control mice (**Figure** **2**a). Peak expression was observed at 2 weeks after vessel ligation (Figure 2a). Notably, ET‐1 expression shown at higher magnification clearly demonstrated its presence in ECs, as indicated by complete overlap with the CD31 (PECAM‐1, a pan‐EC marker)^[^ ^27^ ^]^ immunostaining results in sham control mice, whereas increased secretion of ET‐1 partly overlapped with the CD31 expression and partly diffused toward the media of the vessels (Figure 2a). In addition, ET‐1 with an expression that does not overlap with CD31, which is contiguous with ECs and SMCs, might also be secreted by SMCs or immune cells infiltrating the area around the ligated vessels; however, further experiments are needed to validate this possibility.

Macrophages are prominent immune cells in response to vessel injury or tissue ischemia, and they are remarkably involved in local inflammatory response and tissue regeneration.^[^ ^28^ ^]^ Next, we determined the infiltration of macrophages and their secreted ET‐1 levels near the proximal ligated vessels using an immunofluorescence test. CD68^+^ macrophages significantly infiltrated into the surgical area on day 1, gradually recovered, and returned to normal by day 28 (Figure 2b; Figure S9a, Supporting Information). Interestingly, some macrophages in the sham control mice expressed ET‐1; whereas only a few newly infiltrated and proliferated macrophages in PVP mice expressed ET‐1 (Figure 2b; Figure S9a, Supporting Information). We also investigated whether and to what extent local SMCs expressed ET‐1 during the PVP process and found that a small amount of scattered ET‐1 was expressed in smooth muscle (as indicated by α‐smooth muscle actin (α‐SMA) immunostaining) in the arteries and veins of the sham control mice, but there was no significant increase in the smooth muscle expression of ET‐1 in the mice with PVP (Figure 2c; Figure S9b, Supporting Information).

Additionally, we investigated the expression of ET‐1 in the tissues around the distal parts of the ligated vessels using immunofluorescence. We found increased expression of ET‐1 in ECs in the distal veins but not in those in the distal arteries, or macrophages or SMCs, as indicated by the overlap of ET‐1 with CD68 or α‐SMA (Figures S10a–c and S11a,b, Supporting Information). Interestingly, we found that only a few macrophages were present in the ligated blood vessels; however, none of them expressed ET‐1 (Figure S12a,b, Supporting Information).

**Figure 3:**
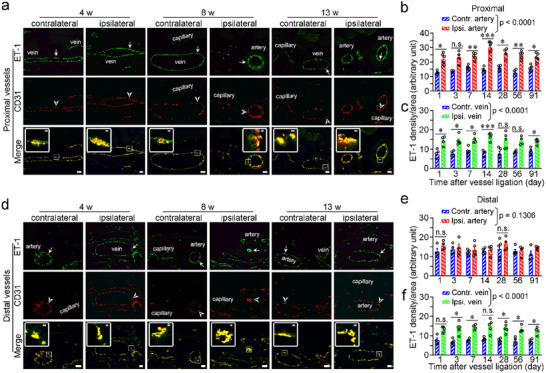
ET‐1 is primarily expressed by endothelial cells in the blood vessels and upregulated during PVP, as detected by in situ hybridization. a) Representative image indicating ET‐1 mRNA in ECs of the arteries, veins and capillaries proximal to the ligated sites in mice with PVP. b,c) Quantification of ET‐1 mRNA on ECs in the proximal arteries (b) and veins (c) around the vessel ligation site, compared to their contralateral counterparts. d) Representative images indicating ET‐1 mRNA in ECs of the arteries, veins, and capillaries distal to the ligated sites in mice with PVP. e,f) Quantification of ET‐1 mRNA on ECs in the distal arteries (e) and veins (f) around the vessel ligation site, compared to their contralateral counterparts. Arrows indicate the enlargement with ET‐1 expressed in ECs; arrowheads indicate the enlargement with CD31 expressed in ECs. Scale bars, 20 µm and 2 µm (in boxes). Contr., contralateral; ET‐1, endothelin‐1; ipsi., ipsilateral; n.s., non‐significant; PVP, peripheral vascular pain. *n* = 4–7 (b), 4–6 (c), and 4–5 (e,f) sections from 3 mice. The data are presented as the means ± SEM. ^*^ *p* < 0.05; ^**^ *p* < 0.01 and ^***^ *p* < 0.001; statistical comparisons were conducted with two‐way ANOVA with Sidak's post hoc test.

To further validate the upregulation of ET‐1 at both the transcriptional level and release level in VP mice, we conducted additional VP models for in situ hybridization and enzyme‐linked immunosorbent assay (ELISA) testing. In our RNAscope experiment, we observed that the ET‐1 probe and CD31 probe almost completely overlap in arteries, veins, and capillaries on the ipsilateral and contralateral sides of VP mice (**Figure** **3**a,d). Fluorescence quantitative analysis revealed that the expression of ET‐1 mRNA in the blood vessels on the ipsilateral side of VP mice was higher than that in the blood vessels on the contralateral side; the expression pattern closely resembled that observed with immunohistochemistry (Figure 3b,c,e,f). DAPI staining indicated that inflammatory cells immediately infiltrated the local sites of surgery (Figure S13a,b, Supporting Information); the infiltration patterns were similar to those observed in the immunofluorescence assay (Figure 2c; Figures S9–S12, Supporting Information). However, there were a few inflammatory cells expressing ET‐1 mRNA, although their expression was much lower compared to that of ECs (Figures S13c,d and S14a–d, Supporting Information). In the ELISA experiment, we surprisingly observed a higher concentration of ET‐1 in the heart blood of the VP mice compared to the sham surgery mice (Figure S14e, Supporting Information). As expected, we also observed a higher concentration of ET‐1 in the peripheral blood of the local ipsilateral sites compared to the local contralateral sites of the VP mice (Figure S14f, Supporting Information). These data from in situ hybridization and ELISA indicate that VP mice exhibit a higher level of ET‐1 in the systemic circulation and local blood circulation, which is mainly produced and released by ECs.

Taken together, these results suggest that ET‐1 secreted mainly from ECs possibly plays an important role in the VP process.

### 2.4 ET‐1/ETAR Signaling Plays an Important Role in the VP Process

**Figure 4:**
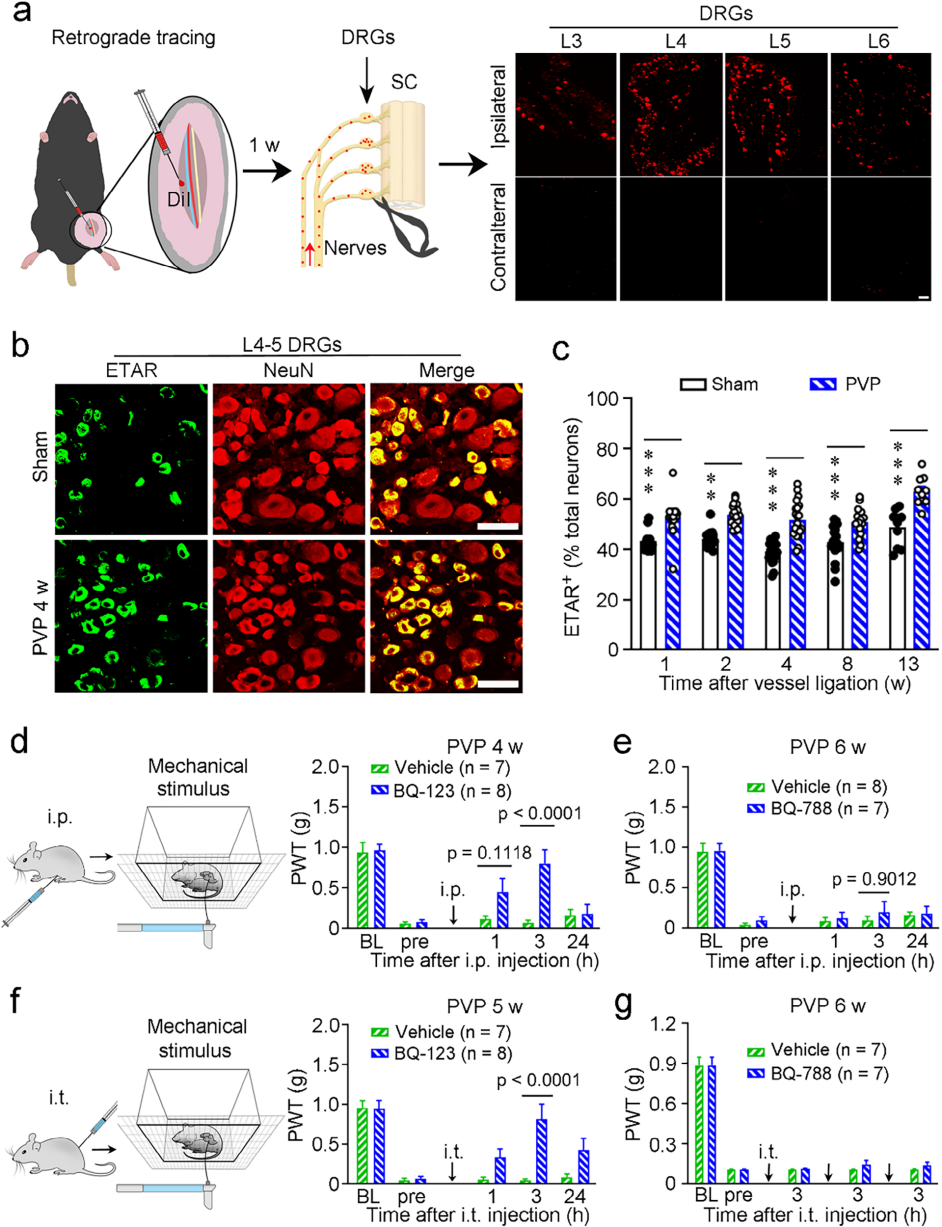
Activation of ETAR signaling during PVP. a) Schematic drawing and the results of retrograde tracing with DiI. Schematic of the tracing experiment (left) and representative images of DiI^+^ L3‐6 DRGs in the mice (right). b,c) Enhanced expression of ETAR in the L4‐5 DRGs of PVP mice. Representative images of ETAR expressed in DRG neurons (b) and quantification of ETAR expression (c). d–g) Systemic (d,e) or local (f,g) blockade of ETAR with BQ‐123 (d,f), but not ETBR with BQ‐788 (e,g), suppressed mechanical hyperalgesia in the early phase in PVP mice. Scale bars, 100 µm (a) or 50 µm (b). BL, baseline; ET‐1, endothelin‐1; ETAR, endothelin A receptor; ETBR, endothelin B receptor; i.p., intraperitoneal injection; i.t., intrathecal injection; L, lumbar; pre, preadministration; PVP, peripheral vascular pain; PWT, paw withdrawal threshold. *n* = 10–21 sections from 5 mice (b,c), or 7–8 mice (d–g). The data are presented as the means ± SEM. ^*^ *p* < 0.05, ^**^ *p* < 0.01, and ^***^ *p* < 0.001, statistical comparisons were conducted with two‐way ANOVA with Sidak's post hoc test.

Nociceptor terminals are the first neuronal components to detect and transduce noxious stimuli into propagating pain signals. Therefore, the primary sensory neurons residing in DRGs play a crucial role in pain processing.^[^ ^20^, ^21^, ^29^ ^]^ We further investigated the expression of ET‐1 and ETAR on nociceptors in mice with PVP. Best recognized for their sensory role, DRG neurons provide the information from the periphery through direct innervation of peripheral vessels. Using retrograde tracing with 1,1′‐dioctadecyl‐3,3,3′,3′‐tetramethylindocarbocyanine perchlorate (DiI) injected into the area around the ligated vessels, we identified that the L2‐6 DRGs^[^ ^30^ ^]^ innervated the affected area (**Figure** **4**a). We then measured the expression of ET‐1, ETAR, and several pain‐related immune molecules in the L4‐5 DRGs and spinal dorsal horn (SDH) using immunofluorescence.

ETAR expression was significantly increased on the ipsilateral L4‐5 DRG neurons of mice with PVP at 1, 2, 4, 8, and 13 weeks, compared with that of sham control mice (Figure 4b), although ET‐1 expression in DRG neurons was not altered (Figure S15a, Supporting Information); however, we did not investigate the expression of ET‐1 B receptor (ETBR), which is majorly expressed on satellites in the DRGs^[^ ^11^ ^]^ and has a less important role than ETAR in the peripheral pain process.^[^ ^31^ ^]^ Accordingly, we observed an upregulation of ETAR expression in the saphenous nerves accompanying the ligated blood vessels compared to the contralateral saphenous nerves (Figure S15b, Supporting Information). Then, we employed pharmacological blockade to assess the roles of ETAR and ETBR in VP. Systemic (intraperitoneal) or local (intrathecal; targeting cells in the spinal cord and DRGs) administration of BQ‐123, a specific blocker of ETAR, almost completely reversed the established mechanical hyperalgesia, returning the mechanical pain threshold to the baseline level, in the early phase (Figure 4d,f); however, this blocking effect decreased in the middle and late phases (Figure S15c,d, Supporting Information). In contrast, systemic or local administration of BQ‐788, a specific blocker of ETBR, did not affect the established mechanical hyperalgesia (Figure 4e,g; Figure S15e,f, Supporting Information). Combined with the common understanding that ETBR expressed in glial cells in DRGs^[^ ^11^, ^31^ ^]^ does not play an important role in other pain processes,^[^ ^31^ ^]^ we supposed that peripheral neural ETBR possibly is not critically involved in the VP process.

We further confirmed the role of neural ETAR by generating conditional knockout (CKO) mice in which ETAR was knocked out in primary nociceptive neurons of DRGs^[^ ^20^, ^29^, ^32^ ^]^ (SNS^cre^/ETAR^f/f^ or Na_v_1.8^cre^/ETAR^f/f^ mice; Figure S16a, Supporting Information), which was validated by immunofluorescence (Figure S16b, Supporting Information). CKO mice displayed partial impairment of mechanical hyperalgesia (indicated by paw withdrawal threshold) and allodynia (indicated by frequency) development after vessel ligation compared with ETAR^f/f^ mice (referred to as wild type (WT); Figure S16c,d, Supporting Information). In addition, we also confirmed the actions of the neural ET‐1 using Na_v_1.8^cre^/ET‐1^f/f^ and advillin^cre^/ET‐1^f/f^ CKO mice (Figure S16e, Supporting Information) and found that conditional deletion of ET‐1 in nociceptors (Na_v_1.8‐Cre) or primary sensory neurons (advillin‐Cre) did not attenuate PVP induced by vessel ligation (Figure S16f, Supporting Information). We also sought to investigate the effects of ET‐1 under the conditional knockout of ET‐1 in ECs using Tek‐Cre (strain 0 04128) and Tie‐Cre (strain 0 08863) strains. Unfortunately, we were unable to obtain homogeneous Tek^cre^/ET‐1^f/f^ or Tie2^cre^/ET‐1^f/f^ cubs due to the deletion of ET‐1 in ECs, hindering their embryo development. However, we were able to obtain the heterogeneous Tek^cre^/ET‐1^f/−^ cubs which developed normally. But, there was no noticeable difference in VP in Tek^cre^/ET‐1^f/‐^ mice compared to the ET‐1^f/f^ VP control mice (Figure S17a, Supporting Information). We further included the Cdh5‐Cre/ERT2 strain in which the Cre promoter will be expressed under tamoxifen induction. We generated Cdh5^cre^/ET‐1^f/f^ mice, and after tamoxifen induction, the adult mice exhibited conditional deletion of ET‐1 in ECs (Figure S17b, Supporting Information). Remarkably, CDH5^cre^/ET‐1^f/f^ mice exhibited a partial impairment of the VP process compared with ETAR^f/f^ mice after tamoxifen induction (Figure S17c, Supporting Information). These data suggest that ET‐1 is secreted mainly from ECs, but not from sensory neuronal ET‐1, and sensory neuronal ETAR play pivotal roles in the process of VP.

**Figure 5:**
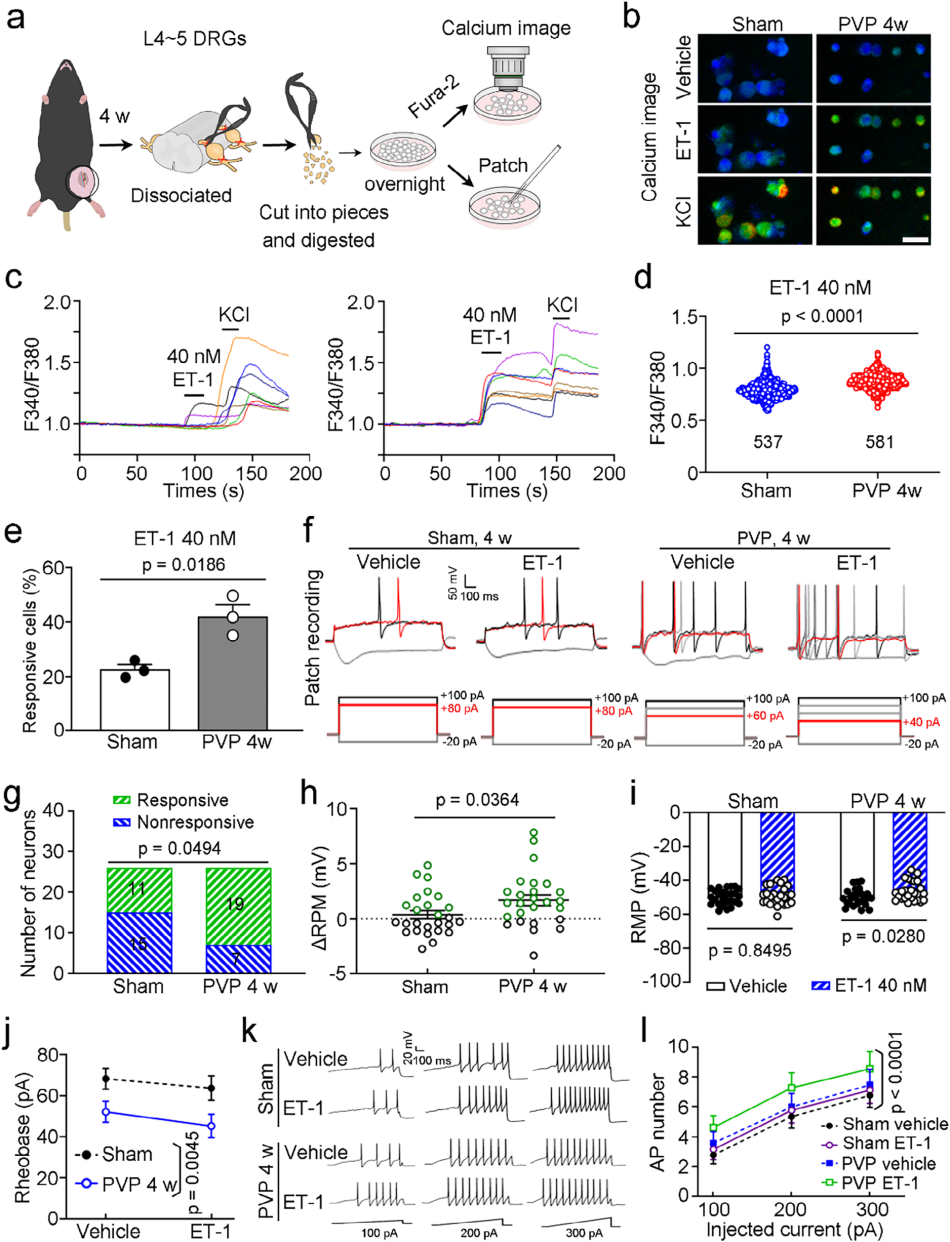
A higher sensitivity in primary sensory neurons of mice with PVP. a–e) More sensitive to ET‐1 in dissociated L4‐5 DRG neurons from mice with PVP than those from sham control mice measured by calcium imaging (b–e) for the degree of calcium influx into the neurons (b–d) and the percentage of responsive cells from three mice (e). Schematic of calcium imaging and patch recording for dissociated DRG neurons (a), representative images (b), and time course (c) of calcium influx when neurons were exposed to ET‐1 or KCl. f–l) Increased sensitivity to ET‐1 in dissociated L4‐5 DRG neurons from mice with PVP compared with those from sham control mice, measured by patch clamp recordings for responsive neuron number (g), alteration of the RMP of the neurons (h), the RMP of the neurons before and after ET‐1 prefusion (i), the rheobase evoking an AP in the neurons (j) and the firing AP number (l). f) Representative traces of action potentials (top) evoked by step current injections; the traces in red represent the APs evoked by the rheobase current (bottom). k) Representative traces of APs evoked by ramp current injections. Scale bar, 25 µm. AP, action potential; ET‐1, endothelin‐1; DRGs, dorsal root ganglia; L, lumbar; RMP, resting membrane potential. *n* = 537–538 neurons from 3 mice (b‐d), 3 mice (e), or 26 neurons from 4–6 mice (g–l). The data are presented as the means ± SEM. Statistical comparisons were conducted with an unpaired two‐tailed *t*‐test (d,e,h), chi‐square test (g), or two‐way ANOVA with Sidak's post hoc test (i,j,l).

To investigate the excitability of nociceptors in response to ET‐1 during VP, we performed calcium imaging and whole‐cell current‐clamp recordings in dissociated L4‐5 DRG neurons from mice with PVP and mice that underwent the sham operation at 4 weeks after vessel ligation (**Figure** **5**a). Acute incubation with ET‐1 evoked an increase in intracellular calcium influx that was more pronounced in neurons from mice with PVP than in those from sham control mice (Figure 5b–e). Out of a total of 537 neurons (179 ± 32.51) from 3 sham control mice, 123 neurons (41.0 ± 9.64) responded to ET‐1; in contrast, out of a total of 581 neurons (193.7 ± 26.3) from 3 mice with PVP, 246 neurons (82 ± 14.93) responded to ET‐1 (22.6 ± 1.8% vs 42.3 ± 4.2%; Figure 4e). All neurons responded to the high concentration of KCl, indicating that they were living cells. Importantly, the neurons that responded to ET‐1 were predominantly small‐diameter DRG neurons (Figure 5b), consistent with our histological findings (Figure 3b).

We then performed whole‐cell patch‐clamp recording on small‐diameter (< 25 µm) DRG neurons from sham and PVP model mice (Figure 5f). Out of a total of 26 neurons from 4 sham control mice, 11 neurons responded to ET‐1 with an increased number of spikes; in contrast, out of a total of 26 neurons from 6 mice with PVP, 19 neurons responded to ET‐1 with an increased number of spikes (Figure 5g,h). The resting membrane potential of the neurons exposed to ET‐1 was more depolarized than that of the neurons exposed to vehicle in the PVP mice; however, this difference was not observed in the sham control mice (Figure 5i). Acute incubation with ET‐1 reduced the injection currents required to elicit an action potential in neurons from both sham control mice and mice with PVP; however, the stimulating injection current was lower in neurons from mice with PVP than in those from sham control mice, as indicated by a lower rheobase (Figure 5j). Exposure to ET‐1 increased the action potential firing of DRG nociceptors isolated from both sham control mice and mice with PVP; of note, neurons from mice with PVP exhibited higher sensitivity, as indicated by more the number of spikes (Figure 5k,l). The above results suggest that DRG neurons from mice with PVP are more sensitive to ET‐1 than those from sham control mice.

Together, these data suggest that ECs directly communicate with vascular or nearby tissue sensory nerve fibers through ET‐1, which is sufficient to drive nociceptive responses through ETAR expressed on primary nociceptive fibers.

### 2.5 VP Is Distinct from Classic Forms of Pain in Gliosis

Next, we investigated the expression of the pain‐related immune features in the L4‐5 DRGs and SDH using an immunofluorescence test. Neither significantly increased ionized calcium‐binding adapter molecule 1 (IBA‐1) immunoreactivity nor significantly increased glial fibrillary acidic protein (GFAP) immunoreactivity on the ipsilateral L4‐5 DRGs or SDH was observed (Figures S18a–c and S19a,b, Supporting Information), suggesting that macrophage infiltration into the ipsilateral L4‐5 DRGs and microglial activation and proliferation in the ipsilateral SDH (both of which were assessed by IBA‐1 immunoreactivity and confirmed by CD68 immunoreactivity (Figure S18b,d, Supporting Information), in which exhibited CD68 almost co‐expression with IBA‐1 in the DRG neurons and largely colocalization with IBA‐1 in the SDH) did not occur; similarly, satellite cell reactivity in the DRGs and astrocyte reactivity in the SDH (both of which were assessed by increased GFAP immunoreactivity) on the ipsilateral side did not occur too (Figure S19a,b, Supporting Information), which is distinct from chronic neuropathic pain and chronic inflammatory pain, as inflammatory cell infiltration into and gliosis in the innervating DRGs and spinal cord are critical features of other pathological pain states.^[^ ^33^ ^]^ Based on the above observations, we supposed that the VP is a new form of pain that exhibits only mechanical hyperalgesia but not heat hyperalgesia or cold allodynia and does not involve gliosis or neuroinflammatory processes.

### 2.6 ECs Regulate VP Mediated by ET‐1/ETAR Pathway through the Endothelial‐Neural Circuit

**Figure 6:**
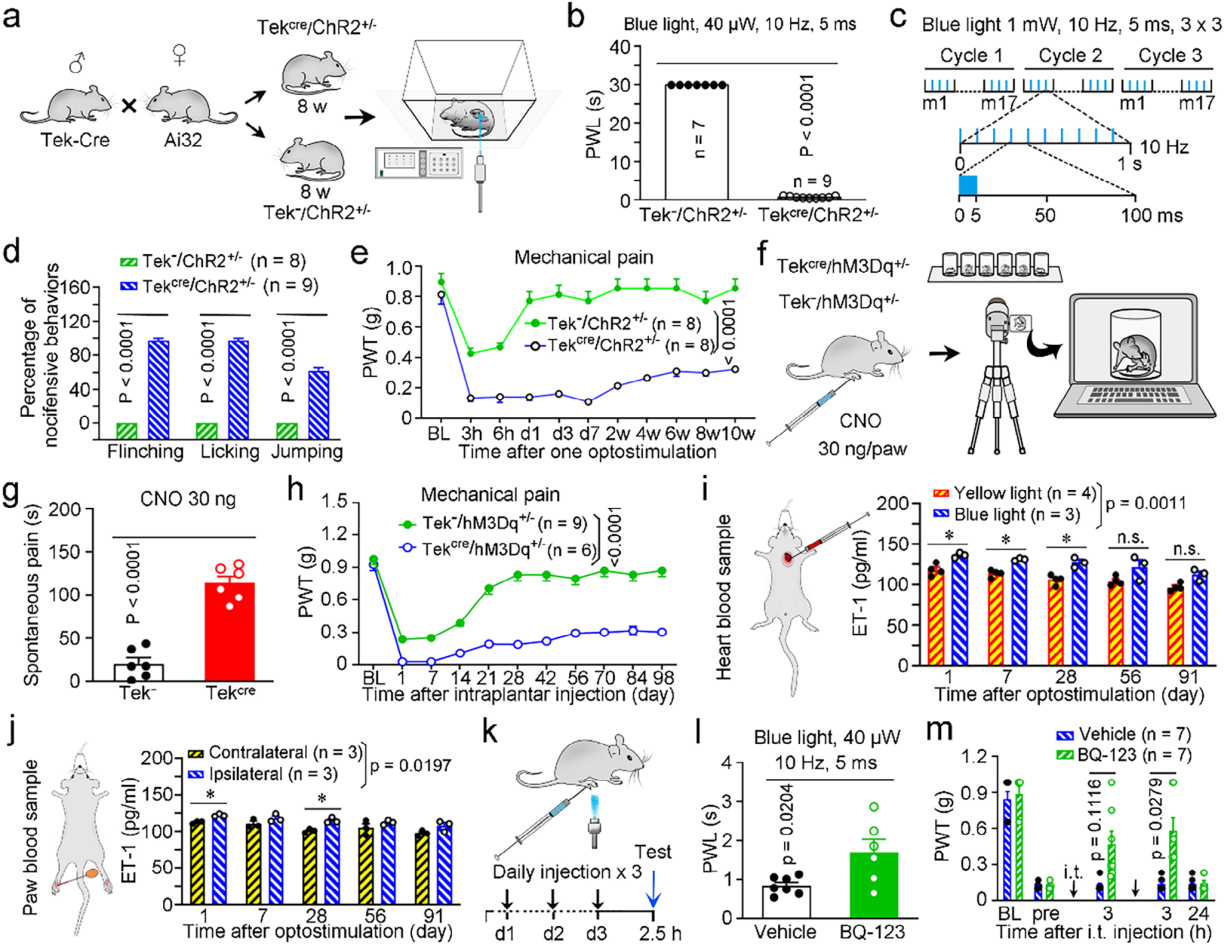
Acute and chronic pain‐like behaviors triggered by optogenetic and chemogenetic stimulation of ECs. a) Schematic of Tek^cre^/ChR2^+/−^ mice generation by crossing EC‐dependent promoter (Tek‐Cre) mice with Ai32 mice and optogenetic manipulations. b) Acute avoidance response evoked by blue light stimulation indicated by the PWL. c,d) Schematic of the experimental protocol for repeated and strong optogenetic stimulation (c) and evoked acute nocifensive responses (d) in Tek^cre^/ChR2^±^ mice. e) A single weak optogenetic stimulation‐evoked long‐lasting mechanical hyperalgesia indicated by the PWT in Tek^cre^/ChR2^±^ mice. f) Schematic of the selective chemogenetic activation of ECs upon CNO administration and the behaviors of Tek^cre^/hM3Dq^+/−^ mice recorded via a video system. g,h) Acute avoidance responses (g) and long‐lasting mechanical hyperalgesia (h) evoked by a single local administration of CNO in Tek^cre^/hM3Dq^+/−^ mice. i,j) Quantification of serum ET‐1 concentration in heart blood (i) or paw local blood samples (j) measured by ELISA for optogenetic mice following ECs activation. k–m) Schematic of the experimental protocol (k), local preadministration of BQ‐123 preventively increased the PWL in Tek^cre^/ChR2^+/−^ mice under blue light stimulation (l) and local administration of BQ‐123 inhibited the established long‐lasting mechanical hyperalgesia (2 w) induced by blue light stimulation in Tek^cre^/ChR2^+/−^ mice (m). BL, baseline; ChR2, channelrhodopsin‐2; CNO, clozapine‐N‐oxide; ET‐1, endothelin‐1; i.t., intrathecal injection; hM3Dq, Gq‐coupled human M3 muscarinic receptor; m, mouse; n.s., non‐significant; pre, preadministration; PVP, peripheral vascular pain; PWL, paw withdrawal latency; PWT, paw withdrawal threshold; Tek^−^/, Tek^−^/hM3Dq^+/−^; Tek^cre^/, Tek^cre^/hM3Dq^+/−^. *n* = 7–9 (b), 8–9 (d), 8 (e), 6 (g, l), 6–9 (h), 5–6 (i) or 7 (m) mice. The data are presented as the means ± SEM; ^*^ *p* < 0.05, ^**^ *p* < 0.01, and ^***^ *p* < 0.001. Statistical comparisons were conducted with an unpaired two‐tailed *t*‐test (b,g,l) or two‐way ANOVA with Sidak's post hoc test (d,e,h‐j,m).

Based on our above observation, we hypothesized that activated ECs could directly evoke PVP prior to ischemic pain occurrence in vessel diseases. Although large‐vessel vasculitis rarely occurs in the skin, cutaneous vasculitis is common in medium‐ and small‐vessel vasculitis,^[^ ^34^ ^]^ suggesting that it is reasonable to experimentally manipulate the ECs of medium‐ and small‐vessels in the paw skin for testing pain behaviors in mice. Thus, to investigate whether peripheral ECs can directly crosstalk with the perivascular or adjacent primary sensory nerves, we adopted an optogenetic strategy^[^ ^35^ ^]^ utilizing transgenic mice engineered to express channelrhodopsin‐2 (ChR2) in ECs^[^ ^36^ ^]^ driven by the Tek (Tie2) promoter to specifically activate the peripheral ECs of skin vessels in the hind paws of adult transgenic mice (**Figure** **6**a). After one session of transdermal optostimulation (terminal intensity, 40 mW mm^−2^; pulse frequency, 10 Hz; illumination duration, 5 ms) with a 473‐nm laser (blue light), similar to the setting used in the Hargreaves test (Figure 6a), Tek^cre^/ChR2^+/−^ mice, but not Tek^−^/ChR2^+/−^ control mice exhibited a pain‐like avoidance response (paw withdrawal) before the 30 s cutoff (0.80 ± 0.08 vs 30.00 ± 0.00 s; Figure 6b); however, we did not find any pulse frequency‐dependent effects when blue light stimulating (Figure S20a, Supporting Information). As a control, stimulation with a 593‐nm laser (yellow light) did not evoke any pain‐like avoidance response (paw withdrawal) within the 30‐s cutoff in either the transgenic or the control mice (Figure S20b, Supporting Information).

We subsequently investigated whether optostimulation evokes spontaneous pain‐like behaviors.^[^ ^29a^ ^]^ After nine strong optostimulations (three cycles of three successive strong stimulations within 20 min; 1 mW mm^−2^, 10 Hz, and 5 ms) with blue light (Figure 5c), Tek^cre^/ChR2^+/−^ mice but not Tek^−^/ChR2^+/−^ control mice exhibited spontaneous pain‐like behaviors, including paw flinching (97.53 ± 2.47%), licking (97.53 ± 2.47%), and jumping (61.73 ± 3.76%; Figure 6d). Notably, we also found that Tek^cre^/ChR2^+/−^ mice but not Tek^−^/ChR2^+/−^ mice displayed persistent mechanical hyperalgesia, lasting at least ten weeks, after only one round of transdermal optostimulation (Figure 6e). However, we did not observe any reduction in blood flow or obvious edema in the optostimulated paws (Figure S20c–e, Supporting Information).

We further confirmed the above mechanism of nociception in activated ECs using a chemogenetic strategy based on designer receptors exclusively activated by designer drugs (DREADDs).^[^ ^37^ ^]^ We generated a Tek^cre^/Gq‐coupled human M3 muscarinic receptor (hM3Dq)^+/−^ transgenic strain (Figure S21a, Supporting Information) and administered clozapine‐N‐oxide (CNO)^[^ ^37^ ^]^ by intraplantar injection to the adult mice (Figure 6f). Video analysis revealed that Tek^cre^/hM3Dq^+/−^ mice exhibited more acute nocifensive behaviors (spontaneous pain‐like behaviors including flinching, licking, and jumping) than did Tek^−^/hM3Dq^+/−^ control mice (114.12 ± 8.05 s vs 20.97 ± 6.89 s; Figure 6g). Interestingly, Tek^cre^/hM3Dq^+/−^ mice, but not Tek^−^/hM3Dq^+/−^ control mice, also exhibited persistent mechanical hyperalgesia (lasting at least 12 weeks) evoked by filament stimulation after a single intraplantar injection of CNO (30 ng; Figure 6h). We also assessed paw perfusion and edema following chemogenetic‐evoked PVP and observed a brief reduction of ≈20% in the blood flow of the affected paws and a daily recovery after 10 or 30 ng CNO injection into the paws (Figure S21b–e, Supporting Information); similarly, the occurrence of paw edema was brief, with recovery within 6 h in Tek^cre^/hM3Dq^+/−^ mice, compared with Tek^−^/hM3Dq^+/−^ mice (Figure S21f,g, Supporting Information). Notably, the degree of paw edema in the females was less than that of the males in Tek^cre^/hM3Dq^+/−^ mice after 10 but not 30 ng CNO paw injection (Figures S20e and S21f, Supporting Information).

Chemogenetic stimulation‐activated EC‐evoked acute nocifensive behaviors (spontaneous pain) were further confirmed in Tek‐Cre mice injected with adeno‐associated viruses (AAVs) expressing the activating DREADD (AAV‐hM3Dq) into hindpaws (Figure S22a, Supporting Information) and then intraplantarly injected with 20 µg of CNO (Figure S22b, Supporting Information). The results of these experimental mice were validated by observing the robust expression of AAV‐hM3Dq‐coupled mCherry, ChR2 coupled enhanced yellow fluorescence protein (EYFP), and hM3Dq coupled mCherry in the blood vessels of the hindpaw skin of Tek^cre^/AAV, Tek^cre^/ChR2^+/−^ and Tek^cre^/hM3Dq^+/−^ mice, respectively (Figure S22c, Supporting Information). Thus, direct specific activation of local ECs by chemogenetic and optogenetic manipulation evokes acute and chronic pain‐like behaviors.

Next, we analyzed the serum concentration of ET‐1 using ELISA in Tek^cre^/ChR2^+/−^ and Tek^cre^/hM3Dq^±^ mice after chemogenetic or optogenetic manipulation, respectively. Notably, we found a higher concentration of ET‐1 in the heart blood of these Tek^cre^/ChR2^+/−^ and Tek^cre^/hM3Dq^±^ VP mice compared to their counterpart control mice (Figure 6i; Figure S22d, Supporting Information). Additionally, we also observed a higher concentration of ET‐1 in the local blood of the ipsilateral peripheral paws compared to that of the contralateral paws in Tek^cre^/ChR2^+/−^ VP mice (Figure 6j), but not in Tek^cre^/hM3Dq^±^ VP mice (Figure S22e, Supporting Information). We further estimated the mediation of ET‐1 in the optostimulation‐induced PVP model using pharmacological methods. Intraplantar administration of BQ‐123 (10 nmol in 10 µL of saline) for three consecutive days resulted in a longer paw withdrawal latency after optostimulation than vehicle control treatment (1.71 ± 0.33 s vs 0.85 ± 0.27 s) in Tek^cre^/ChR2^+/−^ mice (Figure 6k,l), suggesting a preventive action of BQ‐123 on optostimulation‐evoked acute pain‐like behavior. Moreover, intrathecal injection of BQ‐123 (5 nmol in 5 µL of saline) partially relieved optostimulation‐induced mechanical hyperalgesia at 2 and 13 weeks after blue light stimulation in Tek^cre^/ChR2^+/−^ mice, compared with vehicle control treatment (Figure 6m; Figure S23a–c, Supporting Information).

We also confirmed the CKO effect of ET‐1 in ECs using Cdh5^cre^/hM3Dq^+/−^/ET‐1^f/f^ chemogenetic mice in which the Cdh5‐Cre/ERT2 promoter is induced after tamoxifen induction. Video analysis revealed that Cdh5^cre^/hM3Dq^+/−^/ET‐1^f/f^ mice exhibited less acute nocifensive behaviors than did Cdh5^cre^/hM3Dq^±^ mice after a single intraplantar injection of CNO (45.58 ± 6.05 s vs 126.48 ± 8.19 s; Figure S23d, Supporting Information). Accordingly, Cdh5^cre^/hM3Dq^+/−^/ET‐1^f/f^ CKO mice also exhibited a partial impairment in following persistent mechanical hyperalgesia after a single intraplantar injection of CNO, compared with that in Cdh5^cre^/hM3Dq^±^ mice (Figure S23e, Supporting Information). These results indicate that stimulation of ECs is sufficient to evoke short‐term and long‐term pain‐related behaviors and that robust communication mediated by the ET‐1 signal occurs between ECs and primary sensory afferents.

Finally, we investigated the expression of ETAR in Tek^cre^/ChR2^+/−^ mouse DRG neurons after blue light stimulation and found that ETAR expression was significantly increased in the ipsilateral L4‐5 DRG neurons of Tek^cre^/ChR2^+/−^ mice with PVP after optostimulation than in those of ChR2^+/−^ control mice (Figure S24a,b, Supporting Information). Accordingly, the expression of ETAR on the ipsilateral sciatic nerve was also significantly higher compared to the contralateral counterpart in Tek^cre^/ChR2^+/−^ mice with PVP following optostimulation (Figure S24c,d, Supporting Information). Consistent with the increased expression of ETAR in the L4‐5 DRGs of vessel‐ligated mice (Figure 4b,c; Figure S15b, Supporting Information) and the behavioral results of pharmacological blockade of the receptor with BQ‐123 (Figure 6l,m; Figure S23a–c, Supporting Information). We also assessed the activity of ETBR expressed in neural tissue or skin using a pharmacological approach. We found that intrathecal injection of BQ‐788 (5 nmol in 5 µL of saline) for three consecutive days did not affect the established mechanical hyperalgesia within 13 weeks after optostimulation, compared with the vehicle control treatment (Figure S25a,b, Supporting Information); however, intraplantar pretreatment with BQ‐788 (10 nmol in 10 µL of saline) led to a shorter paw withdrawal latency after optostimulation than did vehicle control treatment in Tek^cre^/ChR2^+/−^ mice (0.32 ± 0.08 s vs 0.60 ± 0.10 s; Figure S25c,d, Supporting Information). These results supported our hypothesis that the peripheral neural ETBR is not critically involved in the VP process, suggesting that blocking ETBR expressed in the skin causes an opposite behavioral outcome from blocking ETAR, namely, the activation of ETBR limits ET‐1‐induced VP, which is worthy of further study.

### 2.7 Blocking the ETAR Signaling Ameliorates Clinical VP

**Figure 7:**
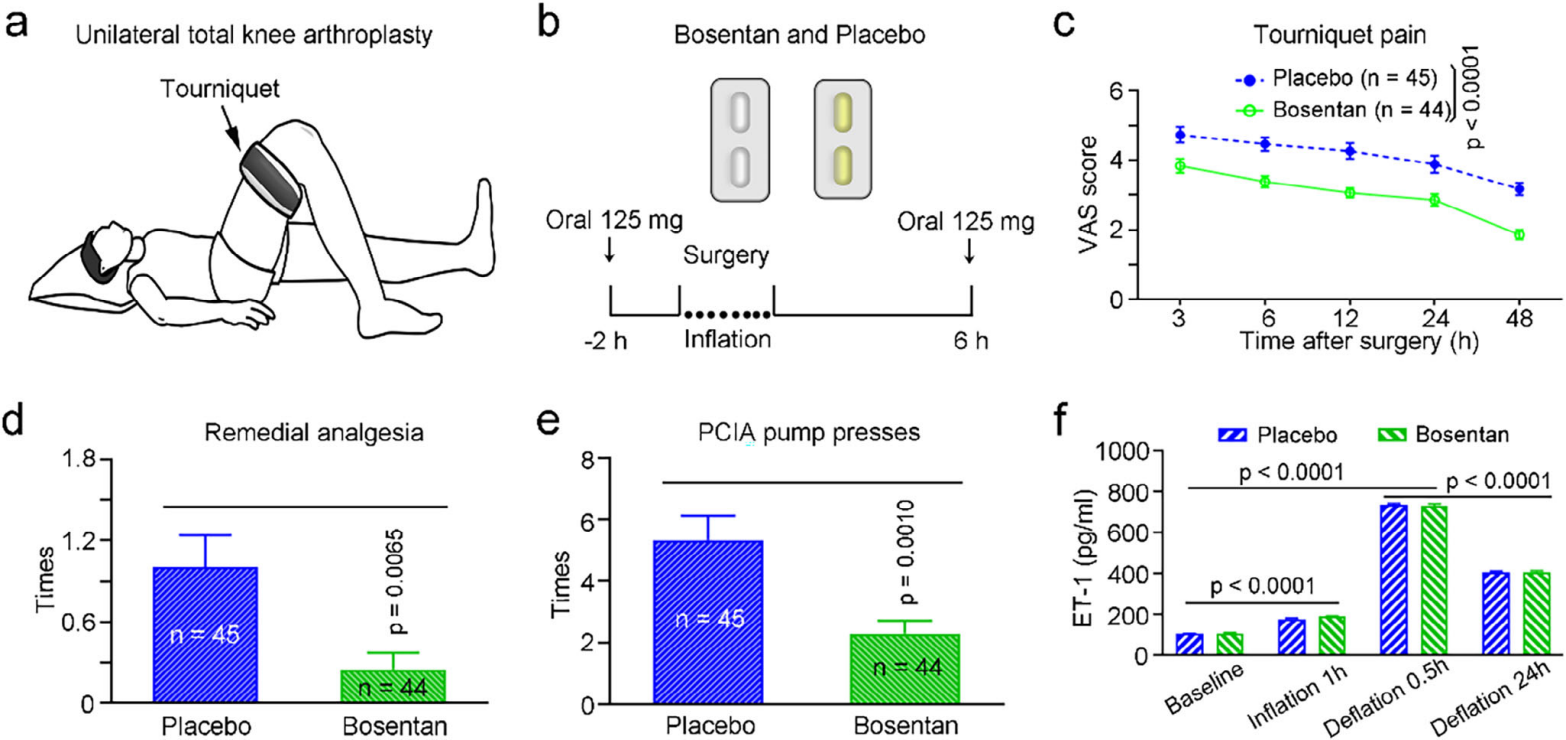
Antinociception by blocking the ET‐1 signal in clinical patients with tourniquet pain. a,b) Schematic of the experimental protocol in knee arthroplasty or lower leg fracture patients with a tourniquet (a) and oral administration of bosentan (b). c–e) Blocking ET‐1 signaling by oral administration of bosentan reduced tourniquet pain (c), the duration of remedial analgesia was required (d), and the number of PCIA pump presses (e) in knee arthroplasty or lower leg fracture patients after surgery. f) Serum ET‐1 levels in bosentan‐treated and placebo‐treated patients before, during, and after bandaging. ET‐1, endothelin‐1; PCIA, patient‐controlled intravenous analgesia; VAS, visual analog scale. *n* = 44–45 patients. The data are presented as the means ± SEM, statistical comparisons were conducted with two‐way ANOVA with Sidak's post hoc test (c,f) or unpaired two‐tailed *t*‐test (d,e).

Tourniquets are commonly used to provide a nearly bloodless field by minimizing surgical bleeding and, thereby, facilitating orthopedic surgery under either general, local, or regional anesthesia. However, tourniquet inflation is usually associated with intraoperative and postoperative pain, referred to as tourniquet pain.^[^ ^38^ ^]^ In addition, patients receiving sufficient surgical block with regional anesthesia can still experience postoperative tourniquet pain.^[^ ^38^ ^]^ Considering intraoperative and postoperative tourniquet‐related pain as acute and subacute PVP, we hypothesized that microcirculation ECs may play an important role in this process through the release of ET‐1. To test this hypothesis, we recruited 89 patients (age: 59.0 ± 1.2 years) undergoing unilateral total knee arthroplasty (75 patients) or fracture repair of lower legs (14 patients) under general anesthesia at the Affiliated Hospital of Xuzhou Medical University between September 2017 and March 2019 and treated them with either a placebo or bosentan, a mixed ETAR/ETBR antagonist, which is an available treatment for PAH (**Figure** **7**a,b). Bosentan was used preventively two hours before surgery and therapeutically six hours after surgery in 44 patients, and it was found that postoperative tourniquet‐related pain, as the primary outcome, was profoundly ameliorated in bosentan‐treated patients compared with placebo‐treated control patients, as measured by estimated visual analog scale (VAS) scores (Figure 7b,c). In addition, the incidence of remedial analgesia and the number of patient‐controlled intravenous analgesia (PCIA) pump uses, the secondary outcomes, were significantly reduced in bosentan‐treated patients than in control patients (Figure 7d,e). Notably, ET‐1 levels were robustly upregulated in patient plasma both during and after bandaging, with no difference between the groups (Figure 7f), suggesting that use of tourniquets activates local ECs, which results in the release of a large amount of ET‐1, and that prophylactic treatment with bosentan does not affect patient plasma ET‐1 levels. These clinical data support our hypothesis that an endothelial‐neural communication mechanism for modulating VP through the ET‐1/ETAR pathway and indicate that blocking the ET‐1 signal is beneficial for VP treatment.

## 3 Discussion

**Figure 8:**
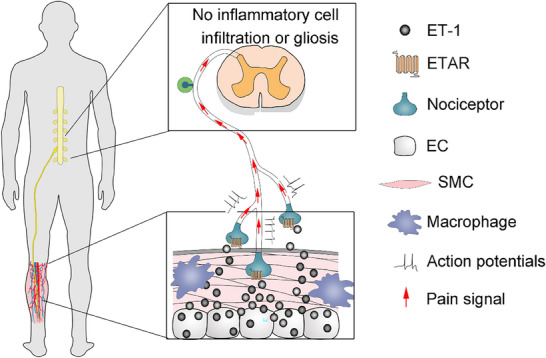
The hypothesis indicating the endothelial‐afferent‐nociceptor neuron‐spinal cord circuit via ET‐1/ETAR pathway is involved in VP. Schematic indicating the endothelial‐afferent‐nociceptor neuron axis mediated by ET‐1 secreted by ECs but not SMCs or macrophages, and neural ETAR signaling involved in VP. ECs, endothelial cells; ET‐1, endothelin‐1; ETAR, endothelin A type receptor; SMCs, smooth muscle cells; VP, vascular pain.

Although two recent elegant studies have suggested that the small blood vessel events in the local site of nerve injury or the innervating DRGs are implicated in the development of marked allodynia and aversion to gentle touch, or spontaneous pain in chronic neuropathic pain.^[^ ^27^, ^39^ ^]^ In the present study, our focus was on the VP associated with vascular diseases themselves. We demonstrated that ECs have an active role mediated by ET‐1/ETAR signaling and beyond homeostatic angiocrine^[^ ^40^ ^]^ by driving protective pain through the endothelial‐neural axis to alarm the CNS in vascular diseases. We classified VP as a new type of pain that is distinct from other classic types of pain; and identified and characterized ET‐1/ETAR‐mediated vascular‐neuronal communication at the EC level, but not SMC or macrophage, and primary sensory afferent levels. In addition, we revealed that the ET‐1/ETAR pathway is involved in the VP process and that blocking the ETAR signal significantly ameliorates VP in experimental animals and clinical patients (**Figure** **8**).

A broad and heterogeneous group of vascular diseases can be accompanied by various painful symptoms, and in fact, VP in the pain field remains largely unclear. Here, we focused on PVP, as PVD is prevalent worldwide; accordingly, the pain model is usually performed on the hind legs of rodents. Therefore, we developed mouse models of acute and chronic PVP, which will facilitate pain research.^[^ ^20e^ ^]^ Vessel‐ligated PVP model exhibited long‐lasting mechanical hyperalgesia, but not spontaneous pain or thermal hyperalgesia (Figure 1; Figure S3, Supporting Information), without inducing inflammatory cell infiltration or gliosis (a critical contributor to many pathological pain states including chronic neuropathic pain, inflammatory pain, visceral pain, and cancer pain)^[^ ^33^, ^41^ ^]^ in the innervating DRGs or spinal cord (Figures S18 and S19, Supporting Information), suggesting that VP is a new type of pain. Although several animal models of thrombus‐induced ischemic pain were well established,^[^ ^42^ ^]^ we aimed to precede overt the ischemic pain that is the last stage of many types of VP and investigate the endothelial‐neural communicational mechanisms underlying the initiation and development of VP, because many VP such as drug‐induced acute VP,^[^ ^43^ ^]^ vascular headache (including migraine)^[^ ^21^, ^44^ ^]^ and others, involve mechanisms rather than ischemia and hypoxia, which is supported by the evidence that ligation of the superficial vessels in the hind legs of PVP mice elicited only a moderate reduction in hindpaw perfusion in the early phase, without any ischemic stress or necrosis (Figure 1; Figures S6 and S7, Supporting Information) and that optogenetic or chemogenetic activation of ECs evoked robust PVP but not an obvious reduction of paw perfusion (Figure 6; Figures S20 and S21, Supporting Information). This study has expanded our understanding of VP beyond an ischemic pain perspective.

Notably, the pain phenotype of PVP is characterized by obvious mechanical pain, without the presence of heat or cold pain. This is similar to the phenotype observed in animal models of thrombus‐induced peripheral ischemic pain and central post‐stroke pain.^[^ ^42^, ^45^ ^]^ We also observed that PVP mice did not exhibit cold allodynia, which is in line with the clinical manifestation of the patients with PVP living in a cold climate not experiencing increased pain during colder weather. More interestingly, PVP mice displayed heat hyposensitivity during tail immersion in hot water in the late phase of PVP (Figure 1f), suggesting that plasticity adaptation in the SC may have been induced by ongoing pain signaling. Our findings from the tail immersion tests were not consistent with the paw withdrawal latency data, possibly due to the tail flick reflex being limited to the spinal circuit, whereas the paw withdrawal reflex involves brain circuits. Another possibility is that the tail flick reflex may be more sensitive than the paw withdrawal reflex, which warrants further investigation.

We also presented evidence showing that the PVP model is distinct from the HLI model in terms of the degree of ischemia (including blood perfusion of the affected paw and skin injury), tissue necrosis and ambulation impairment (Figure 1; Figures S3–S5, Supporting Information). Possibly, the phenotype of PVP mice did not induce inflammatory cell infiltration or gliosis in the innervating DRGs or spinal cord, largely because of its lower degree of ischemic, compared to thrombus‐induced peripheral ischemic pain and central post‐stroke pain. Furthermore, PVP mice showed reduced motion abilities in the later stages, similar to the symptomatic claudication observed in elderly patients with PVDs.^[^ ^4^, ^19^, ^22^, ^23^, ^46^ ^]^

We directly activated ECs in the mouse hindpaw to establish acute PVP models that exhibited spontaneous pain behaviors and chronic PVP models that exhibited lasting mechanical hyperalgesia using chemogenetic and optogenetic approaches (Figures 5 and 6). These models provide the opportunity to study the transmission of pain signals from ECs to primary sensory neurons and to test the mechanisms underlying the initiation and maintenance of VP. Notably, we emphasized that PVP induced by EC activation exhibited similar phenotypic and molecular characteristics to vessel‐ligated PVP. However, while we employed three EC‐derived Cre strains to validate the specific activation of ECs, it is important to exercise caution in explaining the results, as none of the above Cre strains are exclusively expressed in ECs.

Interestingly, a recent elegant study indicated that small blood vessels provide a scaffold and guide for sprouting nociceptors in denervated tissue after nerve injury, which is implicated in the development of marked allodynia and aversion to gentle touch in chronic neuropathic pain.^[^ ^39^ ^]^ Another recent elegant study showed that triggers of neuropathic spontaneous pain and clustered firings are caused by dynamic movements of blood vessels within the nerve‐injured DRGs, detected by Piezo2 receptors in adjacent neurons.^[^ ^27^ ^]^ These studies suggest that the peripheral endothelial‐neural axis may play an essential role in neurovascular communication and may also be involved in chronic neuropathic pain. In contrast, the findings of the study highlighted the pain caused by vascular diseases themselves. When vascular diseases occur, the local ECs of the lesion are first activated by the lesion, leading to changes in blood flow. The distal ECs are then activated by shear stress,^[^ ^47^ ^]^ as evidenced by the release of ET‐1 detected by local serum at the ligation site and in the systemic serum of the heart (Figures S14 and S22, Supporting Information). This suggests that ET‐1 is involved in physiological and pathological processes through autocrine, paracrine, and remote endocrine mechanisms.

We recapitulated the axis in which ECs act on peripheral sensory nerves via ET‐1/ETAR signaling, but we cannot deny the actions of SMCs and macrophages in the VP process. Although few inflammatory cells express ET‐1, with the increased infiltration of inflammatory cells, immune cell‐derived ET‐1 may also contribute to VP in the early stage, which should be further investigated. In fact, many clinical patients with PVD are at risk for cardiovascular events. Moreover, PVDs are often consistent across different phenotypes of atherosclerotic disease; of note, macrophages and SMCs play crucial roles in the process of arteriosclerosis.^[^ ^48^ ^]^ On the other hand, if PVP can be likened to atherosclerotic‐induced angina, this study has expanded our understanding of angina beyond an atherosclerosis‐focused perspective. These findings suggest that ECs could play a significant role in angina prior to the development of atherosclerosis.

Our data about a single optostimulation or chemostimulation leading to persistent effects suggest that the microenvironment of local ECs exhibits a positive feedback system; alternatively, it also reflects the powerful and important function of the EC system. Certainly, ECs play crucial roles in maintaining the organism's homeostasis by modulating cellular physiology, signaling pathways, and responses to various external and internal highly active molecules.^[^ ^8a^ ^]^ ECs can be activated by various stimuli including tumor necrosis factor (TNF)‐α, interleukin (IL)‐1β, thrombin, hypoxia (hypoxia inducible factor (HIF)‐1α activation), reactive oxygen species (ROS), mechanical stress (such as hypertension) and shear stress, which then lead to the release of ET‐1. ET‐1 acts on adjacent SMCs and immune cells through paracrine signaling (by binding to ETAR) or on ECs themselves through autocrine feedback (by binding to ETAR or ETBR). Activated SMCs and immune cells further stimulate ECs through paracrine signaling by releasing ET‐1 and binding to ETAR on the ECs' surface. ET‐1 binding to ETAR on ECs activates them, while binding to ETBR inhibits ECs.

There are several cascades involving ET‐1 paracrine and autocrine positive feedback loops: 1) NADPH oxidase → ROS production → nuclear factor (NF)‐κB activation → up‐regulation of ET‐1 expression; 2) Mitogen‐activated protein kinase (MAPK)/activator protein (AP)‐1 pathway → release of inflammatory factors (IL‐6 and chemokine (CC‐motif) ligand (CCL)2) → recruitment of immune cells → secretion of TNF‐α/IL‐1β → ET‐1 synthesis; 3) ETBR activation leading to endothelial nitric oxide synthase (eNOS) decoupling and oxidative stress; 4) Cross amplification of ET‐1 and other inflammatory pathways, including ① interaction with the coagulation system: ET‐1 promotes plasminogen activator inhibitor type (PAI)‐1 expression, worsening thrombosis. Thrombin released from the thrombus environment activates the endothelium through protease‐activated receptors (PARs), further stimulating ET‐1 production; ② synergy with vasoconstrictors: ET‐1 enhances blood vessel sensitivity to angiotensin II, forming a vasoconstriction‐ET‐1 feedback loop; ③interaction with vascular endothelial growth factor (VEGF): chronic ET‐1 signaling up‐regulates VEGF through HIF‐1α, increasing vascular permeability and activating macrophages to release TNF‐α, stimulating ET‐1 again; ④ ET‐1 also influences the immune response through mechanisms like transcription of proinflammatory cytokines, promoting chemotaxis. This can create a positive feedback system, as these cytokines also stimulate ET‐1 synthesis and release.^[^ ^8^, ^25^, ^47^, ^49^ ^]^ Additionally, it is worth noting that other pathways involving different molecules can also activate ECs and lead to ET‐1 release, creating additional pathways for positive feedback loops.^[^ ^50^ ^]^ Which positive feedback loops associated with PVP need to be clarified in further study, and how can they be activated? On the other hand, central maladaptation (Figure 1f) may contribute to the persistence of VP.

## 4 Conclusion

VP, a type of pain commonly experienced in patients with vascular dysfunction that has been understudied, was characterized at the phenotypic and mechanistic levels in mice. These findings revealed an axis by which ECs, but not macrophages or SMCs, mediated by ET‐1, activate primary sensory neurons by neural ETAR signaling, leading to VP, and this hypothesis was validated in clinical patients. We believe that targeting this axis and ETAR signaling represents therapeutic strategies for VP. Future research will aim to focus on the neural downstream of ETAR signaling involved in broad VP processes and vascular diseases, which may uncover a novel therapeutic strategy.

## 5 Experimental Section

### Animal Studies and Ethics

All experiments were carried out according to the relevant ethical guidelines of the International Association for the Study of Pain and approved by the Committees of the Use of Laboratory Animals of Nantong University (Approval ID: S20210223‐004) and Shantou University Medical College (Approval ID: SUMC2019‐081). Additional detailed information is provided in the Supporting Information.

### Human Study and Ethics Statement

This randomized, double‐blinded, and placebo‐controlled study was approved by the Ethical Committee of the Affiliated Hospital of Xuzhou Medical University (XYFY2017‐KL028‐01) and registered at clinicaltrials.gov (NCT03229694). Each participant provided written informed consent before entering the trial. Additional detailed information is provided in the Supporting Information.

Detailed methods are provided in the Supporting Information.

## Conflict of Interest

The authors declare no conflict of interest.

## Author Contributions

Z.J.J., D.M., S.L., and P.B.J. contributed equally to this work. X.J.L. and Y.G.S. conceived and secured funding. X.J.L. and T.L. wrote the manuscript. Z.J.J., S. L., D.M., P.B.J., B.Wu, Y.X.M., J.Y.G., Q.Y.L., H.H.C., F.M.Z., B.W., Y.Y.Z., L.Q., Z.Y.Z., Y.S.O., S.Y.S., L.L., Y.F., C.J., Y.C., and B.W. performed experiments and analyzed data. Z.S. provided reagents and transgenic mice. P.H.L., F.F.G., J.W., and Z.L. provided expertise and feedback.

## Supporting information



Supporting Information

## Data Availability

Research data are not shared.
