# Supplementary material for: Neuronal Endothelin a Receptor Mediates Experimental and Clinical Vascular Pain through an Endothelial‐Neural Axis
Source: Adv Sci (Weinh). 2025 Aug 14;12(42):e12375. doi: 10.1002/advs.202512375 (PMC12622552; doi:10.1002/advs.202512375)
Supplement: Supplementary file 1 — Supporting Information [file ADVS-12-e12375-s001.docx]

Supplementary Materials for

Neuronal Endothelin A Receptor Mediates Experimental and Clinical Vascular Pain through an Endothelial-Neural Axis

Zuo-Jie Jiang, Di Mu, Su Liu, Peng-Bo Jing, Bin Wu, Xiao-Mei Yang, Jia-Yi Ge, Qing-Yi Li, Hao-Hao Chen, Feng-Ming Zhang, Bing Wang, Ying-Ying Zhang, Linnan Qian, Zi-Yi Zhu, Yu-Sen Ou, Shi-Yu Sun, Lin Luo, Yu Feng, Changyu Jiang, Zhuo Sun, Yong Chen, Hai-Li Pan, Bin Wang, Fen-Fei Gao, Jie Wu, Zhigang Lu, Tong Liu, Yan-Gang Sun, Xing-Jun Liu

Correspondence to: luzg@njucm.edu.cn (Z.G.L); tongliu@ntu.edu.cn (T.L.); yangang.sun@ion.ac.cn (Y.G.S); edvin201@163.com (X.J.L)

**This PDF file includes:**

Materials and Methods

References S1-S20

Figures S1-S25

Tables S1-S3

Materials and Methods

**Animals**

All experiments were carried out according to relevant ethical guidelines of the International Association for the Study of Pain and approved by the Committees of the Use of Laboratory Animals of Nantong University (Approval ID: S20210223-004) and Shantou University Medical College (Approval ID: SUMC2019-081). SNS-Cre (Na_v_1.8 promoter)^[1]^, ET-1-flox and ETAR-flox^[2]^ transgenic mice were kindly provided by Prof. Rohini Kuner (University of Heidelberg) and Prof. Masashi Yanagisawa (University of Texas Southwestern Medical Center), respectively. Tek-Cre mice^[3]^ (strains 004128 and 008863) on the C57BL/6j background and Ai32 (channelrhodopsin-2 (ChR2)-flox) mice (strain 012569) and advillin-Cre (strain 032536)^[4]^ on the 129 background were purchased from Jackson Laboratory (Bar Harbor, ME, USA). ROSA26-CAG-flex-hM3Dq-mCherry-flox knock-in mice (C57BL/6j background) were generated by Beijing Biocytogen Accompany (Beijing, China). Cdh5-Cre/ERT2 strain^[5]^ on the C57BL/6jGpt background was kindly provided by Westlake University (Hangzhou, Zhejiang, China). Male transgenic mice were crossed with adult female wild-type (WT) C57BL/6J mice and maintained on the C57BL/6j background. Mice (2-5/cage) were housed under standard conditions (21-24°C, 60% humidity, 12:12-h light/dark cycle) and provided with free access to water and food.

All animals were kept in a sound-attenuated, isolated holding facility in the laboratory one week prior to surgery, after surgery, and throughout the duration of the behavioral assays to minimize stress. No statistical methods were used to predetermine the sample size. Animals were randomly assigned to treatment groups. Male and female mice (8 weeks old) were employed in most experiments, without considering female estrous cycle, and the data from both sexes were combined.

**Genotyping**

The animals were genotyped by PCR using ear punch tissues. All mouse lines maintained on a WT (C57BL/6j) background. Littermates of the same sex were randomly assigned to either the experimental or control group. The primer sequences and pairs used for each of the alleles for genotyping in this study are listed in Supplementary Table 1.

**Reagents**

Paraformaldehyde, picric acid, trypsin type I, collagenase type 1A, DNase I, pentobarbital sodium and tamoxifen (T5648), corn oil (C8267) and DAPI (D9542) were purchased from Sigma-Aldrich (St. Louis., MO, USA). Dulbecco's modified Eagle’s medium (DMEM) was provided by Gibco-Invitrogen (Carlsbad, CA, USA). Fetal bovine serum and 1,1'-dioctadecyl-3,3,3',3'-tetramethylindocarbocyanine perchlorate (DiI) were purchased from Thermo Fisher Scientific Inc. (Rockford, IL, USA). BQ-123 (5 nmol/5 μl, i.t.; 10~20 nmol/100~200 μl, intraperitoneal injection (i.p.); 1188/500μg) and BQ-788 (5 nmol/5 μl, i.t.; 10~20 nmol/100~200 μl, i.p.; 1500/1mg) were provided by Tocris Bioscience (Ellisville, Missouri, USA). Clozapine N-oxide (CNO, 34233-69-7) was purchased from Cayman Chemical Company (Ann Arbor, Michigan, USA). The recombinant adeno-associated viruses (AAVs) AAV2/9-EF1a-DIO-hM3D(Gq)-EGFP-WPRE and AAV2/9-EF1a-DIO-mChery-WPRE were purchased from OBiO Technology Corp., Ltd. (Shanghai, China). Tracleer (lot IP069A0101) was provided by Patheon Inc. (Mississauga, Ontario, Canada). RNAscope Multiplex Fluorescent Reagent Kit v2 (lot 323100), RNAscope™ Probe-Mm-Edn1 (lot 435221) and RNAscope™ Probe- Mm-Pecam1-C2 (lot 316721-C2) were purchased form Advanced Cell Diagnostics (Newark, CA, USA).

**Great Saphenous Vein and Saphenous Artery Ligation**

To perform great saphenous vein and saphenous artery ligation, mice were placed in the supine position after being anesthetized with 1% pentobarbital sodium (100 mg/kg, i.p.) on a temperature-controlled blanket to maintain a body temperature of 37 °C. After the inner thigh of the left hind limb was shaved, the superficial great saphenous vein and saphenous artery were able to be clearly seen through the skin. After routine disinfection, an approximately 0.5 cm longitudinal incision was made with ophthalmologic scissors along the inner side of the blood vessel about 0.5 cm and half of the tibia. The great saphenous vein (inner side) and its accompanying saphenous artery and saphenous nerve (outer side) were observed under an operating microscope. The tip of a tweezers was used to carefully puncture the mesangium between the vessels and nerve to separate the artery and vein, and then the artery and vein were completely ligated with a needle and 8-0 silk thread. After the lateral skin was lifted with tweezers to cover the blood vessels, the skin was conventionally stitched. In the sham control mice, thread was passed around the vessels and left in place, but the blood vessels were not ligated. The animals were allowed to recover on a heated blanket and then housed normally.

**Hindlimb Ischemia (HLI) Models**

To perform HLI model,^[6]^ mice were subjected to unilateral femoral artery and vein ligation. In brief, mice were placed in the supine position after being anesthetized with 1% pentobarbital sodium (100 mg/kg, i.p.) on a temperature-controlled blanket to maintain their body temperature. After the inner thigh of the left hind limb was shaved and subjected to routine disinfection, an approximately 0.5 cm incision was made on the upper hind limb, just distal to the peritoneal ridge, perpendicular to the limb with ophthalmologic scissors. The inguinal adipose tissue was gently separated using forceps to expose the femoral artery and vein. Using ring forceps, the femoral artery and vein were carefully separated and allowed two sutures to be passed underneath without damaging the internal iliac and epigastric arteries. These sutures (8-0 silk thread) were then used to create two ligatures (∼2 mm apart) of the femoral artery and vein. After the lateral skin was lifted with tweezers to cover the blood vessels, the skin was conventionally stitched. In the sham control mice, the incision was made, and 8-0 silk thread was passed around the vessel and left in place, with no ligatures being tied. The animals were allowed to recover on a heated blanket and then housed normally.

**Von Frey Tests**

To investigate mechanical hyperalgesia, von Frey tests were carried out as described in previous reports^[7]^. For mechanical pain measurement, the paw withdrawal threshold of mice in response to von Frey filament stimulation was determined. The mice were placed in boxes on an elevated metal mesh floor and allowed to habituate for at least 30 min. Then, their hind paws were stimulated with a series of von Frey hairs with logarithmically increasing stiffness (0.16, 0.40, 0.60, 1.00, and 2.00 g; Stoelting, Wood Dale, IL, USA). The hairs were applied perpendicular to the plantar surface of the paw, and the 50% paw withdrawal threshold was determined using Dixon’s up-down method. Mechanical allodynia was assessed by measuring the paw withdrawal frequency in response to repeated stimulation (10-20 times, >5 s between each stimulation) using subthreshold 0.16, 0.40 or 0.60 g von Frey filaments.

**Hargreaves Test**^[7]^

To test heat sensitivity, the mice were first habituated to a testing environment daily for one week. Then the mice were placed in plastic boxes, and hind paw withdrawal latency was measured using a Hargreaves radiating heat apparatus (IITC Life Science Inc., Woodland Hills, CA, USA) at the indicated time points following vessel ligation. After 30 min of acclimation, the paw withdrawal latency in response to radiant heat was calculated as the average of three or four measurements per paw over a 5-min testing period as a measure of thermal pain. The radiant heat intensity was adjusted so that the basal paw withdrawal latency between 10-15 s, and a cutoff of 20 s was used to avoid tissue damage.

**Acetone Test**^[8]^

To test cold pain, mice were habituated to a testing environment daily for two days before the behavioral test. In brief, the mice were placed in isolated chambers on an elevated metal mesh floor, and a drop of acetone (20 µl) was applied on the plantar surface of the hind paws with a 30-G syringe. Then, the number of hind paw flicks and licks within 5 min was counted. The cold pain score was calculated as the average of three measurements per paw over a 5-min testing period.

**Hot and Cold Plate Tests**^[7b]^

To confirm the heat hyperalgesia and cold allodynia results, hot and cold plate tests were performed with a hot/cold plate (IITC Life Science Inc.). Briefly, the mice were individually placed on a plate for at least 5 min per day on 3 consecutive days before the behavioral tests for habituation. Then, the cold plate test was performed at 0°C with a cutoff of 120 s, and the hot plate test was performed at 50°C or 52°C with a cutoff of 60 s. The paw withdrawal latency was calculated as the mean value of 3 measurements with an interval of at least 5 min.

**Tail Immersion**

Mice were placed in a mouse restraint and handled by experimenters for habituating the tail immersion procedure and habituated to the testing environment daily for two days before the behavioral test. The standard protocol involved three training days and a single testing day. On the training days, approximately half of the tail of each mouse was immersed in a hot water bath maintained at 52.0 ± 0.1°C to train the mouse to flick its tail out of the water bath. Each mouse was trained in 3 trials per day with a 10-minute quineibal interval for 3 days. The latency to mouse tail withdrawal from the hot water was recorded with a stopwatch, and a maximum latency of 20 s was used to avoid tissue injury. The formal experiment began on the fourth day. The tail withdrawal latency was calculated as the mean value of 3 measurements with an interval of at least 10 min.

**Rotarod Test**

The apparatus consisted of a rod with a diameter of 2.5 cm subdivided into five compartments. The bar rotated at speeds of 4-45 revolutions per minute (RPM). The ability of each animal to remain on the rotarod was assessed by measuring the time spent on the rods. The cutoff time for this test was 300 s.

The standard protocol involved three training days and a single testing day. On the training days, each mouse was subjected to 3 independent trials, during which the rod was accelerated (4-25, 4-35 and 4-45 revolutions per minute in 5 min each day), with an interval of at least 10 min. To prevent the mice from jumping deliberately, they were placed back on the rod if they fell. The experiment was performed on the fourth day. The time that each mouse spent on the rotating apparatus (4-45 RPM in 5 min) until it fell was recorded in seconds. The test was repeated 3 times with an interval of at least 10 min. The average of three trials was calculated as the duration (s).

**Open Field Test**

A square plastic box (50 × 50 × 45 cm) was used as an open box apparatus (ANY-Maze video tracking system; SDI, San Diego, CA, USA). Mice were placed individually in the center of the box, and their behavior was tracked with an overhead video camera connected to a laptop computer for 5 min. Then, the total distance traveled, the mean speed, the maximum speed and the number of the line crossings in the box were recorded. The recorded data were used as measures of total locomotor activity during the 5-min period.

**Laser Speckle Contrast Perfusion Imaging**

Immediately before surgical ligation, optostimulation or CNO injection and at the indicated time after surgery, optostimulation or CNO injection, the blood perfusion in the hindlimb paws was measured using a laser speckle contrast perfusion imager (RFLSI ZW Laser Speckle Imaging System, RWD Life Science Co. Ltd, Shenzhen, China; SIM BFI HR Pro, SIM Opto-Technology Co., Ltd, Wuhan, China). Mice were anesthetized 1% pentobarbital sodium (100 mg/kg, i.p.) on a heated blanket at 37°C while imaging was performed at the aforementioned specified times. To avoid data variations that may be caused by ambient light, temperature and the state of anesthesia, the perfusion in the ipsilateral hindpaw was normalized to the contralateral hindpaw and expressed as percent recovery.

**A****cute Nociceptive Behaviors**

The mice were handled by experimenters for habituation to the plantar injection procedure and habituated to the testing environment daily for two days before the behavioral tests. Then, chemogenetically-manipulated mice were administered intraplantar injections of 20 ng, 30 ng or 10 μg of CNO (in 10 μl saline) to induce acute pain behaviors after being individually placed in a plastic chamber (10 × 10 × 15 cm) with a mirrored side on an elevated metal mesh floor. Spontaneous pain behaviors were recorded with a video camera for 45 min, and the total response time of spontaneous pain-related behaviors (including paw biting, paw licking, paw flinching and jumping) was measured in seconds by a blinded observer using a stopwatch.

**Intrathecal Injection**^[7b, 9]^

After the animals were anesthetized with isoflurane (2%), the dorsal surface of each animal was shaved to expose the injection site three days prior. The site of subarachnoid puncture was determined by palpation of the iliac bone tuberosities, the spinous process of the last lumbar vertebra, and the area below the lumbosacral space. The lumbar (L)5-6 intervertebral spaces were identified by sliding the index finger along the midline in the rostral direction. After the animals were anesthetized, a sterile 30-G needle was advanced to the midline of the intervertebral space, with the bevel of the needle facing rostrally. When the needle tip was inserted into the intervertebral space at a depth of 2-3 mm, the precise subarachnoid positioning of the needle tip was verified by a brisk tail-flick. Then, the reagent (5 μl) was injected into the subarachnoid space in the cauda equine region. The needle was left in place for 5 s before being withdrawn to avoid reflux of the injected drug. To block the neural ETAR or ETBR, 5 nmol of BQ-123 or BQ-788 in 5 μl of saline were intrathecally injected; to block the neural TRPA1, TRPV1, ERK or CGRP receptor signaling, 5 μg/5 μl of HC-030031, 2 nmol/5 μl of AMG9810, U0126 (100 μM × 5 μl) or 5 μg/5 μl of BMS-927711 were used. Additionally, a vehicle control using 5 μl of saline was included. To neutralize CGRP in the spinal cord, a mouse monoclonal CGRP antibody (4 μg/5 μl was intrathecally injected, and a mouse IgG2A (4 μg/5 μl) was intrathecally injected as the control.

**Intraplantar Injection**

This test was performed as previously described.^[7b]^ Mice were handled and habituated for at least three days to avoid stress and then received an intradermal injection with a 26-G needle. After 15 min, the mice received an intraplantar injection of CNO (20 ng, 30 ng or 10 μg in 10 μl of saline/paw) at the same sites and were immediately returned to their chambers for video recording for 45 min. Finally, pain-related behaviors in the videos were quantified in a blinded manner by counting the time of pain-like responses.

For BQ-123 intraplantar injection, optogenetically manipulated mice received daily injection of BQ-123 (10 nmol in 10 μl of saline/paw) for three days and then were placed in chambers on a glass plate. The ECs in the hind paw skin of each Tek^cre^/ChR2^f/-^ mouse were activated with blue light (473 nm) at approximately 2.5 h after the last injection; and then, the paw withdrawal latency was measured at the indicated time points.

**Retrograde Tracing**

DiI retrograde neuronal labeling was performed as previously described.^[10]^ In short, 0.5 μl of 30 ng/μl DiI was injected into the mice in the area round the surgical sites using a 26-G needle. Seven days after DiI injection, the mice were deeply anesthetized (5% pentobarbital sodium, 100 mg/kg) and perfused with 4% paraformaldehyde and 0.02% picric acid in PBS. Then, the L1-6 dorsal root ganglia (DRGs) were dissected for histological observation.

**Immunohistochemical Staining and Imaging Analysis**

Immunofluorescence was performed as previously described.^[7b, 9, 11]^ Deeply anesthetized mice (2% pentobarbital sodium, 100 mg/kg) were perfused with 4% paraformaldehyde and 0.02% picric acid in PBS. The L4-5 DRGs, spinal cord (SC) and hind limbs (or the sciatic nerves and the saphenous nerves) were isolated and fixed in 4% paraformaldehyde for 1.5 h (DRGs) or overnight (SC, nerves and lower limbs), respectively. DRG sections (7 μm), SC sections (14 μm), the sections of ligated-vessel around tissues (10 μm) and the sciatic nerves and the saphenous nerves (7 μm) were prepared using a Leica CM1950 cryostat (Leica Camera AG, Wetzlar, Germany) or a cryostat (FS800, RWD Life Science Co., Ltd, Shenzhen, Guangdong, China). For ETAR and ET-1 staining, antigen retrieval was performed in citric acid buffer, and the sections were then blocked at room temperature. For the other immunostaining experiments, antigen retrieval was not necessary. The sections were incubated overnight at 4°C with a mouse primary monoclonal antibody against ETAR (1:800; Abmart Medicine Technology (Shanghai) Compony, Shanghai, China) combined with a guinea pig antibody against NeuN (1:2,000; Millipore, Billerica, MA, USA), or with a goat anti-ionized calcium-binding adapter molecule 1 (IBA-1) antibody (1:300; Abcam) combined with a mouse anti-glial fibrillary acidic protein (GFAP) antibody (1:300; Cell Signaling Technology, Danvers, MA, USA) or a rat anti-CD68 antibody (1:1,000; Bio-Rad, Hercules, CA, USA). Next, the sections were incubated with secondary antibodies. To validate the deletion of ETAR in Na_v_1.8^cre^/ETAR^f/f^ conditional knockout mice, the sections were incubated overnight at 4°C with a rabbit anti-ETAR antibody (1:1,000; GL Biochem Ltd, Shanghai, China), and incubated with secondary antibodies. After antigen retrieval, sections were stained with the mouse monoclonal antibody against Na_v_1.8 (1:50; NeuroMab, Davis, CA, USA).

For vessel staining, regions proximal (5 mm) and distal (3 mm) to the ligation site of the vessels were taken from the hind limbs for immunostaining. A rabbit primary antibody against ET-1 (1:2,000; Abcam) and a mouse primary antibody against CD31 (1:1,000; Abcam) or a goat primary antibody against α-Smooth muscle actin (α-SMA; 1:1,000; Novus Biologicals, Briarwood Avenue, CO, USA), combined with a rat anti-CD68 antibody (1:1000; Bio-Rad) were used. Tile scanning of the fluorescence images were performed with an LSM 800 laser-scanning confocal imaging system (Carl Zeiss, AG, Jena, Germany), and Image-Pro Plus 8.0 software (La Jolla, CA, USA) was used to analyze the fluorescence signals. For quantification of the immunostaining results, at least 3 sections (in very 4 sections) were selected for each animal, and at least 5 animals from each group were analyzed in a blinded manner. To determine the percentage of labeled neurons (labeled for ETAR, ET-1) in the DRGs, the number of positive neurons (3 times of the background signal) was divided by the total number of neurons indicated by NeuN immunostaining. For IBA-1, CD68 and GFAP immunostaining, the total density (arbitrary units) of labeled cells (per DRG or spinal dorsal horn (SDH)) was normalized to the entire area (in pixels) of the DRG or SDH. For the staining in the tissues surrounding ligated-vessels, the total number of CD68^+^ macrophages was determined by counting them in a single microscope field (1956 × 1956 pixels, 20× magnification); whereas for ET-1 immunostaining, the total density (arbitrary units) was normalized to the entire tissue area (in pixels) at 20× magnification. However, the representative images displayed in the formal figures of the manuscript were captured at a higher magnification (40×).

For ATF-3 staining, mice were perfused with 4% paraformaldehyde and 0.02% picric acid in PBS on day 7 after great saphenous vein and saphenous artery ligation, the two vessels with 1/3 the accompanying nerve ligation, the two vessels with the entire accompanying nerve ligation, or the sciatic nerve ligation, respectively. The L2-6 DRGs were isolated and fixed in 4% paraformaldehyde for 1.5 h. DRG sections (10 μm) were prepared using a cryostat (FS800, RWD Life Science Co., Ltd,). A rabbit polyclonal antibody against ATF-3 (1:500; Santa Cruz Biotechnology, Inc, Dallas, Texas, USA), combined with a guinea pig antibody against NeuN (1:2,000; Millipore, Billerica, MA, USA) were employed for the section incubation. Finally, the fluorescence images were captured with a MSHOT MIX60-FL microscope (Guangzhou Micro-shot Technology Co., Ltd, Guangzhou, Guangdong, China).

**DRG Neuron Culture**

DRG neurons were harvested and cultured as previously described.^[9, 12]^ Briefly, mice were deeply anesthetized with isoflurane and decapitated, and then the L4-5 DRGs were quickly excised and collected in ice-cold Dulbecco’s Modified Eagle Medium (DMEM, Gibco). The DRGs were digested with enzyme solution (3.0 U/ml dispase and 0.6 U/ml collagenase type D dissolved in D-Hanks without Ca^2+^ or Mg^2+^; Gibco) at 37°C for 40 min, and then mechanically dissociated with Pasteur pipettes. DRG neurons were suspended in DH10 medium containing 90% DMEM/F-12 (Gibco), 10% fetal bovine serum (Thermo Fisher Scientific) and 1% penicillin-streptomycin (100 U/ml penicillin and 100 µg/ml streptomycin; Solarbio) and filtered through a 100 μm cell strainer (BD, Franklin Lakes, NJ, USA). After centrifugation at 1,200 rpm for 5 min, the DRG neurons were resuspended in warm DH10 medium with NGF (25 ng/ml; Merck Millipore) and plated on poly-D-lysine and laminin (Sigma)-coated glass coverslips. The plated DRG neurons were cultured in an incubator (95% O_2_ and 5% CO_2_) at 37°C and used for calcium imaging and patch-clamp recordings within 24 h.

For immunostaining of cultured neurons, the DRGs of adult mice were dissociated, digested with an enzyme mixture of collagenase I, trypsin and DNase I. After centrifugation with 15% Percoll to remove nonneuronal cells, DRG neurons were obtained and cultured in neurobasal medium containing 2% B27 supplement, 2 mM L-glutamine (Invitrogen), and 10 μM 5-fluoro-2’-deoxyuridine (Sigma) to inhibit the proliferation of nonneuronal cells. DRG neurons plated on coverslips were treated with ET-1 (100 ng/ml) after 24 h or 48 h and fixed in 4% paraformaldehyde for 15 min at room temperature. Then, the neurons were incubated with anti-CGRP primary antibody (1:1,000, ImmunoStar, Hudson, WI, USA) overnight at 4°C followed by secondary antibodies conjugated to fluorescent dye (1:1,000; Jackson ImmunoResearch Laboratories, West Grove, PA, USA) for 45 min at 37°C. Fluorescent images were acquired with a Leica TCS SP8 confocal microscope.

**In Situ Hybridization**

After perfusion and tissue fixation, mouse skins were sectioned into frozen slices at a thickness of 14 μm. Sections underwent dehydration through a graded ethanol series, treatment with 3% hydrogen peroxide, target retrieval solution, and protease digestion. RNAscope was performed following the manufacturer’s instructions using the RNAscope™ Multiplex Fluorescent Reagent Kit v2. The probes used in this study were RNAscope™ Probe-Mm-Edn1 (Gene ID 13614) and RNAscope™ Probe- Mm-Pecam1-C2 (Gene ID 18613). Signal amplification was performed using the OPAL 520 Reagent Pack (FP1487001KT) and OPAL 570 Reagent Pack (FP1488001KT). DAPI (D9542) staining was performed at a 1:5000 dilution for 5min. Imaging was conducted using captured with a Leica TCS SP8 laser scanning confocal imaging system, then images were analyzed by Image-Pro Plus 8.0 software. For ET-1 mRNA quantification, the total fluorescence density (arbitrary units) was normalized to the entire tissue area (in pixels) at 40× magnification; To count DAPI staining cells, the total number of DAPI^+^ nucleus was determined by counting them in a single microscope field (1024 × 1024 pixels, 40 × magnification).

**Measurement of ET-1 Concentration in Mouse Serum**

Under isoflurane anesthesia, heart blood was extracted from the hearts of the experimental mice with a syringe after thoracotomy. Alternatively, blood was taken locally from vessel ligation sites or hindpaws with a glass straw after skin incision. The collected whole blood was left at room temperature for 2 h, then centrifuged at 3,000g for 10 min. The upper yellow serum sample was transferred to an EP tube, frozen in liquid nitrogen, and stored at -80^o^C. The concentration of serum ET-1 was detected following the instructions of the ELISA kit (Lot: MM-0561M1; Jiangsu Meimian industrial Co., Ltd, Yancheng, China).

**Calcium Imaging**

Four weeks after adult male mice (8 weeks old) underwent vessel ligation, the L4-5 DRGs were quickly excised, and DRG neurons were cultured for overnight. The cultured DRG neurons were loaded with Fura-2 (1:1,000; Thermo Fisher) and F127 (0.01% w/v, Invitrogen) at 37°C in a CO_2_ incubator maintaining 5% CO_2_. After loading for 30 min, the DRG neurons were washed twice with calcium imaging buffer (in mM: 137 NaCl, 5.4 KCl, 1.2 MgCl_2_, 1.0 CaCl_2_, 1.0 NaH_2_PO_4_, 10 glucose, 20 HEPES; adjusted to Ph 7.3-7.4 with NaOH). Glass coverslips containing DRG neurons were placed in a chamber and perfused with calcium imaging buffer. Ratiometric calcium imaging was performed using a high-resolution CCD camera (Excelitas PCO GmbH, Donaupark, Kelheim, Germany) equipped with an inverted microscope (Nikon Instruments Inc., Tokyo, Japan). The calcium signals at an excitation wavelength of 340 and 380 nm supplied by an alternating light source (PTI, New Jersy, USA) were recorded to measure the changes in intracellular calcium concentration. Neurons in the imaging chamber, equipped with a bespoke four-channel perfusion valve control system, were first stabilized in calcium imaging buffer. Subsequent to baseline stabilization, then infused with 40 nM of ET-1 for 1 minutes, followed by a 30-s perfusion with 56 nM of KCl, and finally re-equilibrated with CIB to return to baseline conditions.

**Whole-Cell Patch-Clamp Recording**^[7]^

Four weeks after adult male mice (8 weeks old) underwent vessel ligation, the L4-5 DRGs were quickly excised, and DRG neurons were cultured for 12-20 h. Whole-cell current-clamp recordings were performed at room temperature using a MultiClamp 700B amplifier and Pclamp10 software (Molecular Devices Corporation, Sunnyvale, CA, USA) driven by a personal computer in conjunction with an A/D and D/A board (DigiData 1550B series interface, Molecular Devices Corporation). Patch pipettes were pulled using a Sutter P-1000 puller (Sutter Instrument, Novato, CA, USA) and had a resistance of 5-8 MΩ after being filled with internal solution. The internal solution contained (in Mm) 30 KCl, 110 potassium gluconate, 0.5 EGTA, 5 HEPES, and 3 Mg-ATP (adjusted to Ph 7.30 with KOH and an osmolarity of 300-310 mOsm with sucrose). The external solution contained (in mM) 140 NaCl, 3 KCl, 2 MgCl_2_, 2 CaCl_2_, 10 mM HEPES, (adjusted to Ph 7.30 with NaOH and an osmolarity of 310-320 mOsm with sucrose). The resting membrane potential of each DRG neuron with a diameter of ~25 μm was recorded in the current-clamp mode. Action potentials (Aps) were evoked by two stimulus protocols, a ramp protocol and step-by-step protocol (stimulus range from 0 to 100 Pa or 200 Pa and 300 Pa, 1,000 ms in duration for the ramp protocol; stimulus range from ~20 to 100 Pa, 1000 ms in duration, 20 Pa or 50 Pa increment for step-by-step protocol). The short current steps (50-300 mV, 2 ms in duration) were used to trigger single action potential that was used to measure the shape properties of Aps. A series of ramp currents were also used to study the excitability of neurons. Signals were low-pass filtered at 5 kHz, sampled at 20 kHz and analyzed offline. Forty nM of ET-1 in the external solution was tested.

**Paw Edema Measurement**

To assess the paw edema, paw thickness was measured as previously described.^[4]^ The average of three paw thickness measurements was recorded using a microcaliper at the indicated time points in a blind manner.

**Optogenetic Manipulation**

Tek-Cre (Tie2-Cre, EC-specific expression, strains 004128 or 008863) mice were crossed with Ai32 (Ai32(RCL-ChR2(H134R)/EYFP)-flox mice,^[4, 13]^ and conditional knock-in Tek^cre^/ChR2^+/-^ mice and littermate control Tek^-^/ChR2^+/-^ mice were used. As for the radiating thermal pain (Hargreaves) test, the experimental mice were placed on a glass plate. After habituation for three days, the ECs in the hind paw skin of the mice were activated with blue light (473 nm; BL473T3-100FC, Shanghai Laser & Optics Century Co., Ltd., Shanghai, China). The laser intensity was measured with digital Sanwa LP-1 laser power meter (SP10; Sanwa Electric Instrument Co., Ltd., Tokyo, Japan). The optimal conditions within a terminal intensity of 40 mW/mm^2^-1.0 mW/mm^2^ for testing Tek^cre^/ChR2^+/-^ mice, a pulse of 1-10 Hz and an illumination duration of 2-20 ms were determined. The withdrawal latency of the paw was measured using a stopwatch (cutoff of 30 s) after blue light stimulation (40 mW/mm^2^, 10 Hz and 5 ms); spontaneous pain behaviors were videotaped and further analyzed after blue light stimulation (1 mW/mm^2^, 10 Hz and 5 ms). To determine whether chronic vascular pain was established, the mechanical pain thresholds of the mice were measured at the indicated time points after optogenetic stimulation with blue light (1.0 mW/mm^2^, 10 Hz and 5 ms) three consecutive times for three cycles within 20 min (nine stimulations in total). Yellow light (593 nm; YL589T6-100FC, Shanghai Laser & Optics Century Co., Ltd.) was applied to the plantar hind paws of the mice as a control.

**Chemogenetic Manipulation**^[14]^

Tek-Cre (Tie2-Cre, strains 004128 or 008863) mice were crossed with ROSA26-CAG-flex-Hm3Dq-EYFP-flox mice to generate Tek^cre^/hM3Dq^+/-^ and littermate control Tek^-^/hM3Dq^+/-^ mice. After injection of CNO (30 ng for Tek^cre^/ChR2^+/-^ mice in 10 μl saline/paw), adult mice were immediately videotaped for 45 min. Induced spontaneous pain behaviors were observed and counted in a double-blind manner. Then mechanical stimuli (von Frey hairs) were used to determine whether chronic vascular pain was induced at the indicated time points.

To evaluate acute nociceptive behaviors induced by activated ECs after chemogenetic stimulation, adult Tek-Cre (strain 004128) mice were intraplantarly administered AAV(2/9)-EF1a-DIO-hM3D(Gq)-mCherry-WPRE or AAV(2/9)-EF1a-DIO-EGFP-WPRE (1.32 × 10^10^ viral genomes (V.G.)/5 μl). Three weeks after intraplantar injection, Tek^cre^/AAV-hM3Dq and Tek^cre^/AAV-EGFP mice were intraplantarly injected with CNO (30 μg in 10 μl saline/paw) and immediately videotaped for 45 min. Then, spontaneous pain behaviors were counted in a double-blind manner.

**Clinical Study**

This randomized, double-blinded and placebo-controlled study was approved by the Ethic Committee of the Affiliated Hospital of Xuzhou Medical University (XYFY2017-KL028-01) and registered at clinicaltrials.gov (NCT03229694). It was performed from September 2017 to March 2019 at the Affiliated Hospital of Xuzhou Medical University. Each participant provided written informed consent before entering the trial.

Eighty-nine patients scheduled for unilateral total knee arthroplasty (75 patients) or the connection of tibia or fibula fracture (14 patients) under general anesthesia were enrolled. The following inclusion criteria were employed: a. American Society of Anesthesiologists (ASA) class II-III, b. requirement of tourniquet inflation of the lower limbs during operation, c. an age of 18-75 years and d. agreement to voluntary provide signed informed consent. The following exclusion criteria were employed: a. no tourniquet applied during the surgery, b. emergency surgery, c. application of tourniquets within the last three months, d. bilateral total knee arthroplasty, e. tourniquet inflation less than 60 min or longer than 90 min, f. history of daily intake of analgesics, g. dysfunction of the liver or kidney, h. serious myocardial disease (coronary heart disease, heart failure, or severe arrhythmia), i. coagulation disorder, j. diabetes, k. history of drug or alcohol abuse, l. pneumonia, asthma or chronic obstructive pulmonary disease, m. hypotension before surgery (systolic pressure < 90 mmHg) and n. pregnancy or recent childbirth. For sample size calculation, we used a proportion of rescue analgesia of 36%, which was identified in our pilot experiments. Therefore, the use of 42 subjects per group would allow detection of a 25% reduction between groups at a significance level of 5% and a power of 80%. Anticipating a 10% dropout rate, we estimated that 45 patients were needed in each group.

A random sequence was generated by Stata 12.0. Each individual was randomly assigned to the Tracleer group (T group) or placebo control group (C group) at a 1:1 ratio. The researcher, patients and statistical analysts were blinded to the group assignments. Allocation concealment was achieved by placing a random sequence that was used to determine the group assignments in opaque, sealed envelopes that were opened 3 h before surgery. All patients fasted for 8 h before surgery. Patients in the T group received Tracleer (125 mg, tablet) orally two hours before surgery and six hours after surgery. Patients in the placebo control group received placebo at the same time points. Anesthesia was induced with midazolam (0.02 mg/kg), etomidate (0.3 mg/kg), fentanyl (2.0-4.0 μg/kg) and rocuronium (0.5 mg/kg). Anesthesia was maintained with propofol (3.0-6.0 mg/kg/h) and remifentanil (0.1-0.3 μg/kg/min). Muscle paralysis was maintained with rocuronium. The tidal volume was adjusted to keep the end-tidal CO_2_ concentration between 35 and 45 mmHg. Patient-controlled intravenous analgesia (PCIA) was maintained with sufentanil (2.0 μg/kg) and tropisetron (6.0 mg) in a total volume of 100 ml saline was used for postoperative analgesia. The PCIA background infusion rate was 1 ml/h, and each bolus volume was 0.5 ml, with a lockout interval of 10 min. If the VAS score was more than 5, the patient received intravenous injection of dezocine (5.0 mg). The interval between each injection was no less than 6 h.

The primary outcome was the change in VAS score at different time points after the operation. The secondary outcomes included the doses of propofol and remifentanil used during operation, the number of PCIA presses, the incidence of remedial analgesia, the concentration of ET-1 in plasma, and the incidence of side effects.

There was no substantial difference between the groups with regard to age, gender, height, weight, blood pressure, and the operation time and tourniquet time (Table S2).

**Measurement of ET-1 Levels in Plasma**

Venous blood samples (2.0 ml) were collected in plastic tubes containing EDTA and centrifuged at 3000 × g for 10 min, and plasma was collected and stored at -80^o^C. The plasma level of ET-1 was determined by using an ET-1 enzyme-linked immunosorbent assay (ELISA) kit (QET00B, R&D systems) according to the manufacturer’s instructions.

**Data Collection and Statistics**

All data were recorded in Microsoft Excel and further analyzed using GraphPad Prism 8 (La Jolla). All data are expressed as the mean ± standard error of the mean (SEM), and differences were considered statistically significant at a P-value of < 0.05 (Table S3). The reported animal or sample sizes were the number of independent values, and statistical analyses were performed using these independent values. For calcium imaging and electrophysiology data, each data point corresponded to a single neuron and recordings were made from at least 5 different mice; for immunofluorescence data, each data point corresponded to a single DRG neuron or SDH section and at least 5 different mice or rats were used. For all histological experiments, mice and rats were randomly selected; however, for the behavioral pain tests, to control unwanted variations in the pain threshold before and after modeling and ensure the comparability of pain thresholds at multiple time points before and after drug treatment, litter-matched and age-matched animals were assigned to experimental groups to generate biological replicates based on baselines and preadministration values (pain threshold), and the behavioral tests and data analyses were performed in a blinded manner. For all animal-related experiments, no outliers were excluded. All data sets were tested for normality and equal variance and then analyzed using Student’s unpaired *t*-test (for two groups), chi-squared test (for two constituent ratios), one-way repeated-measures ANOVA (for multiple groups), or two-way repeated-measures ANOVA (for time course analysis) followed by post hoc Sidak’s or Tukey’s multiple comparisons test using GraphPad Prism 8.0 (GraphPad Software Ltd., San Diego, CA, USA).

**Data Availability**

All relevant data and videos for this study can be made available by the corresponding author upon reasonable request.

**References**

[1] a) N. Agarwal, S. Offermanns, R. Kuner, Conditional gene deletion in primary nociceptive neurons of trigeminal ganglia and dorsal root ganglia, *Genesis* **2004**, *38* (3), 122, https://doi.org/10.1002/gene.20010; b) P. B. Jing, Y. W. Zhou, F. M. Zhang, J. Y. Ge, J. N. Wu, J. H. Xu, X. H. Cao, N. Chang, X. Zhou, L. Luo, X. J. Liu, Autistic-like behaviors and impaired chronic inflammatory pain in primary nociceptive neuron-specific deletion of Mecp2 or Fmr1 knockout male mice, *Behavioural Brain Research* **2025**, *486*, https://doi.org/ARTN 115570. 10.1016/j.bbr.2025.115570; c) X. J. Liu, Y. Zhang, T. Liu, Z. Z. Xu, C. K. Park, T. Berta, D. Jiang, R. R. Ji, Nociceptive neurons regulate innate and adaptive immunity and neuropathic pain through MyD88 adapter, *Cell Res* **2014**, *24* (11), 1374, https://doi.org/10.1038/cr.2014.106.

[2] a) R. M. Kedzierski, P. A. Grayburn, Y. Y. Kisanuki, C. S. Williams, R. E. Hammer, J. A. Richardson, M. D. Schneider, M. Yanagisawa, Cardiomyocyte-specific endothelin A receptor knockout mice have normal cardiac function and an unaltered hypertrophic response to angiotensin II and isoproterenol, *Mol Cell Biol* **2003**, *23* (22), 8226, https://doi.org/10.1128/Mcb.23.22.8226-8232.2003; b) B. Wang, J. Y. Ge, J. N. Wu, J. H. Xu, X. H. Cao, N. Chang, X. Zhou, P. B. Jing, X. J. Liu, Y. Wu, Endothelin A receptor in nociceptors is essential for persistent mechanical pain in a chronic pancreatitis of mouse model, *World J Gastroenterol* **2025**, *31* (23), 103848, https://doi.org/10.3748/wjg.v31.i23.103848.

[3] Y. Y. Kisanuki, R. E. Hammer, J. Miyazaki, S. C. Williams, J. A. Richardson, M. Yanagisawa, Tie2-Cre transgenic mice: a new model for endothelial cell-lineage analysis in vivo, *Dev Biol* **2001**, *230* (2), 230, https://doi.org/10.1006/dbio.2000.0106.

[4] H. H. Chen, M. Mohsin, J. Y. Ge, Y. T. Feng, J. G. Wang, Y. S. Ou, Z. J. Jiang, B. Y. Hu, X. J. Liu, Optogenetic Activation of Peripheral Somatosensory Neurons in Transgenic Mice as a Neuropathic Pain Model for Assessing the Therapeutic Efficacy of Analgesics, *ACS Pharmacol Transl Sci* **2024**, *7* (1), 236, https://doi.org/10.1021/acsptsci.3c00254.

[5] I. Sorensen, R. H. Adams, A. Gossler, DLL1-mediated Notch activation regulates endothelial identity in mouse fetal arteries, *Blood* **2009**, *113* (22), 5680, https://doi.org/10.1182/blood-2008-08-174508.

[6] M. J. Zhang, B. E. Sansbury, J. Hellmann, J. F. Baker, L. P. Guo, C. M. Parmer, J. C. Prenner, D. J. Conklin, A. Bhatnagar, M. A. Creager, M. Spite, Resolvin D2 Enhances Postischemic Revascularization While Resolving Inflammation, *Circulation* **2016**, *134* (9), 666, https://doi.org/10.1161/Circulationaha.116.021894.

[7] a) Z. Z. Xu, X. J. Liu, T. Berta, C. K. Park, N. Lu, C. N. Serhan, R. R. Ji, Neuroprotectin/Protectin D1 Protects against Neuropathic Pain in Mice after Nerve Trauma, *Annals of Neurology* **2013**, *74* (3), 490, https://doi.org/10.1002/ana.23928; b) Y. K. Huang, Y. G. Lu, X. Zhao, J. B. Zhang, F. M. Zhang, Y. Chen, L. B. Bi, J. H. Gu, Z. J. Jiang, X. M. Wu, Q. Y. Li, Y. L. Liu, J. X. Shen, X. J. Liu, Cytokine activin C ameliorates chronic neuropathic pain in peripheral nerve injury rodents by modulating the TRPV1 channel. *Brit J Pharmacol* **2020**, *177* (24), 5642, https://doi.org/10.1111/bph.15284.

[8] R. Y. Huang, L. Poree, K. Y. Ho, S. Y. Tsai, Y. C. Liu, P. H. Tan, Y. R. Wen, Behavioral Survey of Effects of Pulsed Radiofrequency on Neuropathic and Nociceptive Pain in Rats: Treatment Profile and Device Implantation, *Neuromodulation* **2020**, https://doi.org/10.1111/ner.13169.

[9] X. J. Liu, F. X. Zhang, H. Liu, K. C. Li, Y. J. Lu, Q. F. Wu, J. Y. Li, B. Wang, Q. Wang, L. B. Lin, Y. Q. Zhong, H. S. Xiao, L. Bao, X. Zhang, Activin C expressed in nociceptive afferent neurons is required for suppressing inflammatory pain, *Brain* **2012**, *135*, 391, https://doi.org/10.1093/brain/awr350.

[10] H. Baier, S. Rotter, S. Korsching, Connectional topography in the zebrafish olfactory system: random positions but regular spacing of sensory neurons projecting to an individual glomerulus, *Proc Natl Acad Sci U S A* **1994**, *91* (24), 11646, https://doi.org/10.1073/pnas.91.24.11646.

[11] X. J. Liu, T. Liu, G. Chen, B. Wang, X. L. Yu, C. Yin, R. R. Ji, TLR signaling adaptor protein MyD88 in primary sensory neurons contributes to persistent inflammatory and neuropathic pain and neuroinflammation, *Sci Rep-Uk* **2016**, *6*, https://doi.org/ARTN 28188

10.1038/srep28188.

[12] G. Hoeffel, G. Debroas, A. Roger, R. Rossignol, J. Gouilly, C. Laprie, L. Chasson, P. V. Barbon, A. Balsamo, A. Reynders, A. Moqrich, S. Ugolini, Sensory neuron-derived TAFA4 promotes macrophage tissue repair functions, *Nature* **2021**, *594* (7861), 94, https://doi.org/10.1038/s41586-021-03563-7.

[13] a) J. A. Cohen, T. N. Edwards, A. W. Liu, T. Hirai, M. R. Jones, J. N. Wu, Y. Li, S. Q. Zhang, J. Ho, B. M. Davis, K. M. Albers, D. H. Kaplan, Cutaneous TRPV1(+) Neurons Trigger Protective Innate Type 17 Anticipatory Immunity, *Cell* **2019**, *178* (4), 919, https://doi.org/10.1016/j.cell.2019.06.022; b) A. M. Cowie, F. Moehring, C. O'Hara, C. L. Stucky, Optogenetic Inhibition of CGRP alpha Sensory Neurons Reveals Their Distinct Roles in Neuropathic and Incisional Pain, *Journal of Neuroscience* **2018**, *38* (25), 5807, https://doi.org/10.1523/Jneurosci.3565-17.2018.

[14] a) D. Mu, J. Deng, K. F. Liu, Z. Y. Wu, Y. F. Shi, W. M. Guo, Q. Q. Mao, X. J. Liu, H. Li, Y. G. Sun, A central neural circuit for itch sensation, *Science* **2017**, *357* (6352), 695, https://doi.org/10.1126/science.aaf4918; b) A. Sorrentino, T. Michel, Redox a la carte: Novel chemogenetic models of heart failure, *Br J Pharmacol* **2020**, *177* (14), 3162, https://doi.org/10.1111/bph.15093.

Supplementary Figure 1


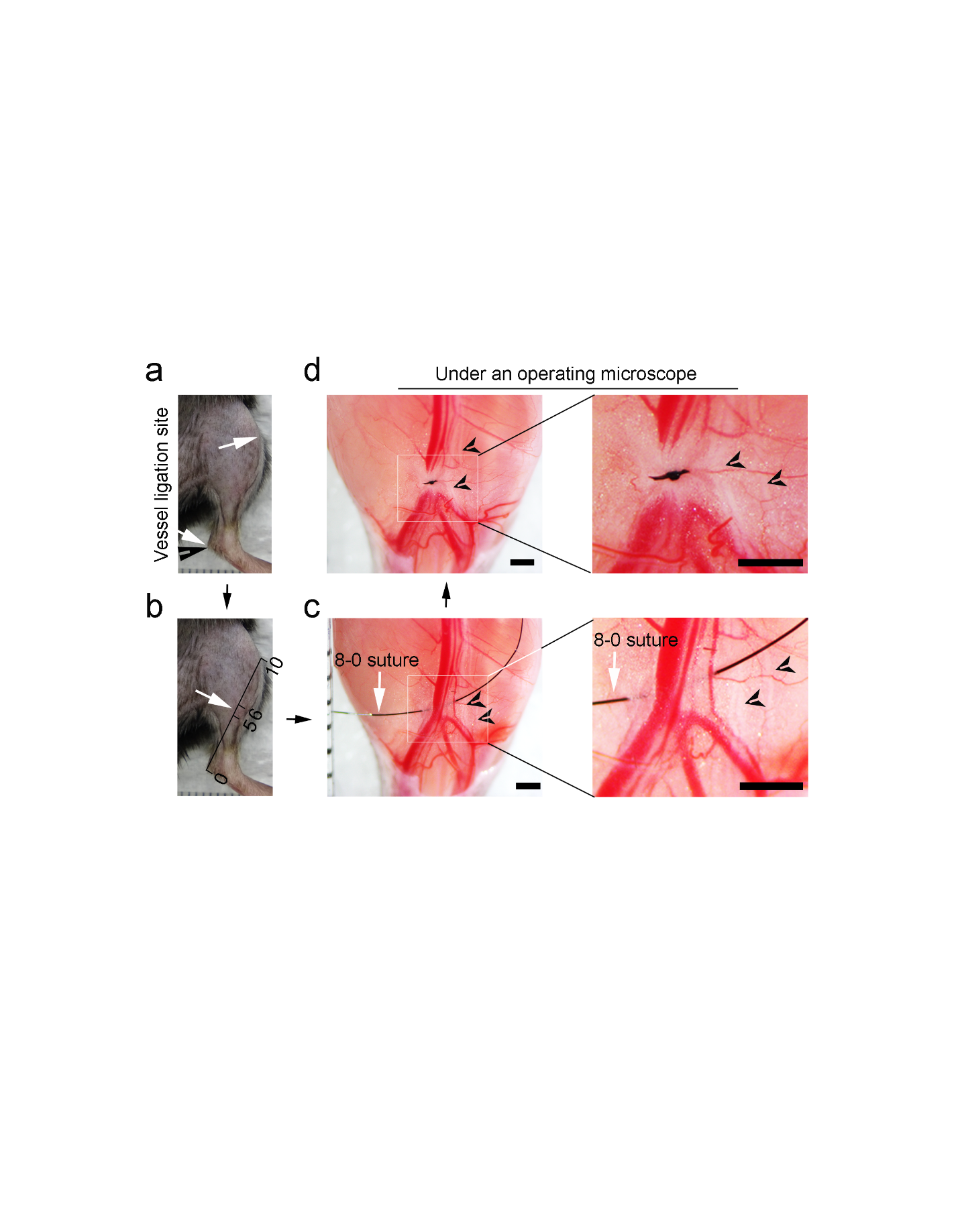


Figure. S1.

**Schematic and images of the site of vessel ligation of mice induced PVP. a, b** Schematic of the site of vessel ligation induced PVP. **c, d** Images showing a distance between the ligated vessels and accompaning nerves. Arrowheads indicate the intact acommpaning nerves. Scale bars, 1.0 mm.

Supplementary Figure 2


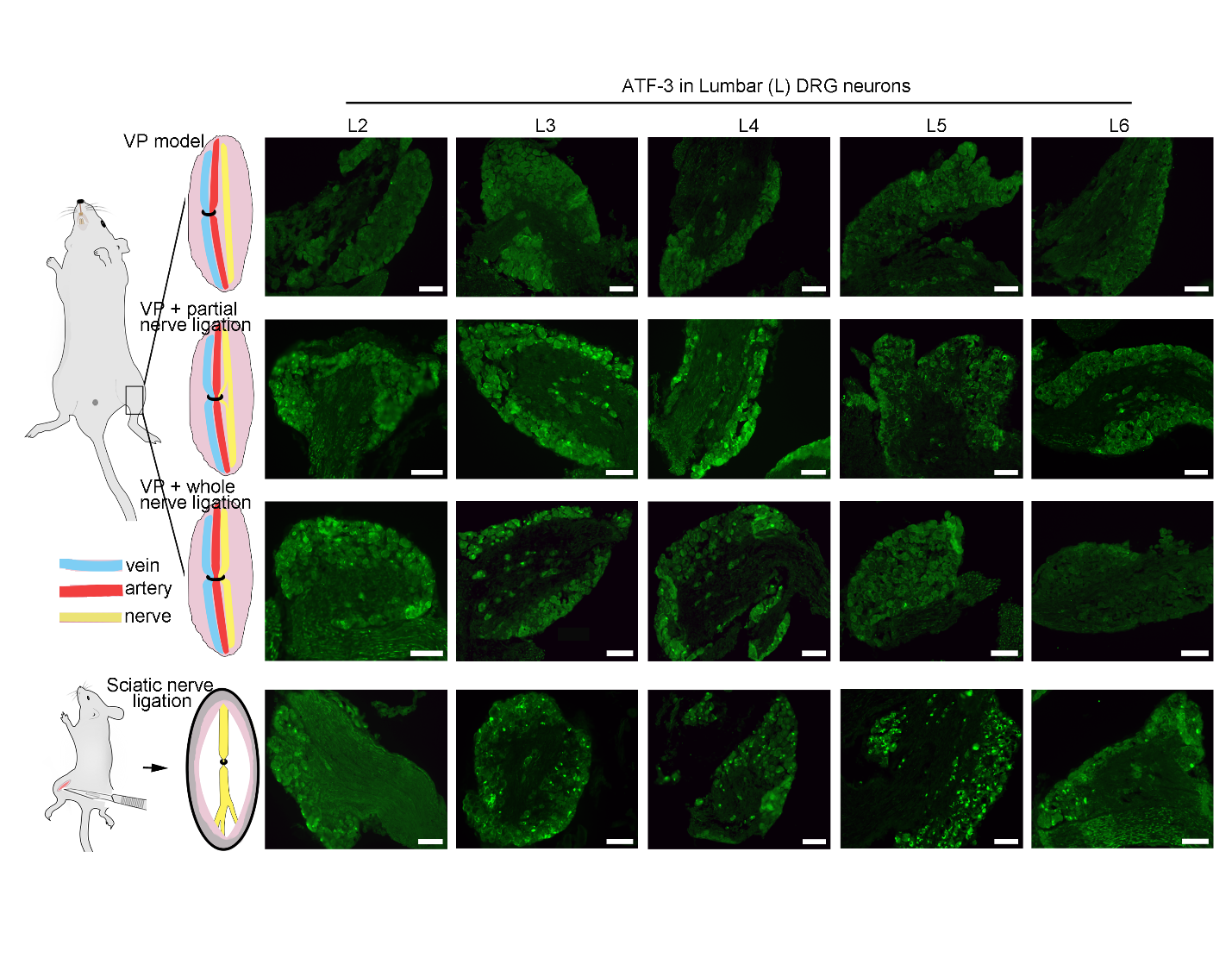


Figure. S2.

**ATF-3 expression on primary sensory neurons in various segments of lumbar ganglia following ligation of vessels, ligation of vessels and partial saphenous nerves, ligation vessels and entire saphenous nerves, or ligation of the sciatic nerve, respectively.** Scale bars, 100 μm. ATF-3, activating transcription factor-3; DRG, dorsal root ganglion; L, lumbar; VP, vascular pain.

Supplementary Figure 3


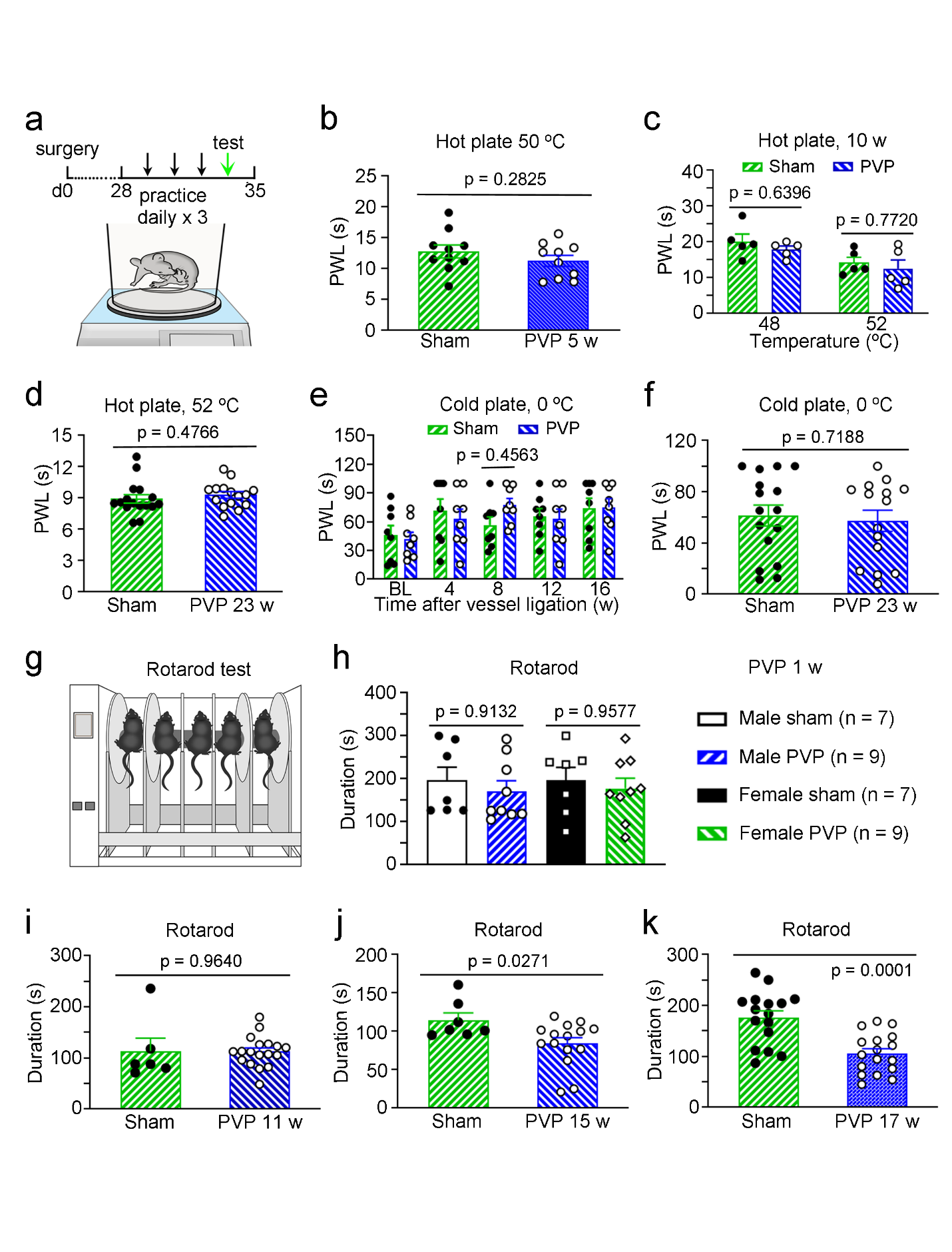


Figure. S3.

**Behavioral phenotypes of heat and cold pain and motor coordination in PVP mice. a** Schematic of the hot/cold plate test. **b**-**f** No heat hyperalgesia (**b**-**d**) or cold allodynia (**e**, **f**) was observed in the hot/cold plate test (**a**) in the early (**b**, **e**), middle (**c,** **e**) and late (**d**-**f**) phases in PVP mice. **g** Schematic of the accelerating rotarod test. **h**-**k** Impairment of motor function in an accelerating rotarod test in the late phase (**j,** **k**) but not in the early (**h**) and middle (**i**) phases in PVP mice. BL, baseline; Maxi., maximum; PVP, peripheral vascular pain; PWL, paw withdrawal latency. n = 10 (b), 5 (c), 15~16 (d), 8(e), 15~16 (f), 7~9 (h), 6~18 (i), 7~15 (j) and 16~17 (k) mice. The data are presented as the means ± SEM; statistical comparisons were conducted with unpaired two-tailed *t*-test (b, d, f, i-k), one-way ANOVA (h) or two-way ANOVA (c, e) with Sidak’s post hoc test.

Supplementary Figure 4


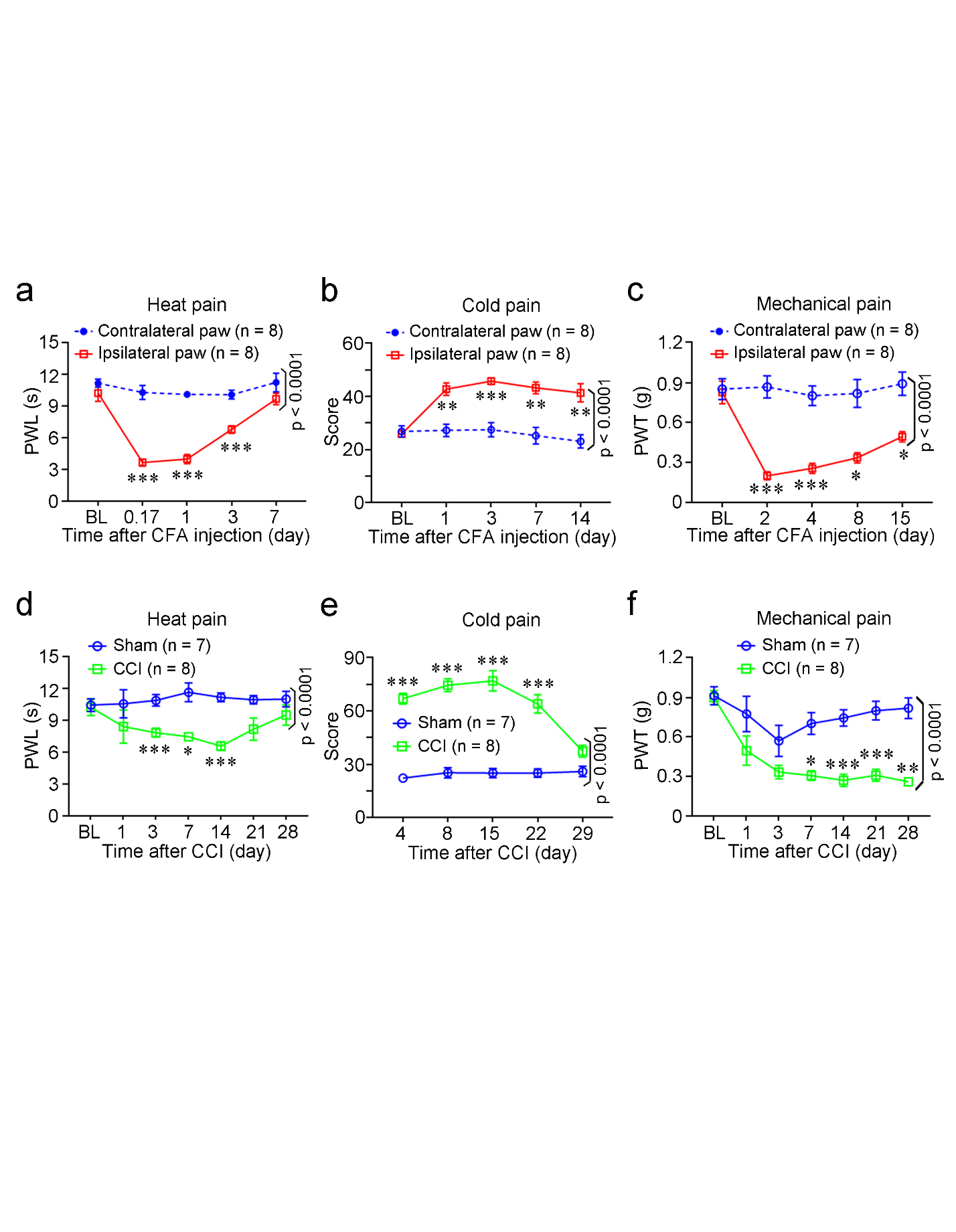


Figure. S4.

**Pain phenotype in** **chronic inflammatory pain and neuropathic pain in mice.** **a-c** The phenotype of heat (**a**), cold (**b**) and mechanical pain (**c**) in chronic inflammatory pain mice induced by CFA-intraplantar injection. **d-f** The phenotype of heat (**d**), cold (**e**) and mechanical pain (**f**) in chronic neuropathic pain mice induced by CCI. BL, baseline; CCI, chronic constriction injury; CFA, complete Freund’s adjuvant; PWL, paw withdrawal latency. n = 8 (a-c) and 7~8 (d-e) mice. The data are presented as the means ± SEM; *p < 0.05, **p < 0.01 and ***p < 0.001; statistical comparisons were conducted with two-way ANOVA with Sidak’s post hoc test.

Supplementary Figure 5


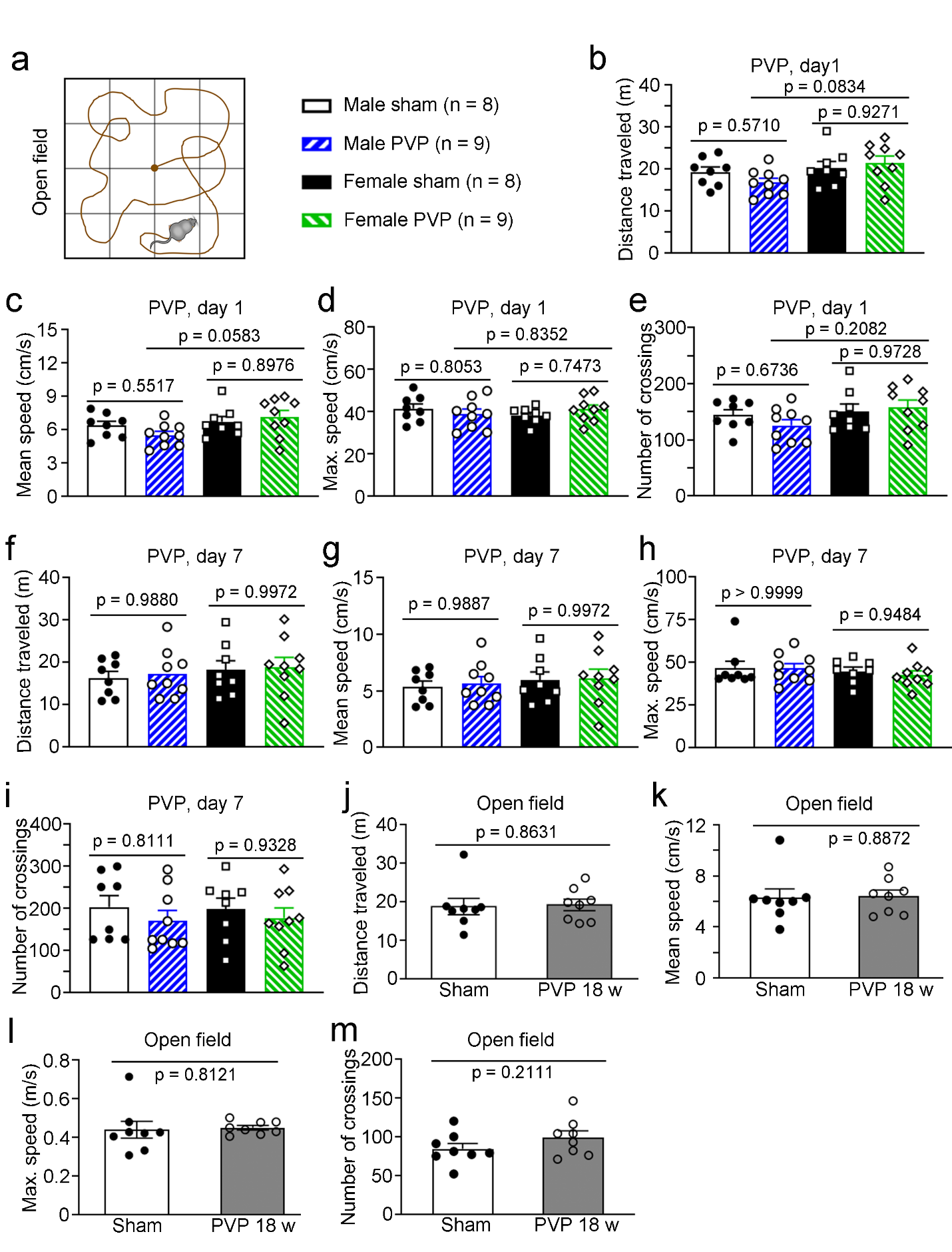


Figure. S5.

**Behavioral phenotype in locomotor activity of PVP mice. a** Schematic of the open field test. **b-m** Intact locomotor activity indicated by total distance traveled (**b**, **f**, **j**), mean moving speed (**c**, **g**, **k**), maximum moving speed (**d**, **h,** **l**) and the number of line crossings (**e**, **I**, **m**) of PVP mice in the early (**b**-**i**) and late (**j-m**) phases in the open field test. Maxi., maximum; PVP, peripheral vascular pain. n = 8~9 (b-i) and 8 (j-m) mice. The data are presented as the means ± SEM; statistical comparisons were conducted with one-way ANOVA with Tukey’s post hoc test (b-i) or unpaired two-tailed *t*-test (j-m).

Supplementary Figure 6


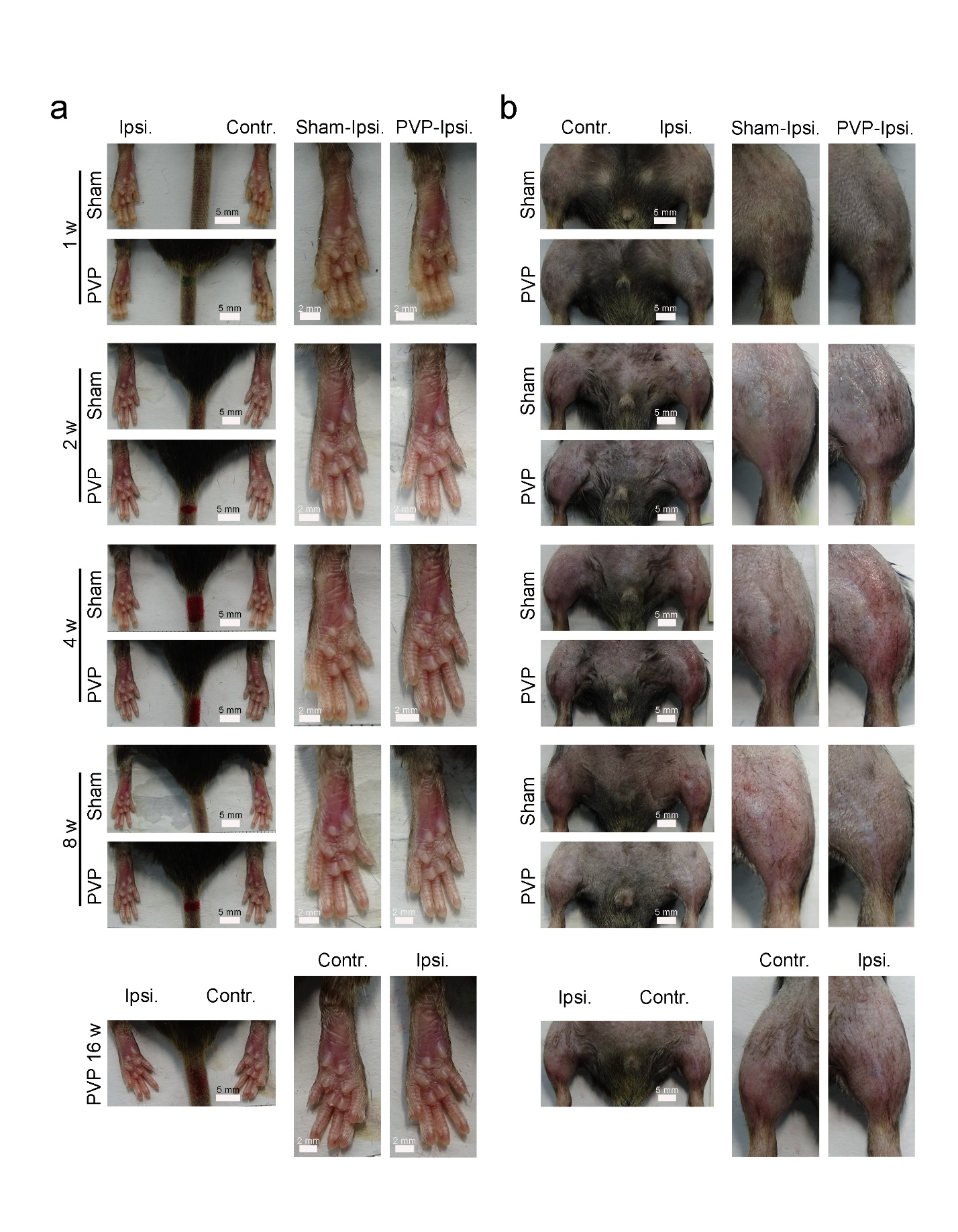


Figure. S6.

**No conspicuous pathology or abnormality in the plantar region and ligation region of the skin after vessel ligation. a** Normal appearance of the ipsilateral paws of PVP mice after vessel ligation, compared with the ipsilateral paws of sham control mice or the contralateral paws of PVP mice. **b** There was no conspicuous pathology in the ipsilateral ligation regions of the skin after vessel ligation compared with the ipsilateral regions of the skin in sham control mice or contralateral regions of PVP mice. Scale bars, 5 mm. contr., contralateral; ipsi., ipsilateral; PVP, peripheral vascular pain.

Supplementary Figure 7


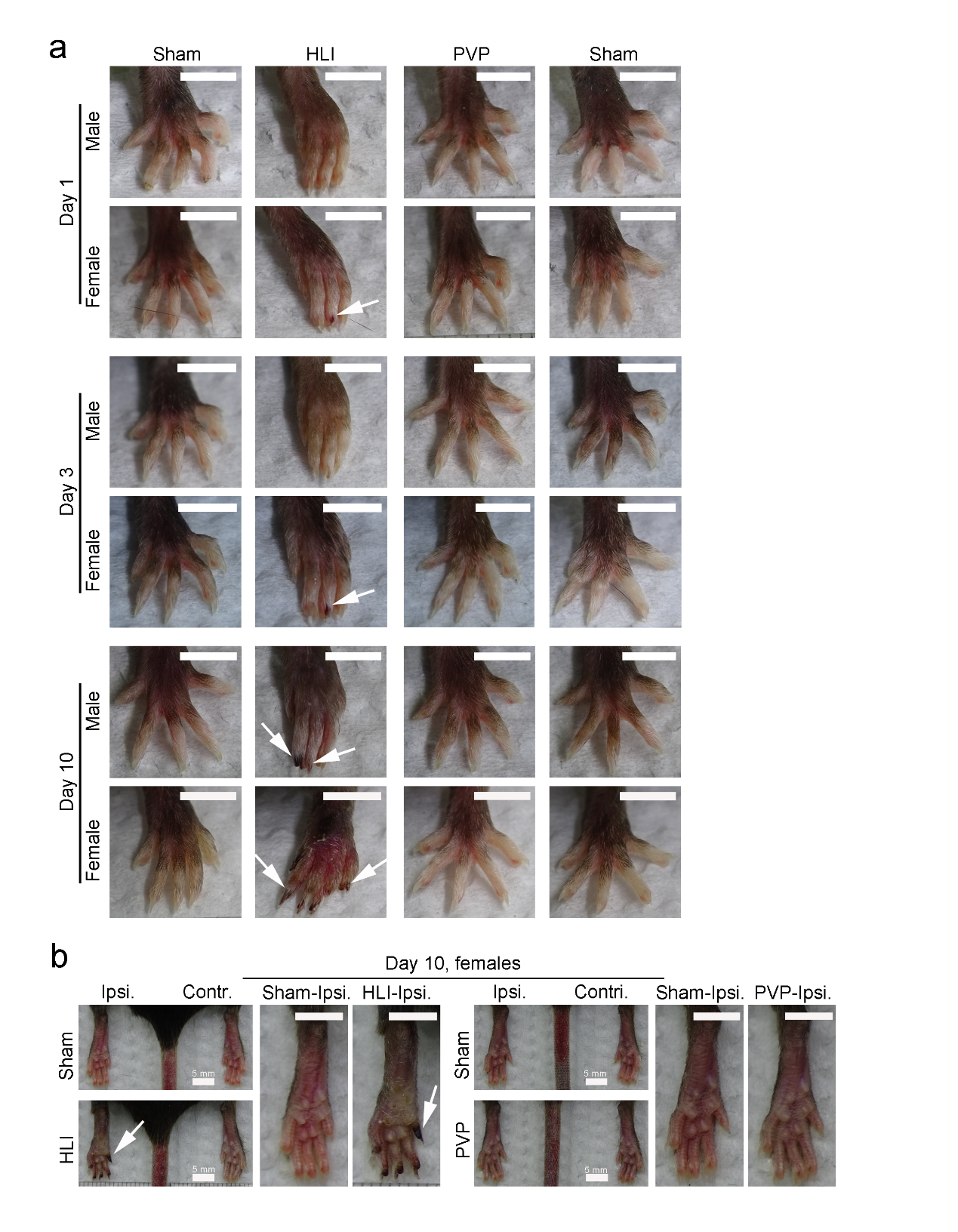


Figure. S7.

**Conspicuous pathology and abnormality in the ipsilateral hindpaws after HLI but not after the PVP operation. a** and **b** Paw abnormality and toe necrosis on day 1, 3 and 10 after the operation. Scale bars, 5 mm. Contr., contralateral; HLI, hindlimb ischemia; Ipsi., ipsilateral; PVP, peripheral vascular pain.

Supplementary Figure 8


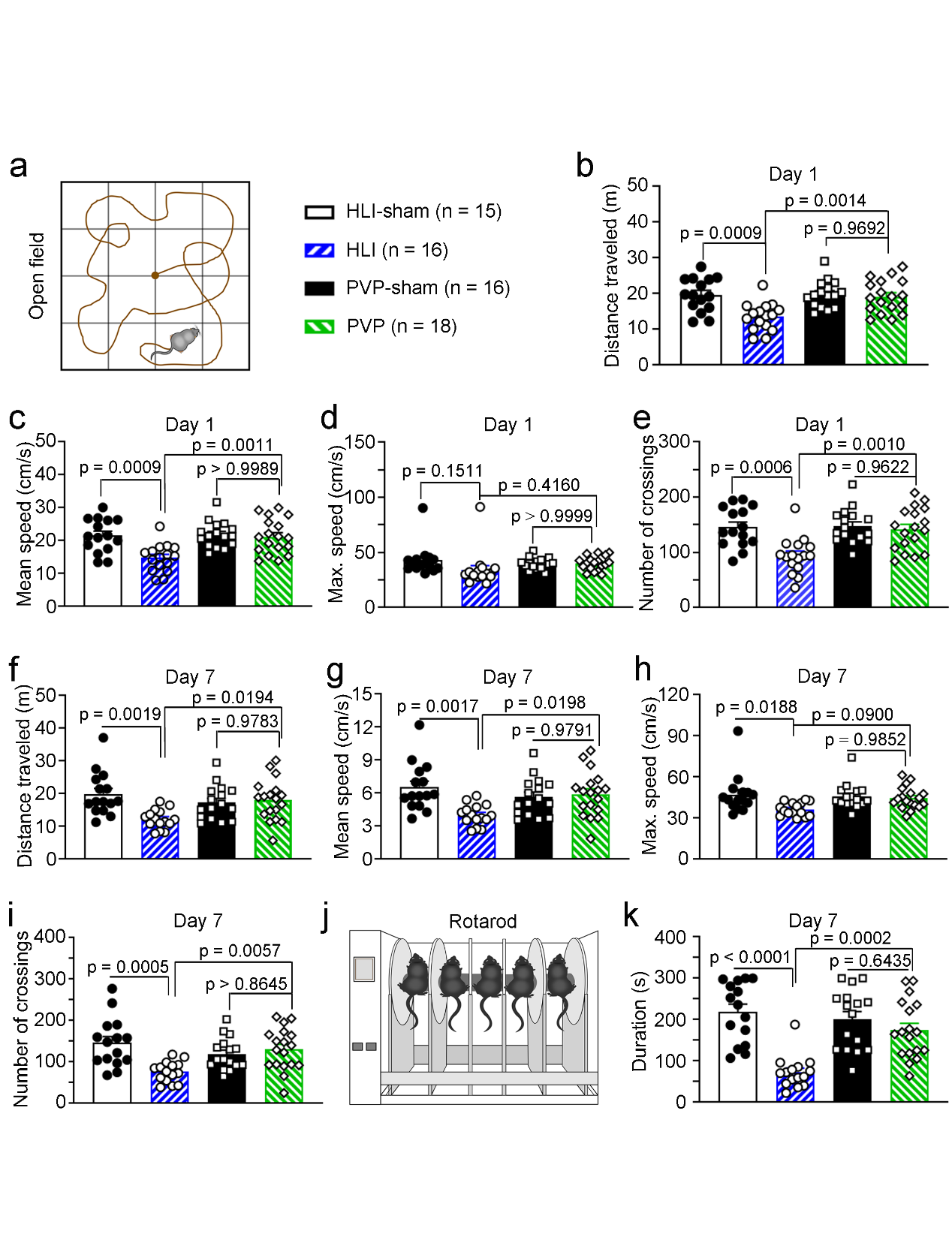


Figure. S8.

**Behavioral phenotypes of motor coordination and locomotor activity in HLI and PVP mice during the first week after the operation. a** Schematic of the open field test. **b-i** Impairment of locomotor activity in the open field test was observed in HLI mice but not in PVP mice within 1 week after the operation. **j** and **k** Schematic of the accelerating rotarod test (**j**) and impairment of motor function indicated by the movement duration of HLI mice but not PVP mice within the first week (**k**). HLI, hindlimb ischemia; Maxi., maximum; PVP, peripheral vascular pain. n = 15~18 mice. The data are presented as the means ± SEM; statistical comparisons were conducted with one-way ANOVA with Sidak’s post hoc test.

Supplementary Figure 9

**
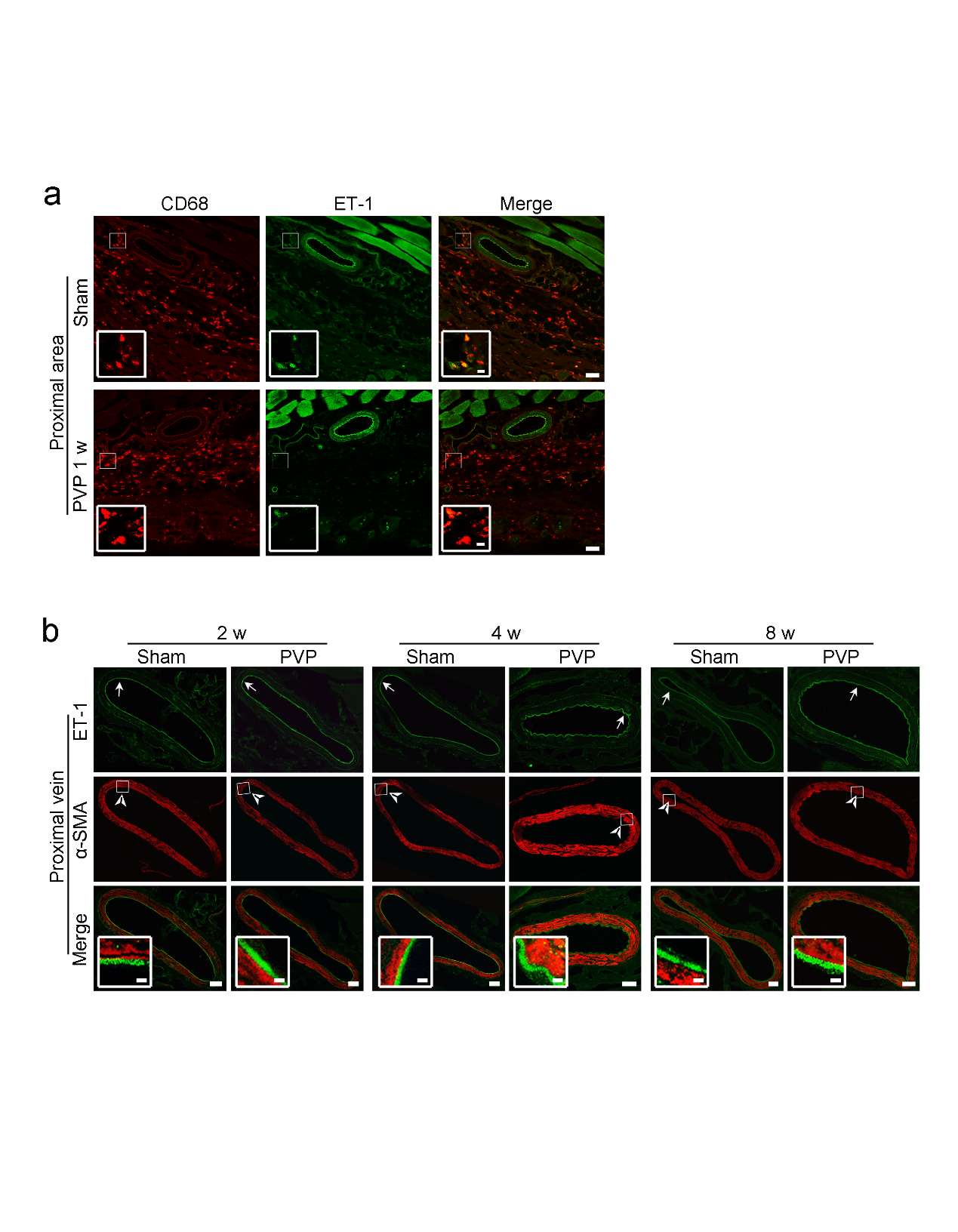
**

Figure. S9.

**The expression of ET-1 in the blood vessels and macrophages surrounding the tissues proximal to the ligation site during PVP. a** Representative images (20×) showing that there was little ET-1 expression on macrophages surrounding the tissues proximal to the ligation sites in mice with PVP. **b** There was no increase in ET-1 expression in the SMCs of the ligated veins proximal to the ligated sites of mice with PVP. Scale bars, 50 μm (a; 10 μm in boxes) or 20 μm (b). α-SMA, α-smooth muscle actin; ET-1, endothelin-1; PVP, peripheral vascular pain.

Supplementary Figure 10

**
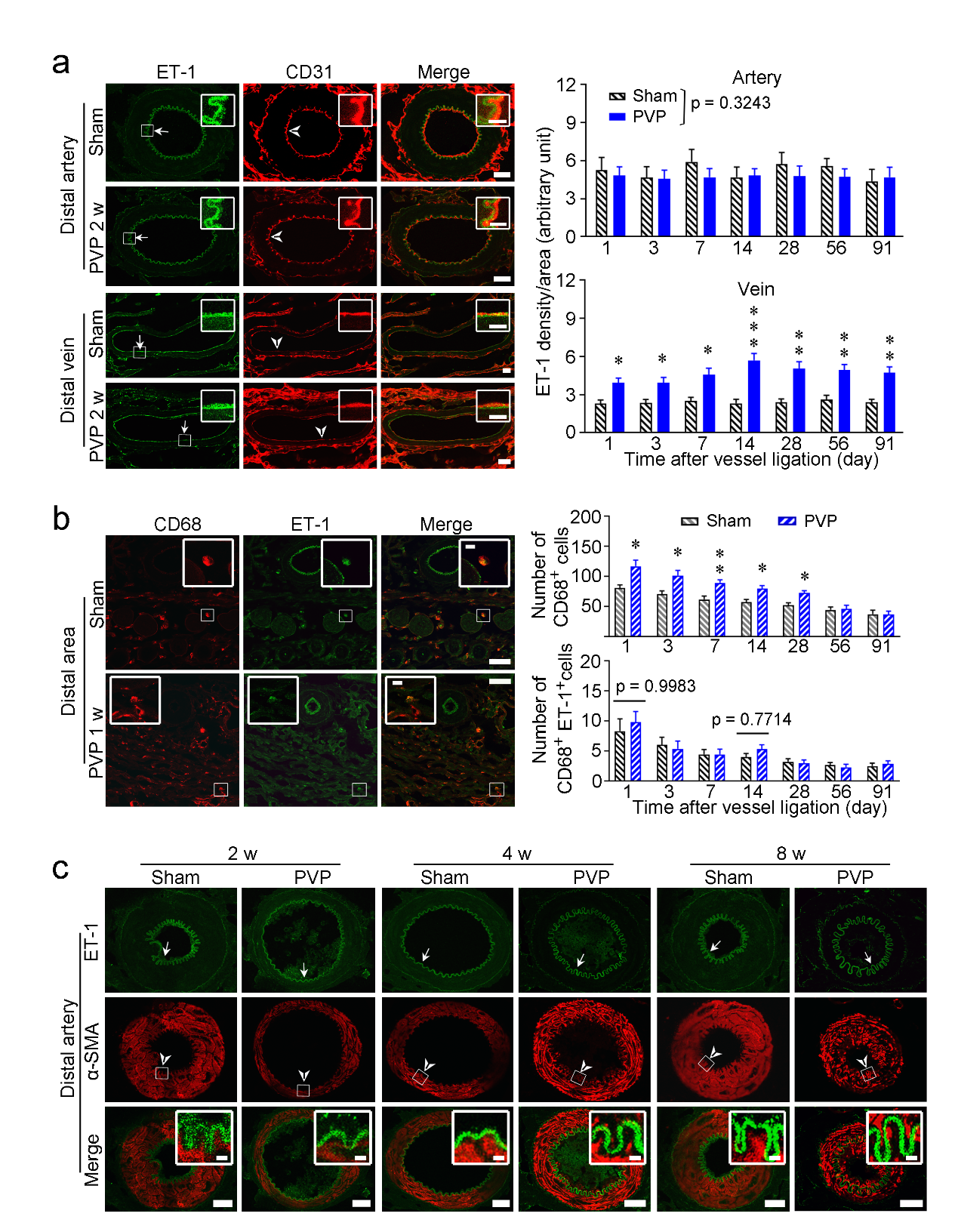
**

Figure. S10.

**The expression of ET-1 in the blood vessels and macrophages surrounding the tissues distal to the ligation site during PVP. a** Increased expression of ET-1 in ECs of the ligated veins but not arteries distal to the ligated sites in mice with PVP. Representative images indicating ET-1 expressed in ECs (**left**) and quantification of ET-1 expression (**right**). Arrows indicate ET-1 expressed in ECs; arrowheads indicate CD31 expressed in ECS. **b** There was little expression of ET-1 on macrophages surrounding the tissues distal to the ligated sites in mice with PVP. High-magnification (40×) representative images indicating low ET-1 expression on macrophages (**left**) and quantification of ET-1 expression (**right**). **c** There was no increase in ET-1 expression in the SMCs of the ligated arteries and veins distal to the ligated sites in mice with PVP. Scale bars, 20 μm (a, c; 10 μm in boxes) or 50 μm (b). α-SMA, α-smooth muscle actin; ET-1, endothelin-1; PVP, peripheral vascular pain. n = 12~16 (a) and 13~15 (b) sections from 5 mice. The data are presented as the means ± SEM. *p < 0.05, **p < 0.01 and ***p < 0.001; statistical comparisons were conducted with two-way ANOVA with Sidak’s post hoc test.

Supplementary Figure 11

**
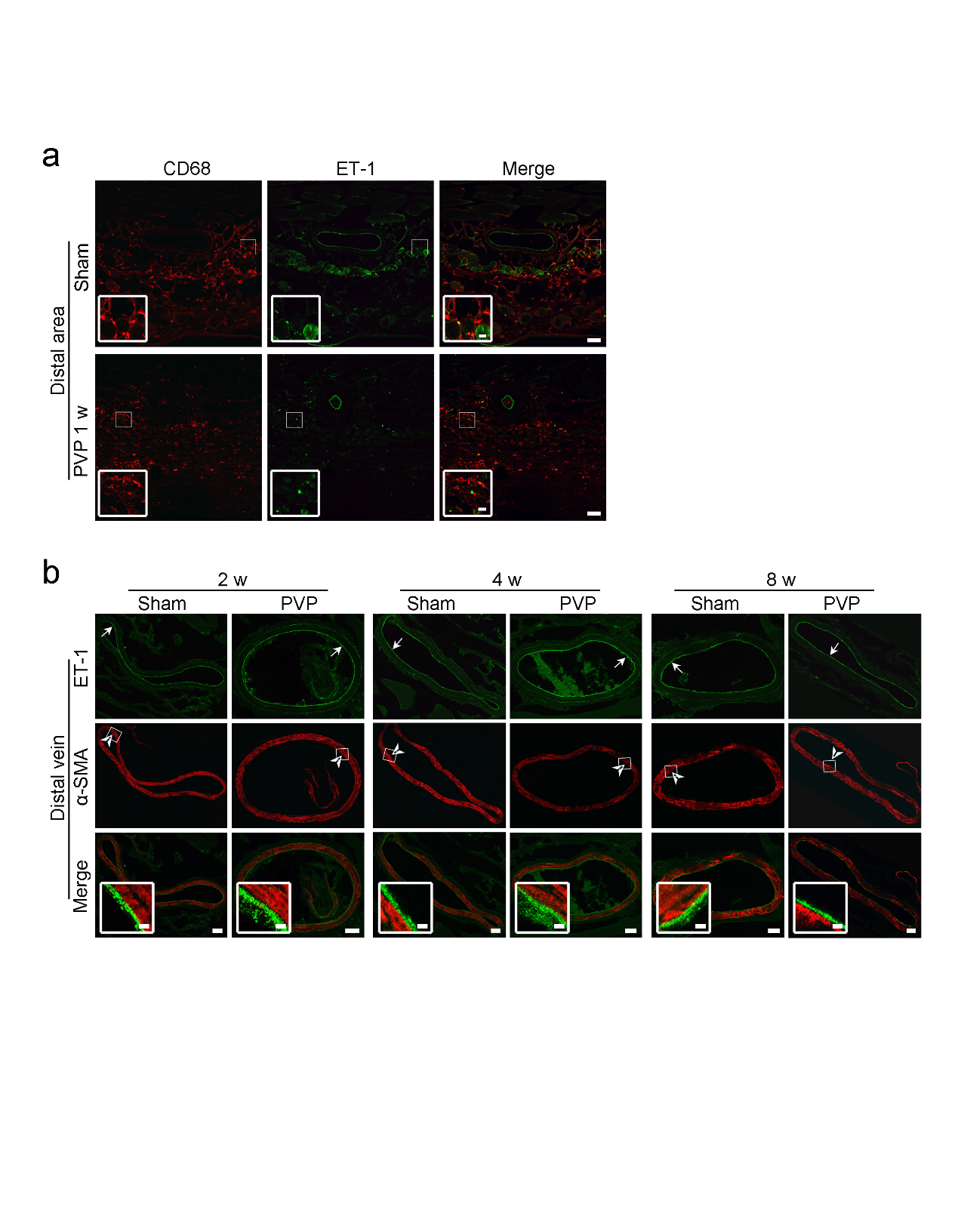
**

Figure. S11.

**The expression of ET-1 in the blood vessels and macrophages surrounding the tissues distal to the ligation site during PVP. a** Representative images (20 ×) showing that there was little ET-1 expressions on macrophages surrounding tissues distal to the ligated sites in mice with PVP. **b** There was no increase in ET-1 expression in the SMCs of the ligated veins distal to the ligated sites in mice with PVP. Arrows and arrowheads indicate the enlarged area in the boxes. Scale bars, 50 μm (a; 10 μm in boxes) or 20 μm (b). α-SMA, α-smooth muscle actin; ET-1, endothelin-1; PVP, peripheral vascular pain.

Supplementary Figure 12


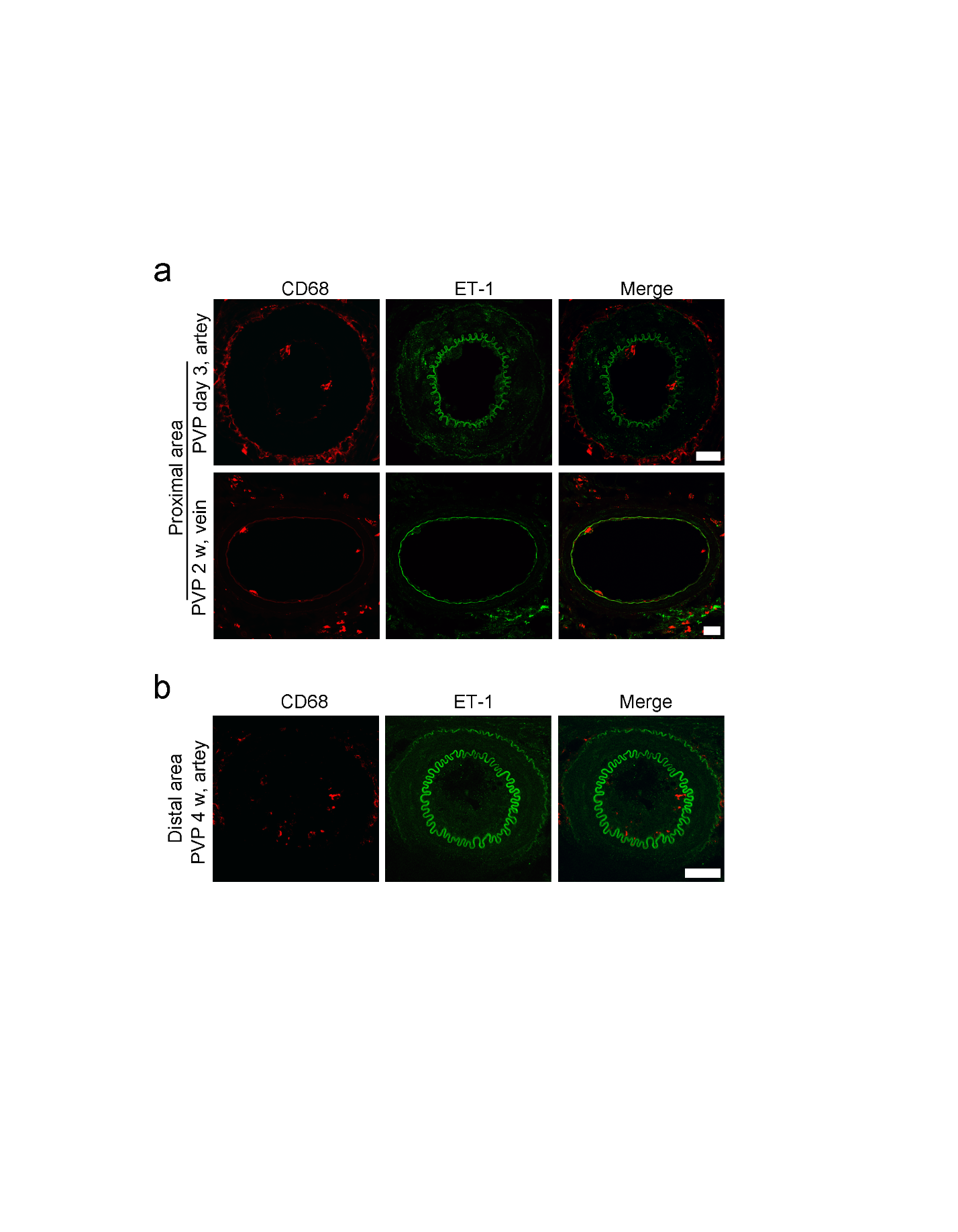


Figure. S12.

**Macrophage infiltration and secretion of ET-1 surrounding the tissues of the ligation site during PVP. a** and **b** Representative images (40 ×) indicating that there was little macrophage infiltration in vessel lumen proximal (**a**) and distal (**b**) to the ligated sites in mice with PVP or that underwent sham surgery. Few macrophages expressed ET-1. Scale bars, 50 μm. ET-1, endothelin-1; PVP, peripheral vascular pain.

Supplementary Figure 13


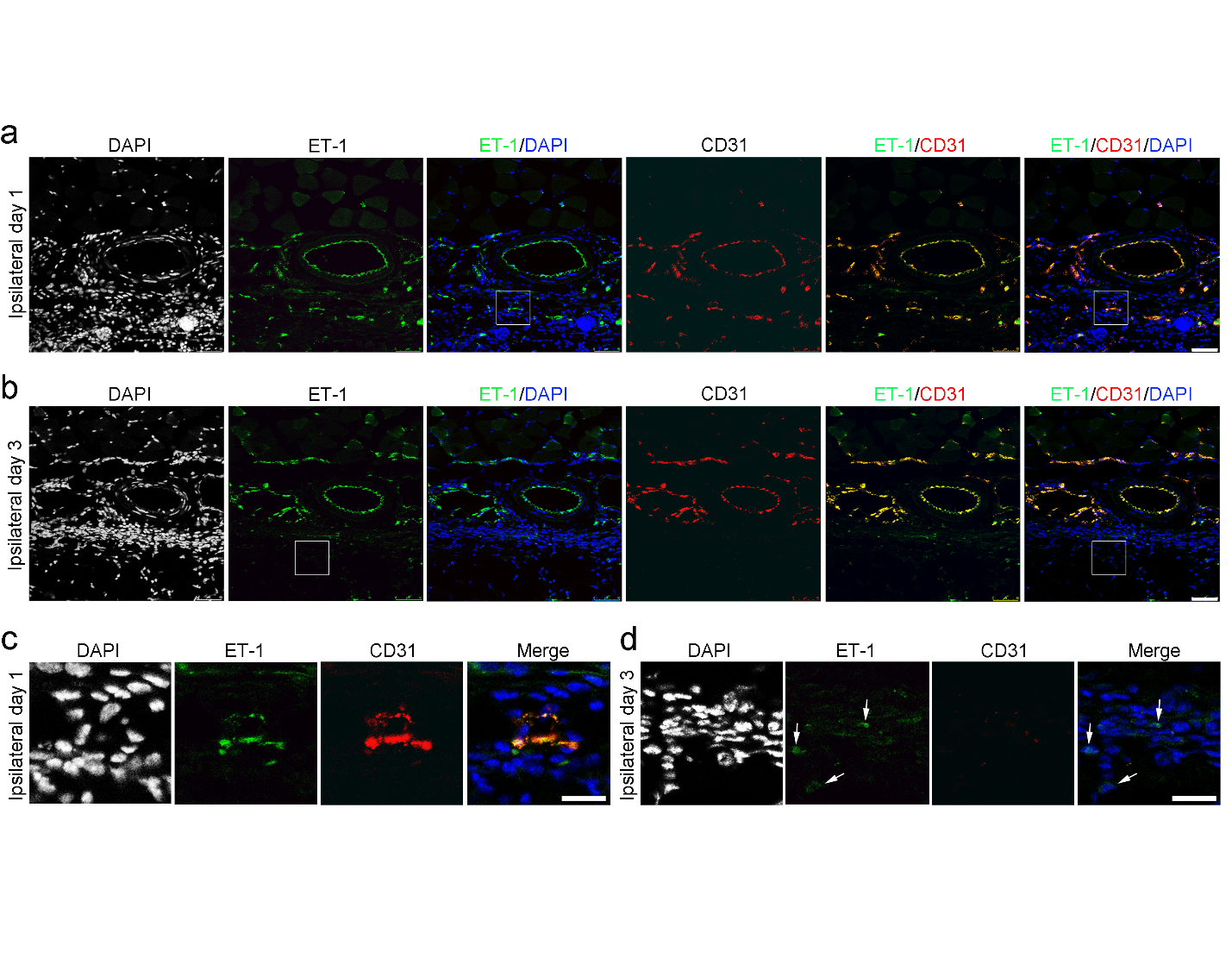


Figure. S13.

**The expression of ET-1 mRNA in the blood vessels and** **inflammatory cells surrounding the tissues of the ligation site during PVP. a-d** Representative images (40 ×) showing that there were mayorly ET-1 expressions on ECs in proximal vessels to the ligated sites on day 1 (**a, c**) and day 3 (**b, d**) in mice with PVP. **c, d** Image enlargements for **a, b**. There was little ET-1 mRNA expression on the infiltrated inflammatory cells (**d**) of the proximal to the ligated site in mice with PVP. Arrows indicate the expression of ET-1 on inflammatory cells. Scale bars, 50 μm (a, b) or 20 μm (c, d). ET-1, endothelin-1; PVP, peripheral vascular pain. n = 12~19 sections from 5 mice.

Supplementary Figure 14


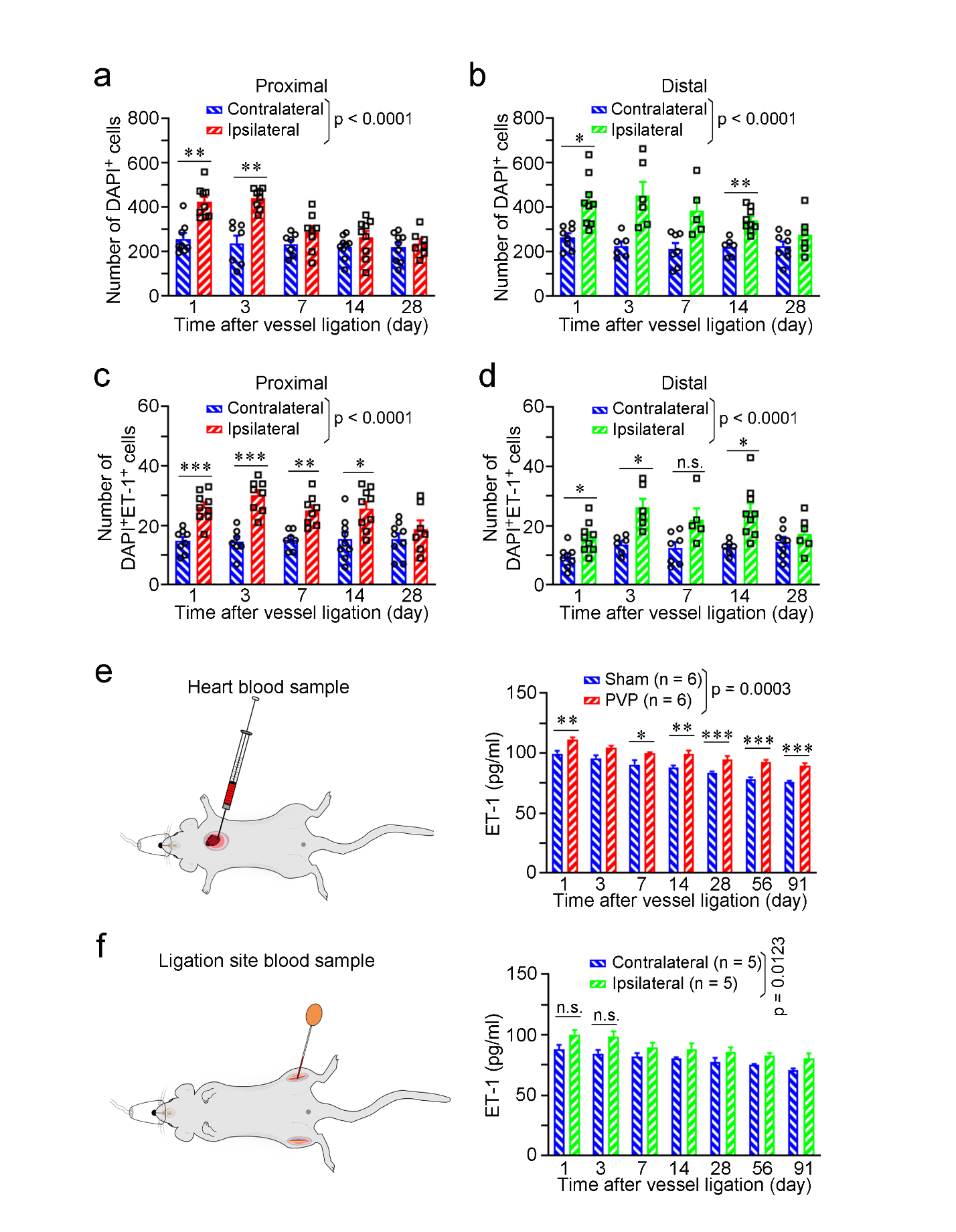


Figure. S14.

**The expression of ET-1 mRNA on inflammatory cells surrounding the tissues of the ligation site and serum ET-1 concentration in mice during PVP. a-d** Quantification of inflammatory cells (indicated by DAPI staining) infiltrated into the proximal area (**a, c**) and distal area (**b, d**), as well as their expression of ET-1 mRNA (**c, d**). **e, f** Quantification of serum ET-1 concentration in heart blood (**e**) or local blood sample around ligation site (**f**) measured by ELISA. ET-1, endothelin-1; n.s., non-significant; PVP, peripheral vascular pain. n = 7~9 (a-c) and 5~9 (d) sections from 3 mice, 6 (e) and 5 (f) mice. The data are presented as the means ± SEM. *p < 0.05, **p < 0.01 and ***p < 0.001; statistical comparisons were conducted with two-way ANOVA with Sidak’s post hoc test.

Supplementary Figure 15


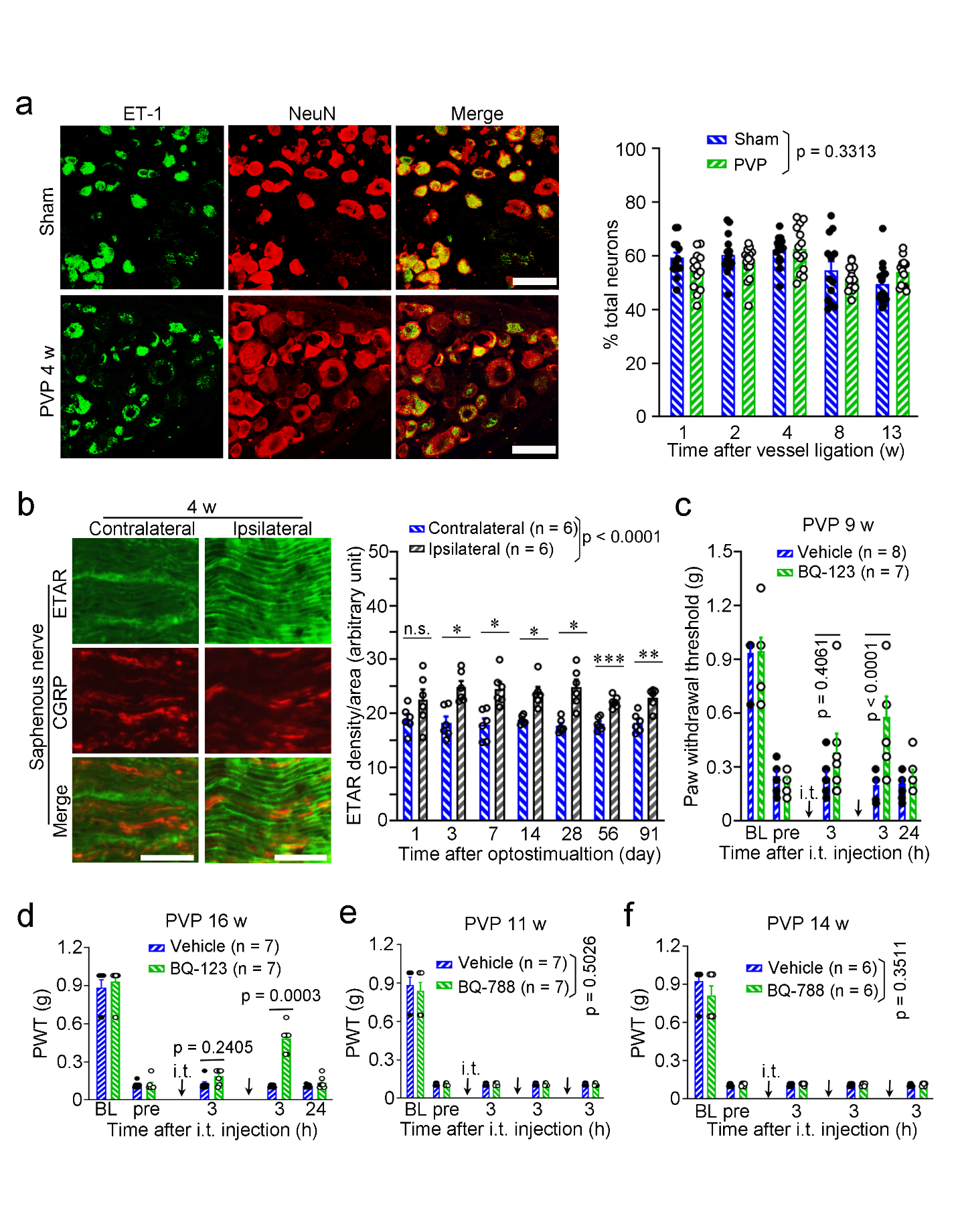


Figure. S15.

**The expression of neuronal ET-1/ETAR and activation of ETAR signaling during PVP. a** Neuronal ET-1 expression in the L4-5 DRGs did not differ between PVP mice and sham control mice. Representative images of the expression (**left**) and quantification (**right**) of ET-1. **b** Neuronal ETAR expression on the ipsilateral saphenous nerves was upregulated compared to that of the contralateral saphenous nerves of PVP mice. Representative images of the expression (**left**) and quantification (**right**) of ETAR. **c, d** Intrathecal blockade of ETAR with BQ-123 suppressed mechanical hyperalgesia in the middle (**c**) and late (**d**) phases in PVP mice. **e** and **f** Intrathecal blockade of ETBR with BQ-788 did not affect mechanical hyperalgesia in the middle (**e**) and late (**f**) phases in PVP mice. Scale bars, 50 μm. BL, baseline; CGRP, calcitonin gene-related peptide; ET-1, endothelin-1; ETAR, endothelin A receptor; ETBR, endothelin B receptor; i.t., intrathecal injection; L, lumbar; n.s., non-significant; pre, preadministration; PVP, peripheral vascular pain; PWT, paw withdrawal threshold. n = 13~15 (a), 6 (b) sections from 5 mice, and 7~8 mice (c-f). The data are presented as the means ± SEM. *p < 0.05, **p < 0.01 and ***p < 0.001; statistical comparisons were conducted with two-way ANOVA with Sidak’s post hoc test.

Supplementary Figure 16


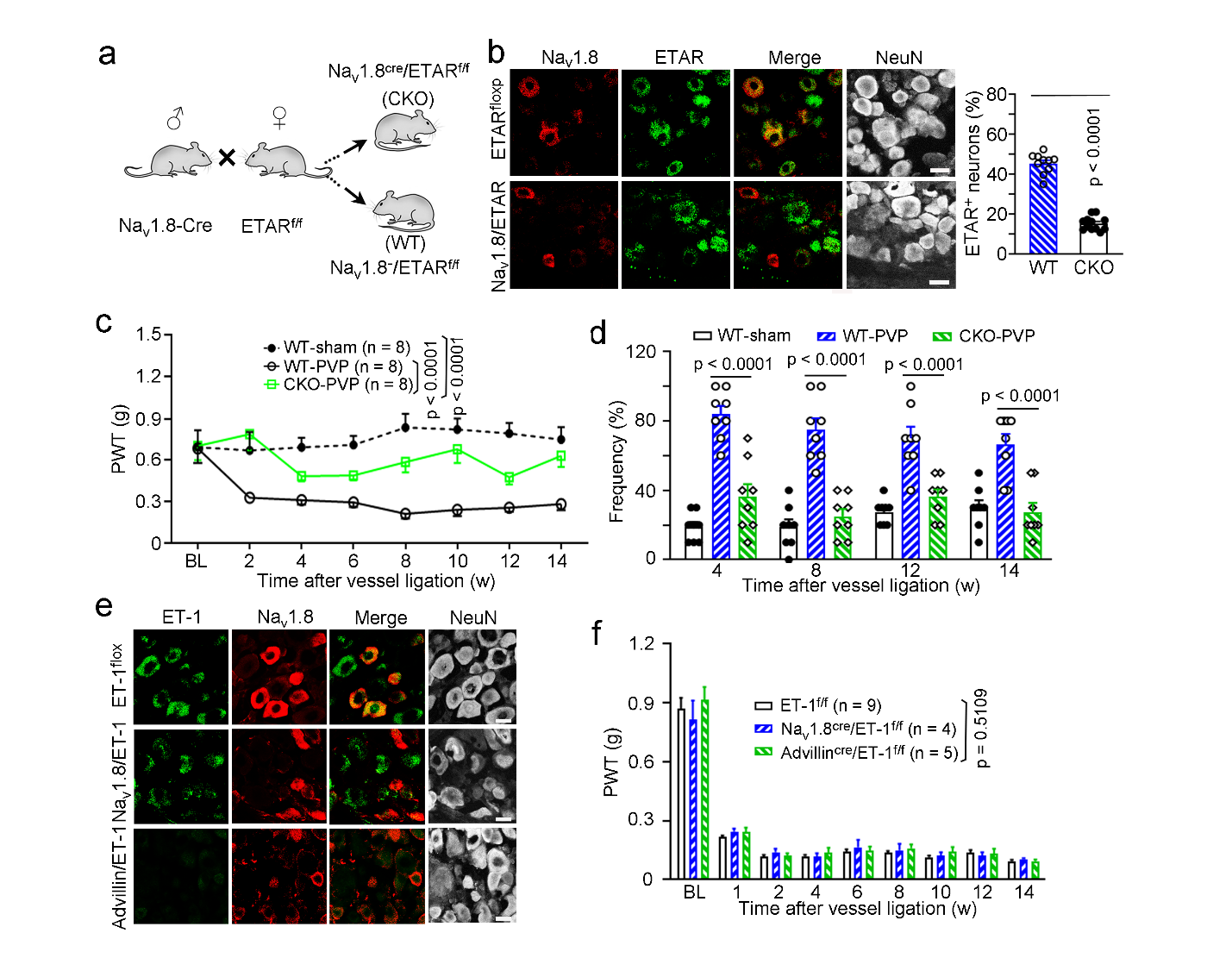


Figure. S16.

**Impaired mechanical hyperalgesia in ETAR CKO mice with PVP and intact mechanical hyperalgesia in ET-1 CKO mice with PVP. a** Schematic representation of Na_v_1.8^cre^/ETAR^f/f^ (CKO) mice generated by crossing specific nociceptor-dependent promoter (Na_v_1.8-Cre) mice with ETAR-flox mice. **b** The selective deletion of ETAR in the nociceptive neurons of CKO mice was confirmed through immunofluorescence (**left**) and by counting ETAR^+^ neurons (**right**). **c** and **d** Conditional deletion of the ETAR in nociceptors reduces mechanical hyperalgesia (indicated by the mechanical threshold; **c**) and mechanical allodynia (indicated by the 0.6 g hair-induced paw withdrawal frequency; **d**) in Na_v_1.8^cre^/ETAR^f/f^ mice with PVP. **e** and **f** Intact mechanical hyperalgesia in ET-1 CKO mice with PVP. Na_v_1.8^cre^/ET-1^f/f^ and advillin^cre^/ET-1^f/f^ CKO mice were validated by coexpression of ETAR with Na_v_1.8 and NeuN in the L4-5 DRGs (**e**) and these mice exhibited intact mechanical pain after vessel ligation as compared with that of ET-1^f/f^ PVP mice (**f**). Scale bar, 50 μm. BL, baseline; CKO, conditional knockout; ET-1, endothelin-1; ETAR, endothelin A receptor; L, lumbar; PVP, peripheral vascular pain; PWT, paw withdrawal threshold; WT, wild-type. n = 10~12 (b), sections from 3 mice, 8 (c, d) and 4~9 (f) mice. The data are presented as the means ± SEM; statistical comparisons were conducted with two-way ANOVA with Sidak’s post hoc test.

Supplementary Figure 17


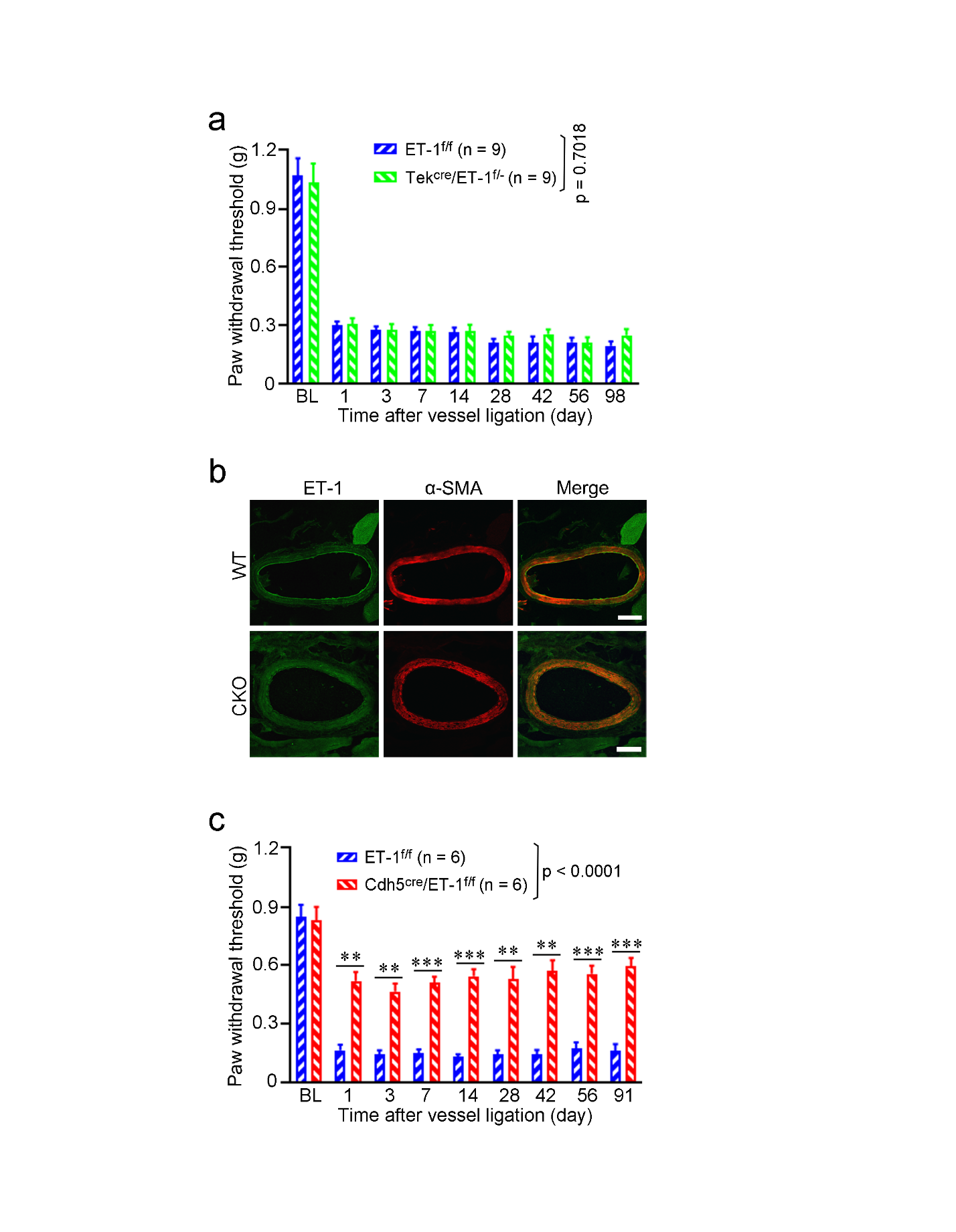


Figure. S17.

**The impairment of PVP after the conditional deletion of ET-1 in ECs. a** The heterozygotes of Tek^cre^/ET-1^f/-^ mice exhibited normal PVP after vessel ligation. **b** Representative immunofluorescence images showing selective deletion of ET-1 in ECs under Cdh5-Cre/ERT2 promoter (Cdh5^cre^/ET-1^f/f^ homozygotes) on day 14 following tamoxifen induction. **c** Partial impairment of PVP in Cdh5^cre^/ET-1^f/f^ mice following tamoxifen induction. Scale bars, 50 μm. α-SMA, α-smooth muscle actin; BL, baseline; CKO, conditional knockout; ET-1, endothelin-1; PVP, peripheral vascular pain; WT, wild-type. n = 9 (a) and 6 (c) mice. The data are presented as the means ± SEM; **p < 0.01 and ***p < 0.001; statistical comparisons were conducted with two-way ANOVA with Sidak’s post hoc test.

Supplementary Figure 18


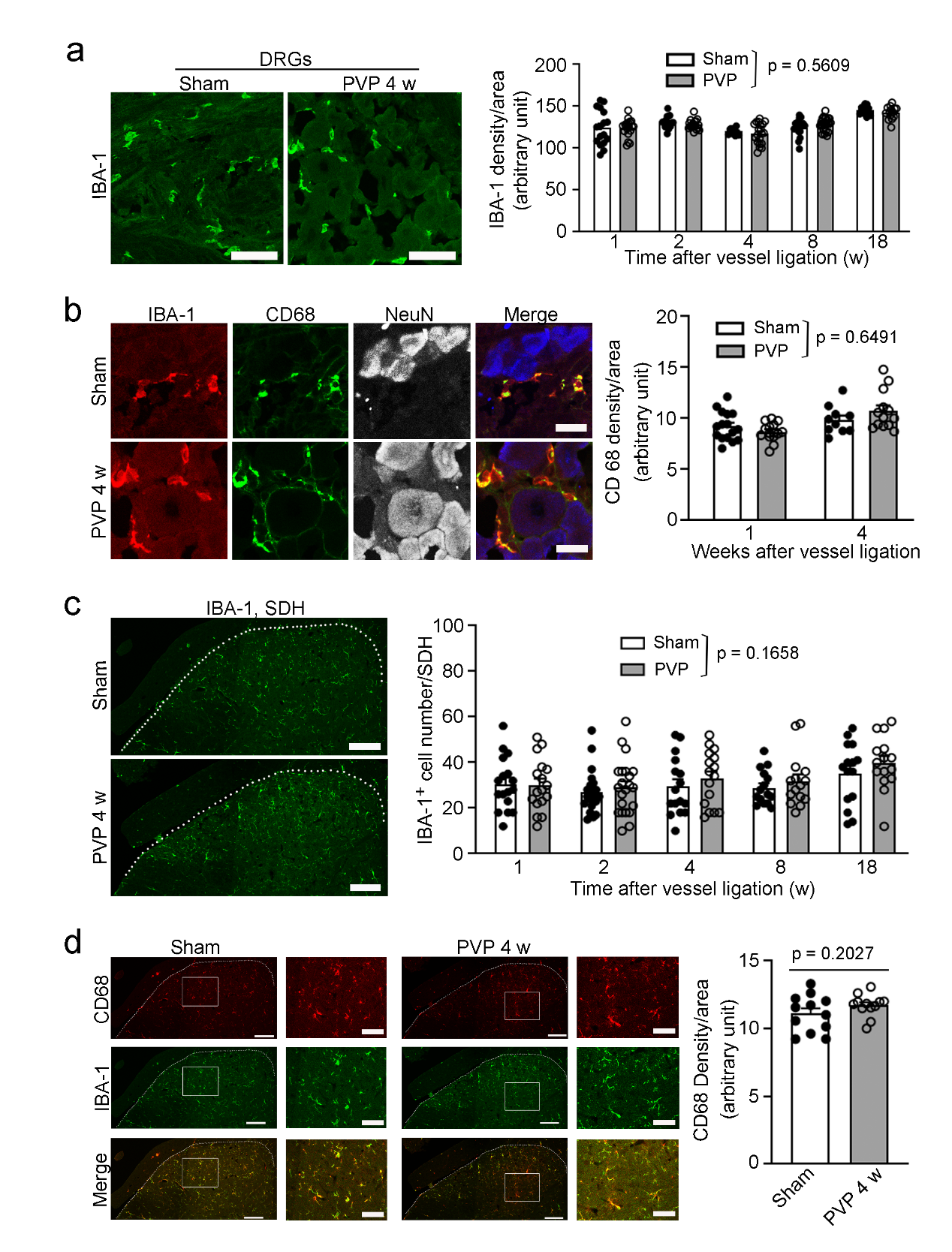


Figure. S18.

**The expression of IBA-1 and CD68 in the lumbar DRGs and SDH in mice with PVP. a** and **b** No differences in the expression of either IBA-1 (**a**) or CD68 (**b**) in the ipsilateral L4-5 DRGs were observed between PVP mice and sham control mice. Representative images of IBA-1 and/or CD68 expression (**left**) and quantification of IBA-1 or CD68 expression (**right**). **c** and **d** No differences in the expression of IBA-1 (**c**) or CD68 (**d**) in the ipsilateral L4-5 SDH were observed between PVP mice and sham control mice. Representative images of IBA-1 and/or CD68 expression (**left**) and quantification of IBA-1 or CD68 expression (**right**). Scale bars, 20 μm (**b**, **d** in boxes), 50 μm (**a**) or 100 μm (**c**, **d**). DRGs, dorsal root ganglia; IBA-1, ionized calcium-binding adapter molecule 1; L, lumbar; PVP, peripheral vascular pain; SDH, spinal cord horn. n = 11~25 (a), 13~16 (b), 15~22 (c) and 12 (d) sections from 5 mice. The data are presented as the means ± SEM; statistical comparisons were conducted with two-way ANOVA with Sidak’s post hoc test (a-c) or unpaired two-tailed *t*-test (d).

Supplementary Figure 19


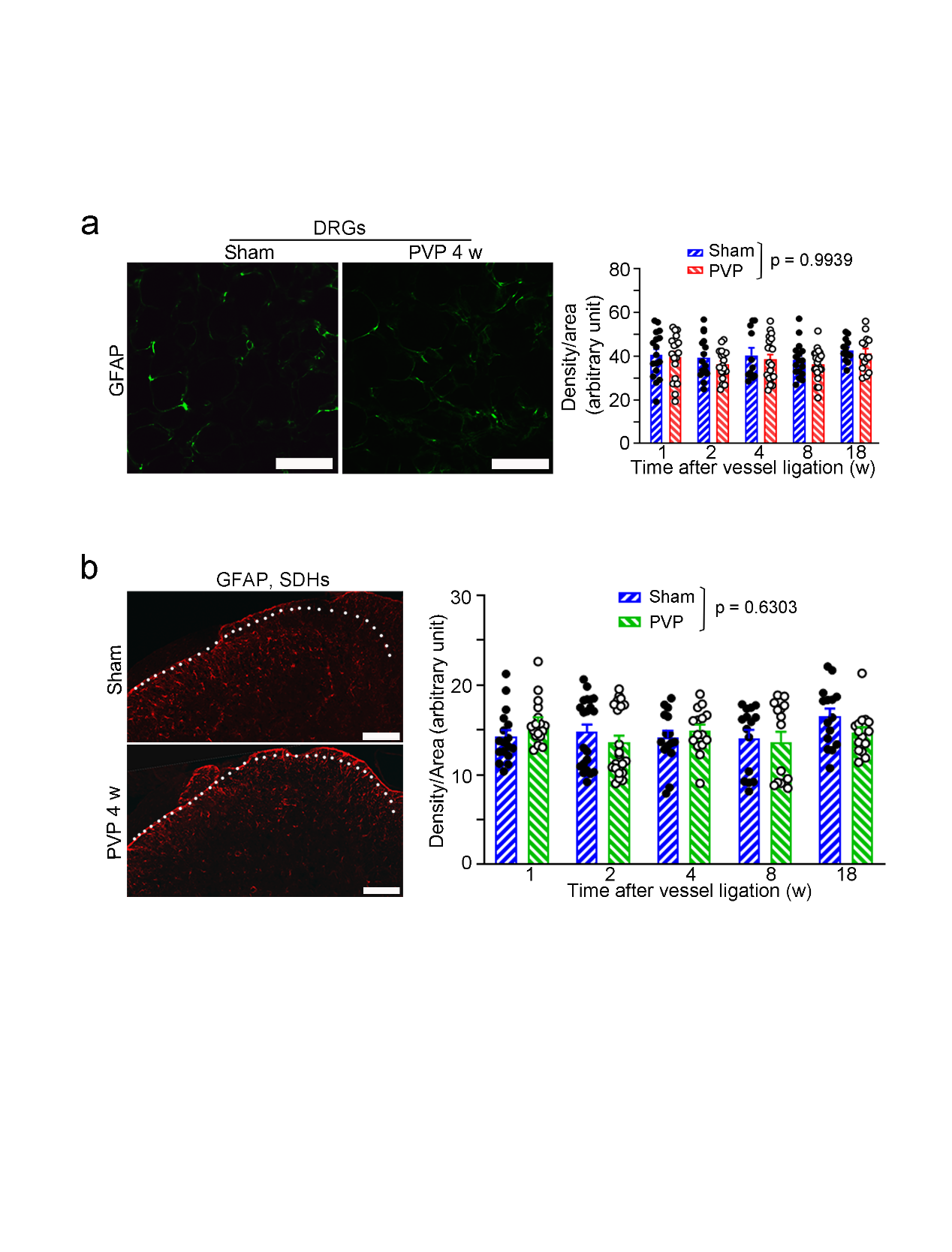


Figure. S19.

**The expression of GFAP in the lumbar DRGs and SDH of PVP mice. a** and **b** No differences in the expression of GFAP in the ipsilateral L4-5 DRGs (**a**) or SDH (**b**) were observed between PVP mice and sham control mice. Representative images of GFAP expression (**left**) and quantification of GFAP expression (**right**). Scale bars, 50 μm (a) or 100 μm (b). DRGs, dorsal root ganglia; GFAP, glial fibrillary acidic protein; L, lumbar; PVP, peripheral vascular pain; SDH, spinal cord horn. n = 11~25 (a) and 15~22 (b) sections from 5 mice. The data are presented as the means ± SEM; statistical comparisons were conducted with two-way ANOVA with Sidak’s post hoc test.

Supplementary Figure 20


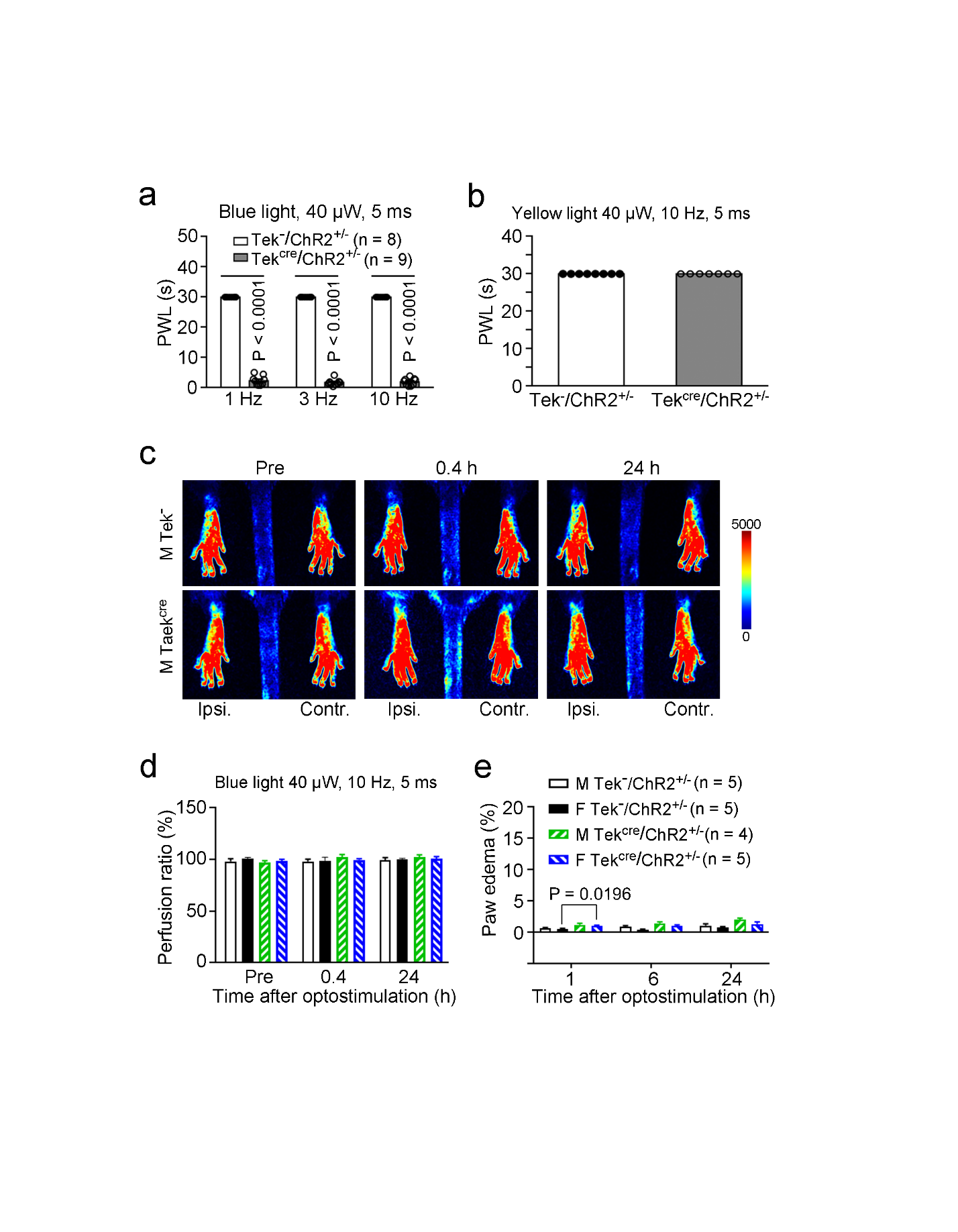


Figure. S20.

**Phenotypes associated with the pulse frequency-dependent effect, paw blood perfusion and edema in** **Tek^cre^/ChR2^+/-^ mice after optogenetic stimulation. a** No pulse frequency-dependent effect in Tek^cre^/ChR2^+/-^ mice on blue light stimulation. **b** Yellow light stimulation did not evoke an acute avoidance response in Tek^cre^/ChR2^+/-^ mice. **c** and **d** Blood flow in the optogenetic-manipulated paw and the subsequent recovery time course. Representative images indicating a normal perfusion (**c**) and no alteration within 24 hours in Tek^cre^/ChR2^+/-^ mice after a single session of blue light stimulation (**d**). **e** Tek^cre^/ChR2^+/-^ mice exhibited no obvious edema of the optogenetic-manipulated paws, compared with Tek^-^/ChR2^+/-^ mice after a single session of blue light stimulation. n = 8~9 (a), 7~8 (b) and 9~10 (d, e) mice. The data are presented as the means ± SEM; statistical comparisons were conducted with two-way ANOVA with Sidak’s post hoc test (a, d, e) or unpaired two-tailed *t*-test (b).

Supplementary Figure 21


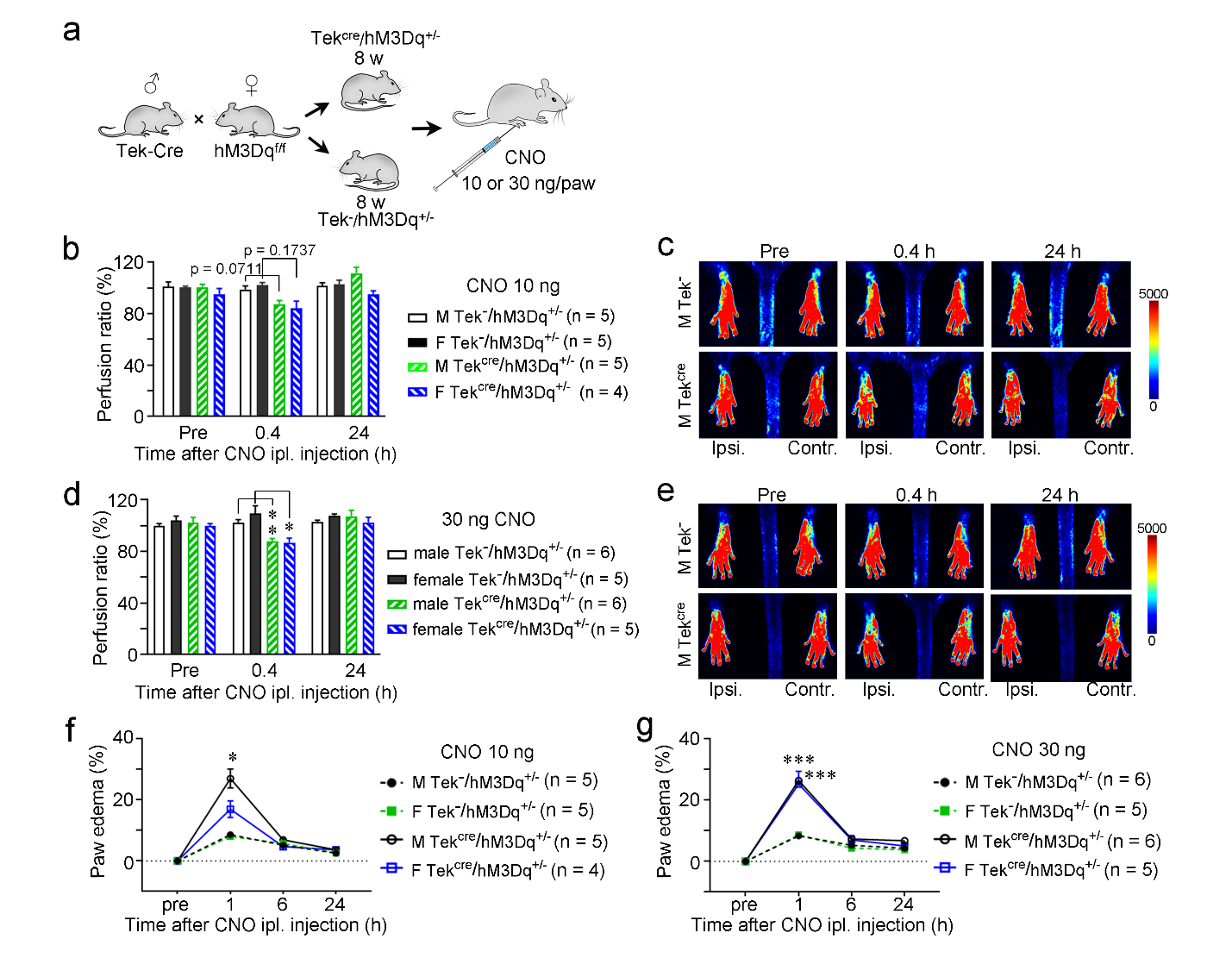


Figure. S21.

**Phenotypes of paw blood perfusion and edema in** **Tek^cre^/Hm3Dq^+/-^ mice after chemogenetic stimulation. a** Schematic of Tek^cre^/hM3Dq^+/-^ mouse generation by crossing EC-dependent promoter (Tek-Cre) mice with Hm3Dq-flox mice and optogenetic manipulation. **b**-**e** A slight reduction in blood flow in the paws of Tek^cre^/hM3Dq^+/-^ mice after 10 ng (**b, c**) or 30 ng **(d, e**) CNO intraplantar injection and the subsequent recovery time course. Representative images indicating a reduction in perfusion and a gradual recovery within 24 hours. **f**, **g** Significant edema of the ipsilateral paws in Tek^cre^/hM3Dq^+/-^ mice after a single intraplantar injection of 10 ng (**f**) or 30 ng (**g**) CNO and subsequent recovery within 24 hours. CNO, clozapine-N-oxide; contr., contralateral; F, female; hM3Dq, Gq-coupled human M3 muscarinic receptor; ipl., intraplantar injection; ipsi., ipsilateral; M, male; pre, preadministration; Tek^-^, Tek^-^/hM3Dq^+/-^; Tek^cre^, Tek^cre^/hM3Dq^+/-^. n = 4~5 (b, d) and 5~6 (e) mice. The data are presented as the means ± SEM; *p < 0.05 and ***p < 0.001, male or female Tek^cre^/hM3Dq^+/-^ compared with male or female Tek^-^/hM3Dq^+/-^, respectively; statistical comparisons were conducted with two-way ANOVA with Sidak’s post hoc test.

Supplementary Figure 22


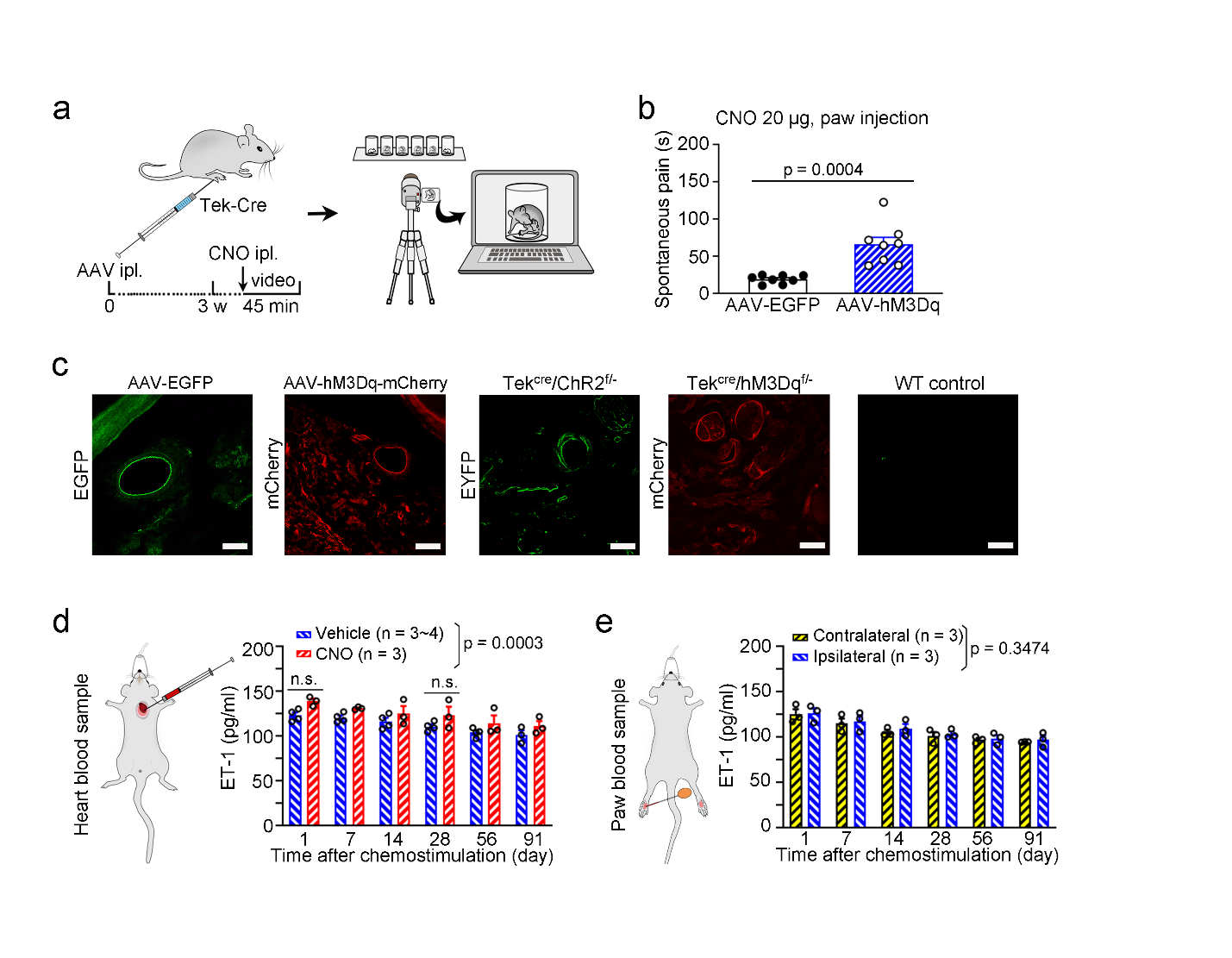


Figure. S22.

**Chemogenetic operation with AAV injection in Tek-Cre mice and serum ET-1 in chemogenetic mice after ECs activation. a**, **b** Schematic of the generation of Tek^cre^/AAV-Hm3Dq^+/-^ mice with an EC-dependent promoter (Tek-Cre) by intraplantar injection with AAV2/9-EF1a-DIO-hM3D(Gq)-mCherry-WPRE, allowing selective chemogenetic activation of ECs upon CNO administration (**a**) and acute avoidance responses evoked by a single CNO intraplantar administration (**b**). **c** Verification of paw infection by AAV in Tek-Cre mice and the expression of EGFP, EYFP or mCherry in the vessels of paw skin in Tek^cre^/ChR2^+/-^ or Tek^cre^/hM3Dq^+/-^ mice, respectively. **d**, **e** Quantification of serum ET-1 concentration in heart blood (**d**) or paw local blood samples (**e**) measured by ELISA for chemogenetic mice following ECs activation. Scale bars, 20 μm. AAV, adeno-associated virus; ChR2, channelrhodopsin-2; CNO, clozapine-N-oxide; ET-1, endothelin-1; EYFP, enhanced yellow fluorescence protein; EGFP, enhanced green fluorescence protein; hM3Dq, Gq-coupled human M3 muscarinic receptor; Ipl., intraplantar injection; n.s., non-significant; PWL, paw withdrawal latency. n = 8 (b) and 6 (e, f) mice. The data are presented as the means ± SEM; statistical comparisons were conducted with unpaired two-tailed *t*-test (b, f) or two-way ANOVA with Sidak’s post hoc test (e).

Supplementary Figure 23


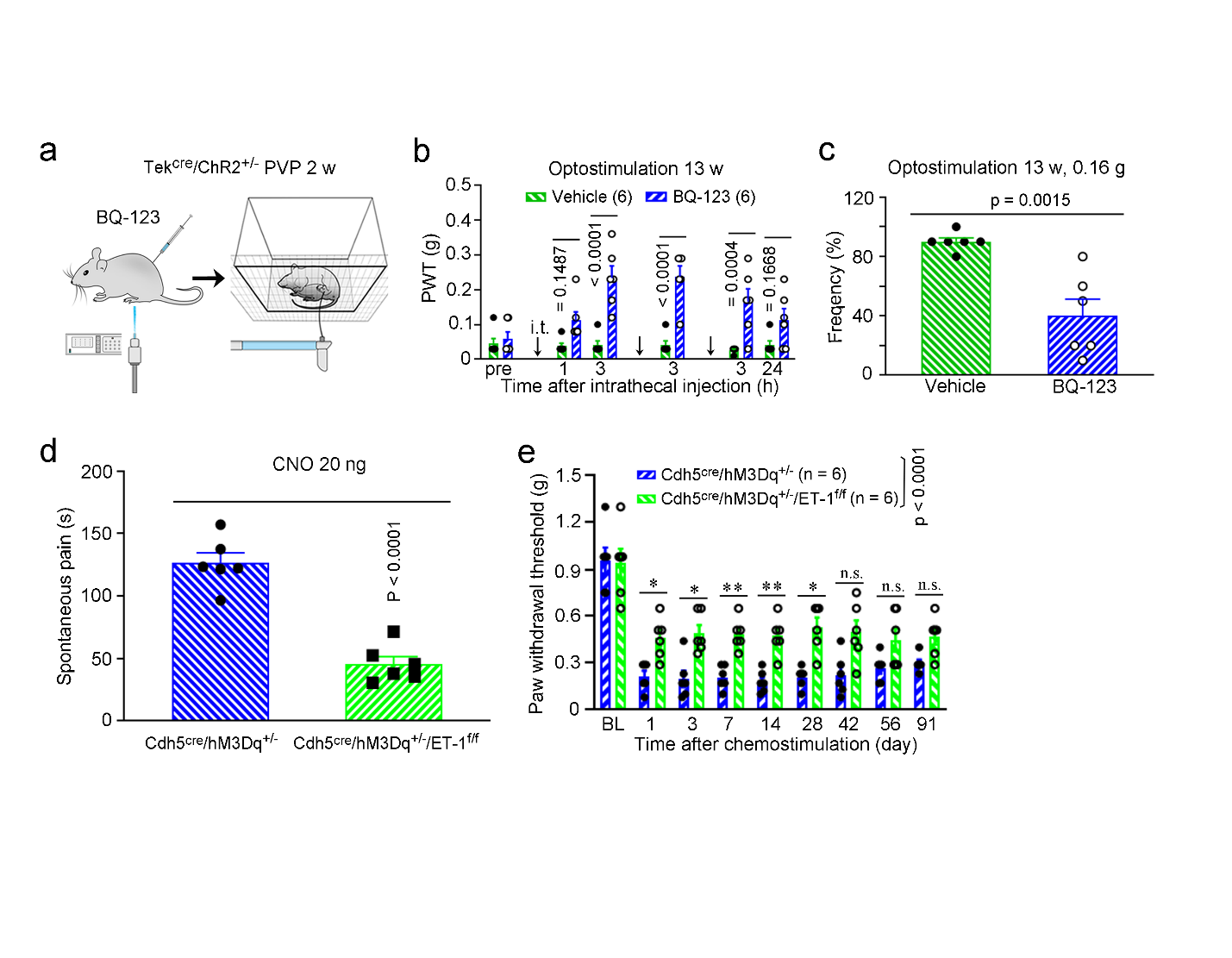


Figure. S23.

**The impairment of PVP after pharmacologically blocking ETAR in optogenetic mice and the conditional deletion of ET-1 in ECs in chemogenetic mice. a-c** The schematic (**a**), local administration of BQ-123 inhibited established long-lasting mechanical hyperalgesia (**b**) and allodynia (**c**) induced by blue light stimulation in Tek^cre^/ChR2^+/-^ mice. **d**, **e** Partial impairment of spontaneous pain (**d**) and chronic PVP (**e**) in Cdh5^cre^/hM3Dq^+/-^/ET-1^f/f^ mice following tamoxifen induction and the paw injection of CNO. BL, baseline; ChR2, channelrhodopsin-2; CKO, conditional knockout; ET-1, endothelin-1; ETAR, endothelin A receptor; n.s., non-significant; pre, pre-administration; PVP, peripheral vascular pain. n = 3~4 (b, c), 3 (e, f) and 6 (g, h) mice. The data are presented as the means ± SEM; *p < 0.05 and **p < 0.01; statistical comparisons were conducted with two-way ANOVA with Sidak’s post hoc test (b, e) or unpaired two-tailed *t*-test (c, d).

Supplementary Figure 24


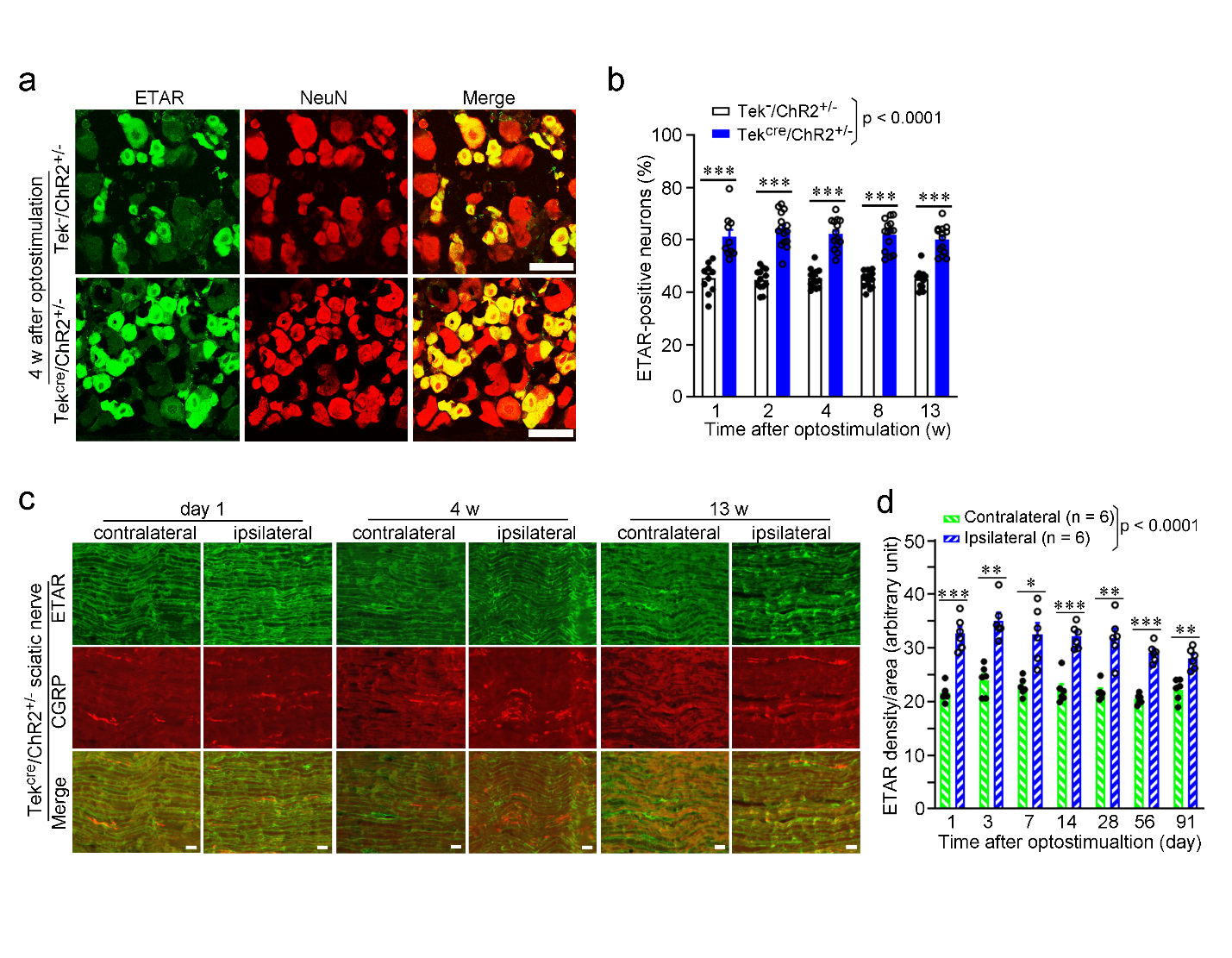


Figure. S24.

**Expression of the neural ETAR in primary sensory neurons and on their fibers in the optogenetic PVP mice. a, b** Increased expression of ETAR, which colocalized with NeuN, in the L4-5 DRGs of Tek^cre^/ChR2^+/-^ mice, compared with that in Tek^-^/ChR2^+/-^ control mice, after blue light stimulation. Representative images indicating the coexpression of ETAR with NeuN (**a**) and quantification of the coexpression (**b**). c, d Increased expression of ETAR on the ipsilateral sciatic nerves compared to that of the contralateral sciatic nerves in Tek^cre^/ChR2^+/-^ mice after blue light stimulation. Representative images (**c**) and quantification of the expression (**d**). Scale bars, 50 μm. ChR2, channelrhodopsin-2; ETAR, endothelin A receptor; PVP, peripheral vascular pain. n = 10~15 (b) from 5 mice and 6 section from 3 mice (d). The data are presented as the means ± SEM; statistical comparisons were conducted with two-way ANOVA with Sidak’s post hoc test.

Supplementary Figure 25


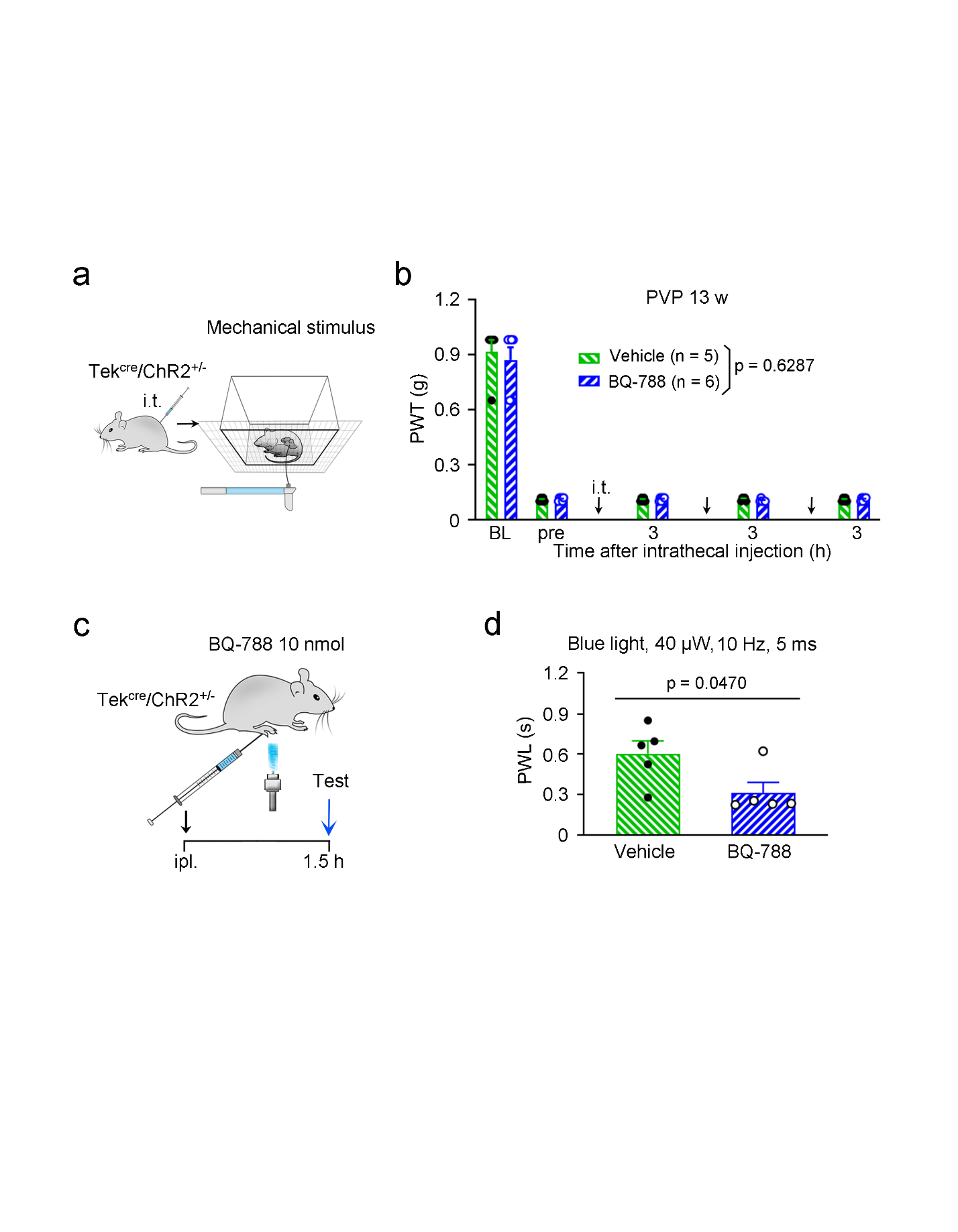


Figure. S25.

**Different functions of the neural and peripheral ETBR in the optogenetic PVP model. a, b** The schematic (**a**) and PWT (**b**) of local administration of BQ-788 in Tek^cre^/ChR2^+/-^ mice at 13 w after optostimulation. **c**, **d** The schematic (**c**) and the PWL (**d**) of local pre-administration of BQ-788 in Tek^cre^/ChR2^+/-^ mice. Scale bars, 50 μm. ChR2, channelrhodopsin-2; ETAR, endothelin A receptor; Ipl., intraplantar injection; PVP, peripheral vascular pain; PWL, paw withdrawal latency; PWT, paw withdrawal threshold. n = 5~6 (b) and 5 (d) mice. The data are presented as the means ± SEM; statistical comparisons were conducted with two-way ANOVA with Sidak’s post hoc test (b) or unpaired two-tailed *t*-test (d).

Table S1.

**Genotyping Methods**.

| Strain | Primers (5’- **>** -3’) | Wild type (WT) | Mutant/transgene (MT) | |
| --- | --- | --- | --- | --- |
| Na_v_1.8-Cre | F: ATT TGC CTG CAT TAC CGG TC  R: GCA TCA ACG TTT TCT TTT CGG |  | | 350 bp |
| Tek-Cre/Tie2-Cre (strain 004128) | F: ATT TGC CTG CAT TAC CGG TC  R: ATC AAC GTT TTC TTT TCG G |  | | 350 bp |
| ETAR-flox | F: CCT CAG GAA GGA AGT AGC AAG ATT A  R: ACA CAA CCA TGG TGT CGA | 610 bp | | 650 bp |
| Ai32 (B6:129) | F(WT): AAG GGA GCT GCA GTG GAG TA  R(WT): CCG AAA ATC TGT GGG AAG TC  F(MT): ACA TGG TCC TGC TGG AGT TC  R(MT): GGC ATT AAA GCA GCG TAT CC | 212 bp | | 297 bp |
| ROSA26-CAG-flex  -Hm3Dq-EYFP-flox | F: AGT CGC TCT GAG TTG TTA TCA G  R(WT): TGAGCATGTCTTTAATCTACCTCGATG  R(MT): GTCAATGGAAAGTCCCTATTGGCGT | 469 bp | | 278 bp |
| Advillin-Cre | F: GCAATTTCGACCAGGTT  R: GAGTCATCCTTAGCGCCGTA |  | | 420 bp |
| ET-1-flox | F: CCCAAAGATTCTGAATTGATAACTTCG  R: GATGATGTCCAGGTGGCAGAAG |  | | 900 bp |
| Tie2-Cre (strain 008863) | F(WT): CAT GGC CAC AGA ATT GAA AGA TCT  R(WT): GTA GGT GGA AAT TCT AGC ATC ATC C  F(MT): GCG GTC TGG CAG TAA AAA CTA TC  R(MT): GTG AAA CAG CAT TGC TGT CAC TT | 324 bp | | 100 bp |
| Cdh5-Cre/ERT2 | F: CCG GTC GAT GCA ACG AGT GAT GAG G  R: GCC TCC AGC TTG CAT GAT CTC CGG |  | | ~900 bp |

Table S2.

**Demographic data in two groups (Control group n = 45 and Tracleer group n = 44)**.

| Variable | Control Group | Tracleer Group | P value |
| --- | --- | --- | --- |
| Age (year) | 59.27±12.14 | 57.93±10.89 | 0.59 |
| Gender (M/F) | 29/16 | 28/16 | 0.94 |
| Height (cm) | 160(155-169) | 162(157-168) | 0.26 |
| Weight (kg) | 66.40±9.08 | 66.82±9.70 | 0.83 |
| Operation time (min) | 126(120-132) | 127(120-134) | 0.88 |
| Tourniquet time (min) | 70(65-78) | 70(65-78) | 0.85 |
| SBP (mmHg) | 130.57±9.34 | 130.07±9.59 | 0.80 |
| DBP (mmHg) | 71(65-78) | 73(65-77) | 0.94 |
| HR (bpm) | 72(68-76) | 72(68-78) | 0.50 |

Table S3.

**Statistics information**

| Figure | Panel | Mice | Time  points | Statistical comparison | P value |
| --- | --- | --- | --- | --- | --- |
| Figure 1 | C | Sham = 7 males  PVP = 15 males |  | two-way ANOVA with  Sidak's multiple comparisons  post hoc test | F(1,20)= 143.0  P<0.0001 |
|  |  |  | Baseline |  | >0.9999 |
|  |  |  | Day 1 |  | =0.0020 |
|  |  |  | Day 3 |  | =0.0002 |
|  |  |  | Day 7 |  | <0.0001 |
|  |  |  | Day 14 |  | <0.0001 |
|  |  |  | Day 28 |  | <0.0001 |
|  |  |  | Day 56 |  | <0.0001 |
|  |  |  | Day 84 |  | <0.0001 |
|  |  |  | Day 112 |  | <0.0001 |
|  | D | Sham = 7 males  PVP = 15 males |  | two-way ANOVA with  Sidak's multiple comparisons  post hoc test | F (1, 20) = 0.04137  P=0.8409 |
|  |  |  | Baseline |  | >0.9999 |
|  |  |  | Day 8 |  | =0.8626 |
|  |  |  | Day 15 |  | >0.9999 |
|  |  |  | Day 29 |  | =0.9571 |
|  |  |  | Day 43 |  | >0.9999 |
|  |  |  | Day 57 |  | =0.9968 |
|  |  |  | Day 85 |  | >0.9999 |
|  |  |  | Day 113 |  | 0.9912 |
|  | E | Sham = 7 males  PVP = 15 males |  | two-way ANOVA with  Sidak's multiple comparisons  post hoc test | F (1, 20) = 0.1809  P=0.6751 |
|  |  |  | 4 w |  | >0.9999 |
|  |  |  | 6 w |  | >0.9999 |
|  |  |  | 8 w |  | =0.9838 |
|  |  |  | 12 w |  | =0.2647 |
|  |  |  | 16 w |  | >0.9999 |
|  | F | SHam = 7 males + 10 females  PVP = 7 males + 10 females | | Unpaired two-tailed *t* test | T(32) = 3.030  P=0.0048 |
|  | H | Sham male = 7  Sham female = 7  PVP male = 7  PVP female = 7 |  | two-way ANOVA with  Sidak's multiple comparisons  post hoc test | F (7, 168) = 36.05  P<0.0001 |
|  |  |  | Pre |  | Sm *vs* Pm ns  Sf *vs* Pf ns |
|  |  |  | 0.4 h |  | Sm *vs* Pm <0.0001  Sf *vs* Pf <0.0001 |
|  |  |  | d 1 |  | Sm *vs* Pm <0.0001  Sf *vs* Pf <0.0001 |
|  |  |  | d 3 |  | Sm *vs* Pm <0.0001  Sf *vs* Pf <0.0001 |
|  |  |  | 1 w |  | Sm *vs* Pm <0.0001  Sf *vs* Pf <0.0001 |
|  |  |  | 2 w |  | Sm *vs* Pm <0.0001  Sf *vs* Pf <0.0001 |
|  |  |  | 3 w |  | Sm *vs* Pm <0.0001  Sf *vs* Pf <0.0001 |
|  |  |  | 4 w |  | Sm *vs* Pm, P<0.05  Sf *vs* Pf, P<0.05 |
|  | I | Sham = 6 males + 6 females  PVP = 6 males + 6 females |  | two-way ANOVA with  Sidak's multiple comparisons  post hoc test | F (3, 44) = 0.1610  P=0.9220 |
|  |  |  | Pre |  | S-ipsi *vs* P-ipsi. =0.9999 |
|  |  |  | Day 1 |  | S-ipsi *vs* P-ipsi. >0.9999 |
|  |  |  | Day 3 |  | S-ipsi *vs* P-ipsi. =0.9999 |
|  |  |  | Day 7 |  | S-ipsi *vs* P-ipsi. >0.9999 |
|  |  |  | Day 14 |  | S-ipsi *vs* P-ipsi. =0.9939 |
|  |  |  | Day 28 |  | S-ipsi *vs* P-ipsi. =0.9073 |
|  |  |  | Day 56 |  | S-ipsi *vs* P-ipsi. >0.9999 |
|  |  |  | Day 91 |  | S-ipsi *vs* P-ipsi. >0.9999 |
| Figure 2 | A | Sham = 12~19 sections from at least 5 males  PVP = 12~19 sections from at least 5 males |  | two-way ANOVA with  Sidak's multiple comparisons  post hoc test | Artery: F (1, 172) = 131.3,  P<0.0001  Vein: F (1, 182) = 76.07  P<0.0001 |
|  |  |  | Day 1 |  | Artery: =0.0007  Vein: =0.0404 |
|  |  |  | Day 3 |  | Artery: =0.0011  Vein: =0.0174 |
|  |  |  | Day 7 |  | Artery: =0.0025  Vein: =0.0213 |
|  |  |  | Day 14 |  | Artery: <0.0001  Vein: =0.0147 |
|  |  |  | Day 28 |  | Artery: =0.0066  Vein: =0.0011 |
|  |  |  | Day 56 |  | Artery: =0.0015  Vein: =0.0373 |
|  |  |  | Day 91 |  | Artery: =0.0037  Vein: =0.0357 |
|  | B | Sham = 12~19 sections from at least 5 males  PVP = 12~19 sections from at least 5 males |  | two-way ANOVA with  Sidak's multiple comparisons  post hoc test | CD68: F (1, 159) = 34.97  P<0.0001  CD68/ET-1: F (1, 194) = 0.07425, P=0.7855 |
|  |  |  | Day 1 |  | CD68: =0.0115  CD68/ET-1: >0.9999 |
|  |  |  | Day 3 |  | CD68: =0.0451  CD68/ET-1: =0.9947 |
|  |  |  | Day 7 |  | CD68: =0.0264  CD68/ET-1: >0.9999 |
|  |  |  | Day 14 |  | CD68: =0.0489  CD68/ET-1: >0.9999 |
|  |  |  | Day 28 |  | CD68: =0.0068  CD68/ET-1: =0.9521 |
|  |  |  | Day 56 |  | CD68: =0.8789  CD68/ET-1: =0.9973 |
|  |  |  | Day 91 |  | CD68: =0.0115  CD68/ET-1: >0.9999 |
|  | C | Sham: males  PVP: males | | | |
| Figure 3 | A, D | Males | | | |
| Figure 4 | A | males | | | |
|  | BC | Sham = 10~19 sections from at least 5 males  PVP = 10~21 sections from at least 5 males |  | two-way ANOVA with  Sidak's multiple comparisons  post hoc test | F (1, 38) = 116.6  P<0.0001 |
|  |  |  | 1 w |  | <0.0001 |
|  |  |  | 2 w |  | <0.0001 |
|  |  |  | 4 w |  | <0.0001 |
|  |  |  | 8 w |  | =0.0075 |
|  |  |  | 13 w |  | =0.0006 |
|  | D | Vehicle = 7 males  BQ-123 = 8 males |  | two-way ANOVA with  Sidak's multiple comparisons  post hoc test | F (1, 13) = 6.795  P = 0.0217 |
|  |  |  | Baseline |  | >0.9999 |
|  |  |  | Pre |  | >0.9999 |
|  |  |  | 1 h |  | = 0.1118 |
|  |  |  | 3 h |  | <0.0001 |
|  |  |  | 24 h |  | >0.9999 |
|  | E | Vehicle = 8 males  BQ-788 = 7 males |  | two-way ANOVA with  Sidak's multiple comparisons  post hoc test | F (1, 13) = 0.4391  P=0.5191 |
|  |  |  | Baseline |  | >0.9999 |
|  |  |  | Pre |  | =0.9933 |
|  |  |  | 1 h |  | =0.9987 |
|  |  |  | 3 h |  | =0.9012 |
|  |  |  | 24 h |  | =0.9999 |
|  | F | Vehicle = 7 males  BQ-123 = 8 males |  | two-way ANOVA with  Sidak's multiple comparisons  post hoc test | F (1, 13) = 11.42  P=0.0049 |
|  |  |  | Baseline |  | >0.9999 |
|  |  |  | Pre |  | >0.9999 |
|  |  |  | 1 h |  | =0.2643 |
|  |  |  | 3 h |  | <0.0001 |
|  |  |  | 24 h |  | =0.1045 |
|  | G | Vehicle = 7 males  BQ-788 = 7 males |  | two-way ANOVA with  Sidak's multiple comparisons  post hoc test | F (1, 12) = 0.5637  P=0.4672 |
|  |  |  | Baseline |  | >0.9999 |
|  |  |  | Pre |  | >0.9999 |
|  |  |  | 3 h |  | =0.9998 |
|  |  |  | 3 h |  | =0.8499 |
|  |  |  | 3 h |  | =0.9352 |
| Figure 5 | BCD | Sham = 537 neurons from 3 males  PVP = 581 neurons from 3 males | | Unpaired two-tailed *t* test | T (1116) = 17.40  P<0.0001 |
|  | E | Sham = 3 males  PVP = 3 males | | Unpaired two-tailed *t* test | T (4) = 4.263  P=0.0130 |
|  | G | Sham = 26 neurons from 4 males  PVP = 26 neurons from 6 males | | Chi-square with Yates' correction | χ^2^ (1) = 3.861  P=0.0494 |
|  | H |  |  | Unpaired two-tailed *t* test | T (50) = 2.150  P=0.0364 |
|  | I |  |  | two-way ANOVA with  Sidak's multiple comparisons  post hoc test | F (1, 50) = 2.391  P=0.1283  PVP: vehicle *vs.* ET-1 P=0.0280 |
|  | J |  |  |  | F (1, 50) = 8.830  P=0.0045  ET-1: sham *vs* PVP P=0.0381 |
|  | L |  |  |  | F (1.243, 62.15) = 76.91  P<0.0001 |
| Figure 6 | B | Tek^-^/ChR2^+/-^ = 4 males + 3 females Tek^cre^/ChR2^+/-^ = 5 males + 4 females | | Unpaired two-tailed *t* test | T (14) = 302.0  P<0.0001 |
|  | D | Tek^-^/ChR2^+/-^ = 4 males + 4 females Tek^cre^/ChR2^+/-^ = 5 males + 4 females |  | two-way ANOVA with  Sidak's multiple comparisons  post hoc test | F (1, 15) = 3116  P<0.0001  Flinching <0.0001  Licking <0.0001  Jumping <0.0001 |
|  | E | Tek^-^/ChR2^+/-^ = 4 males + 4 females  Tek^cre^/ChR2^+/-^ = 4 males + 4 females |  | two-way ANOVA with  Sidak's multiple comparisons  post hoc test | F (1, 14) = 1199  P<0.0001 |
|  |  |  | Baseline |  | =0.9887 |
|  |  |  | 3 h |  | =0.0001 |
|  |  |  | 6 h |  | =0.0001 |
|  |  |  | Day 1 |  | <0.0001 |
|  |  |  | Day 3 |  | =0.0001 |
|  |  |  | Day 7 |  | =0.0001 |
|  |  |  | 2 w |  | =0.0001 |
|  |  |  | 4 w |  | =0.0002 |
|  |  |  | 6 w |  | <0.0001 |
|  |  |  | 8 w |  | =0.0006 |
|  |  |  | 10 w |  | =0.0004 |
|  | G | Tek^-^/ hM3Dq^+/-^ = 3 males + 3 females  Tek^cre^/ hM3Dq^+/-^ = 3 males + 3 females | | Unpaired two-tailed *t* test | T (10) = 8.794  P<0.0001 |
|  | H | Tek^-^/ hM3Dq^+/-^ = 3 males + 3 females  Tek^cre^/ hM3Dq^+/-^ = 3 males + 3 females |  | two-way ANOVA with  Sidak's multiple comparisons  post hoc test | F (1, 13) = 375.6  P<0.0001 |
|  |  |  | Baseline |  | =0.9995 |
|  |  |  | Day 1 |  | <0.0001 |
|  |  |  | Day 7 |  | <0.0001 |
|  |  |  | Day 14 |  | <0.0001 |
|  |  |  | Day 21 |  | <0.0001 |
|  |  |  | Day 28 |  | <0.0001 |
|  |  |  | Day 42 |  | <0.0001 |
|  |  |  | Day 56 |  | <0.0001 |
|  |  |  | Day 70 |  | <0.0001 |
|  |  |  | Day 84 |  | <0.0001 |
|  | FigureS21D | Tek^-^/hM3Dq^+/-^  male = 6  female = 5  Tek^cre^/hM3Dq^+/-^  male = 6  female = 5 |  | two-way ANOVA with  Sidak's multiple comparisons  post hoc test | F (3, 18) = 3.617  P=0.0334 |
|  |  |  | Pre |  | Tek^-^ m *vs* Tek^cre^ m =0.9439  Tek^-^ f *vs* Tek^cre^ f =0.6766 |
|  |  |  | 0.4 h |  | Tek^-^ m *vs* Tek^cre^ m =0.0060  Tek^-^ f *vs* Tek^cre^ f =0.0468 |
|  |  |  | 24 h |  | Tek^-^ m *vs* Tek^cre^ m =0.7815  Tek^-^ f *vs* Tek^cre^ f =0.6109 |
|  | L | Vehicle = 3 males + 3 females  BQ-123 = 3 males + 3 females | | Unpaired two-tailed *t* test | T (11) = 2.708  P=0.0204 |
|  | M | Vehicle = 5 males + 2 females  BQ-123 = 4 males + 3 females |  | two-way ANOVA with  Sidak's multiple comparisons  post hoc test | F (1, 12) = 9.568  P=0.0093 |
|  |  |  | Baseline |  | =0.9911 |
|  |  |  | Pre |  | >0.9999 |
|  |  |  | 3 h |  | =0.1116 |
|  |  |  | 3 h |  | =0.0279 |
|  |  |  | 24 h |  | =0.9994 |
| Figure 7 | C | Placebo = 29 males + 16 females  Bosentan = 28 males+ 16 females |  | two-way ANOVA with  Sidak's multiple comparisons  post hoc test | F (1, 87) = 22.73  P<0.0001 |
|  |  |  | 3 h |  | <0.001 |
|  |  |  | 6 h |  | <0.0001 |
|  |  |  | 12 h |  | <0.0001 |
|  |  |  | 24 h |  | <0.0001 |
|  |  |  | 48 h |  | <0.0001 |
|  | D | Placebo = 29 males + 16 females  Bosentan = 28 males+ 16 females | | Unpaired two-tailed *t* test | T (87) = 2.786  P=0.0065 |
|  | E | Placebo = 29 males + 16 females  Bosentan = 28 males+ 16 females | | Unpaired two-tailed *t* test | T (87) = 3.421  P=0.0010 |
|  | F | Placebo = 29 males + 16 females  Bosentan = 28 males+ 16 females |  | two-way ANOVA with  Sidak's multiple comparisons  post hoc test | Time: F (3, 261) = 4736  P<0.0001  Treatment: F (1, 87) = 0.2930  P=0.5897 |
|  |  |  | Baseline |  | =0.9905 |
|  |  |  | 1 h |  | =0.6068 |
|  |  |  | 0.5 h |  | =0.9668 |
|  |  |  | 24 h |  | >0.9999 |
| Figure S1 |  | males |  |  |  |
| Figure S2 |  | males |  |  |  |
| Figure S3 | B | Sham = 5 males + 5 females  PVP = 5 males + 5 females | | Unpaired two-tailed *t* test | T (18) = 1.108  P=0.2825 |
|  | C | Sham = 5 males  PVP = 5 males |  | two-way ANOVA with  Sidak's multiple comparisons  post hoc test | F (1, 8) = 0.7546  P=0.4103 |
|  |  |  | 48 C^O^ |  | =0.6396 |
|  |  |  | 52 C^O^ |  | =0.7720 |
|  | D | Sham = 8 males + 8 females  PVP = 8 males + 7 females | | Unpaired two-tailed *t* test | T (29) = 0.7212  P=0.4766 |
|  | E | Sham = 8 males  PVP = 8 males |  | two-way ANOVA with  Sidak's multiple comparisons  post hoc test | F (1, 14) = 0.01649  P=0.8997 |
|  |  |  | Baseline |  | =0.9992 |
|  |  |  | 4 w |  | =0.9637 |
|  |  |  | 8 w |  | =0.4563 |
|  |  |  | 12 w |  | =0.9997 |
|  |  |  | 16 w |  | >0.9999 |
|  | F | Sham = 7 males + 9 females  PVP = 7 males + 8 females | | Unpaired two-tailed *t* test | T (29) = 0.3635  P=0.7188 |
|  | H | Sham: males = 7, females = 7  PVP: males = 9 , females = 9 |  | one-way ANOVA with Sidak’s multiple comparisons  post hoc test | F (3, 28) = 0.2329  P=0.8727  Sham *vs* PVP  Males: =0.9132  Females: 0.9577 |
|  | I | Sham = 6 males  PVP = 18 males | | Unpaired two-tailed *t* test | T (22) = 0.0456  P=0.9640 |
|  | J | Sham = 7 males  PVP = 15 males | | Unpaired two-tailed *t* test | T (20) = 2.385  P=0.0271 |
|  | K | Sham = 7 males + 9 females  PVP = 7 males + 10 females | | Unpaired two-tailed *t* test | T (31) = 4.373  P<0.0001 |
| Figure S4 | A | 8 males  Sham = 7 males  CCI = 8  males | Baseline  4 h  Day 1  Day 3  Day 7 | two-way ANOVA with  Sidak's multiple comparisons  post hoc test | F (1, 14) = 130.5  P<0.0001  P=0.8838  P<0.0001  P<0.0001  P=0.0002  P=0.5726 |
|  | B |  | Baseline  Day 1  Day 3  Day 7  Day 14 |  | F (1, 14) = 90.75  P<0.0001  P=0.9965  P=0.0018  P<0.0005  P=0.0022  P=0.0043 |
|  | C |  | Baseline  Day 2  Day 4  Day 8  Day 15 |  | F (1, 14) = 97.05  P<0.0001  P=0.9999  P=0.0002  P=0.0003  P=0.0101  P=0.0103 |
|  | D |  | Baseline  Day 1  Day 3  Day 7  Day 14  Day 21  Day 28 |  | F (1, 13) = 29.69  P=0.0001  P>0.9999  P=0.9247  P=0.0061  P=0.0170  P<0.0001  P=0.2210  P=0.8490 |
|  | E |  | Day 4  Day 8  Day 15  Day 22  Day 29 |  | F (1, 13) = 199.2  P<0.0001  P<0.0001  P<0.0001  P<0.0001  P=0.0003  P=0.1058 |
|  | F |  | Baseline  Day 1  Day 3  Day 7  Day 14  Day 21  Day 28 |  | F (1, 13) = 71.92  P<0.0001  P>0.9999  P=0.6366  P=0.5280  P=0.0146  P=0.0004  P=0.0010  P=0.0018 |
| Figure S5 | B | Male sham = 8  Male PVP = 9  Female sham = 8  Female PVP = 9 |  | one-way ANOVA with Tukey's multiple comparisons test | F (3, 30) = 2.210  P=0.1075  Sham *vs* PVP:  Males: =0.5710  Females: =0.9271  PVP males *vs* PVP females = 0.0834 |
|  | C |  |  |  | F (3, 30) = 2.501  P=0.0784  Sham *vs* PVP:  Males: =0.5517  Females: =0.8976  PVP males *vs* PVP females = 0.0583 |
|  | D |  |  |  | F (3, 30) = 0.6111  P=0.6131  Sham *vs* PVP:  Males: =0.8053  Females: =0.7473  PVP males *vs* PVP females = 0.8352 |
|  | E |  |  |  | F (3, 30) = 1.471  P=0.2424  Sham *vs* PVP:  Males: =0.6736  Females: =0.9728  PVP males *vs* PVP females = 0.2082 |
|  | F |  |  |  | F (3, 30) = 0.2720  P=0.8451  Sham *vs* PVP:  Males: =0.9880  Females: =0.9972  PVP males *vs* PVP females = 0.9494 |
|  | G |  |  |  | F (3, 30) = 0.2607  P=0.8531  Sham *vs* PVP:  Males: =0.9887  Females: =0.9972  PVP males *vs* PVP females = 0.9523 |
|  | H |  |  |  | F (3, 30) = 0.4238  P=0.7373  Sham *vs.* PVP:  Males: >0.9999  Females: =0.9484  PVP males *vs.* PVP females = 0.7623 |
|  | I |  |  |  | F (3, 30) = 0.3816  P=0.7670  Sham *vs* PVP:  Males: =0.8111  Females: =0.9328  PVP males *vs* PVP females = 0.9980 |
|  | J | Sham = 8 males  PVP = 8 males |  | Unpaired two-tailed *t* test | T (14) = 0.1757  P=0.8631 |
|  | K |  |  |  | T (14) = 1.444  P=0.8872 |
|  | L |  |  |  | T (14) = 0.2423  P=0.8121 |
|  | M |  |  |  | T (14) = 1.311  P=0.2111 |
| Figure S6 |  | Sham = 5 males  PVP = 5 males | | | |
| Figure S7 |  | PVP sham = 8 males + 8 females  PVP = 9 males + 9 females  HLI sham = 8 males + 7 female  HLI = 8 males + 8 females | | | |
| Figure S8 | B | HLI sham = 8 males + 7 female  HLI = 8 males + 8 females  PVP sham = 8 males + 8 females  PVP = 9 males + 9 females |  | one-way ANOVA with Tukey's multiple comparisons test | F (3, 61) = 8.194  P=0.0001  HLI sham *vs* HLI =0.0009  PVP sham *vs* PVP =0.9692  HLI *vs* PVP =0.0014 |
|  | C |  |  |  | F (3, 61) = 8.442  P<0.0001  HLI sham *vs* HLI =0.0009  PVP sham *vs* PVP =0.9989  HLI *vs* PVP =0.0011 |
|  | D |  |  |  | F (3, 61) = 1.658  P=0.1855  HLI sham *vs* HLI =0.1511  PVP sham *vs* PVP >0.9999  HLI *vs* PVP =0.4160 |
|  | E |  |  |  | F (3, 61) = 8.655  P<0.0001  HLI sham *vs* HLI =0.0006  PVP sham *vs* PVP =0.9622  HLI *vs* PVP =0.0010 |
|  | F | HLI sham = 8 males + 7 female  HLI = 8 males + 7 females (one mouse with severe toe necrosis was euthanized.)  PVP sham = 8 males + 8 females  PVP = 9 males + 9 females |  | one-way ANOVA with Tukey's multiple comparisons test | F (3, 60) = 5.332  P=0.0025  HLI sham *vs* HLI =0.0019  PVP sham *vs* PVP =0.9783  HLI *vs* PVP =0.0194 |
|  | G |  |  |  | F (3, 60) = 5.389  P=0.0024  HLI sham *vs* HLI =0.0017  PVP sham *vs* PVP =0.9791  HLI vs PVP =0.0198 |
|  | H |  |  |  | F (3, 60) = 3.702  P=0.0164  HLI sham *vs* HLI =0.0188  PVP sham *vs* PVP =0.9852  HLI *vs* PVP =0.0900 |
|  | I |  |  |  | F (3, 60) = 6.670  P=0.0006  HLI sham *vs* HLI =0.0005  PVP sham *vs* PVP =0.8645  HLI *vs* PVP =0.0057 |
|  | K |  |  |  | F (3, 60) = 15.17  P<0.0001  HLI sham *vs* HLI <0.0001  PVP sham *vs* PVP =0.6435  HLI *vs* PVP =0.0002 |
| Figure S9 |  | Sham = 12~19 sections from at least 5 males  PVP = 12~19 sections from at least 5 males | | | |
| Figure S10 | A | Sham = 12~13 sections from at least 5 males  PVP = 12~16 sections from at least 5 males |  | two-way ANOVA with  Sidak's multiple comparisons  post hoc test | Artery: F (1, 141) = 1.008,  P=0.3243  Vein: F (1, 165) = 110.4  P<0.0001 |
|  |  |  | Day 1 |  | Artery: =0.9999  Vein: =0.0194 |
|  |  |  | Day 3 |  | Artery: >0.9999  Vein: =0.0291 |
|  |  |  | Day 7 |  | Artery: =0.9276  Vein: =0.0212 |
|  |  |  | Day 14 |  | Artery: >0.9999  Vein: =0.0002 |
|  |  |  | Day 28 |  | Artery: =0.9850  Vein: =0.0026 |
|  |  |  | Day 56 |  | Artery: =0.9509  Vein: =0.0037 |
|  |  |  | Day 91 |  | Artery: >0.9999  Vein: =0.0021 |
|  | B | Sham = 13~15 sections from at least 5 males  PVP = 13 sections from at least 5 males |  | two-way ANOVA with  Sidak's multiple comparisons  post hoc test | CD68: F (1, 170) = 37.86  P<0.0001  CD68/ET-1: F (1, 144) = 0.2156  P=0.6463 |
|  |  |  | Day 1 |  | CD68: =0.0426  CD68/ET-1: =0.9983 |
|  |  |  | Day 3 |  | CD68: =0.0398  CD68/ET-1: >0.9999 |
|  |  |  | Day 7 |  | CD68: =0.0080  CD68/ET-1: >0.9999 |
|  |  |  | Day 14 |  | CD68: =0.0280  CD68/ET-1: =0.7714 |
|  |  |  | Day 28 |  | CD68: =0.0151  CD68/ET-1: >0.9999 |
|  |  |  | Day 56 |  | CD68: >0.9999  CD68/ET-1: =0.9973 |
|  |  |  | Day 91 |  | CD68: >0.9999  CD68/ET-1: =0.9996 |
|  | C | Sham: males  PVP: males | | | |
| Figure S11 |  | Sham = 12~13 sections from at least 5 males  PVP = 12~16 sections from at least 5 males | | | |
| Figure S12 |  | males | | | |
| Figure 3 | B | contralateral = 4~5 sections from at least 3 males  ipsilateral = 4~7 sections from at least 3 males |  | two-way ANOVA with  Sidak's multiple comparisons  post hoc test | F (1, 43) = 151.2  P<0.0001 |
|  |  |  | Day 1 |  | =0.0128 |
|  |  |  | Day 3 |  | =0.1002 |
|  |  |  | Day 7 |  | =0.0041 |
|  |  |  | Day 14 |  | =0.0002 |
|  |  |  | Day 28 |  | =0.0326 |
|  |  |  | Day 56 |  | =0.0011 |
|  |  |  | Day 91 |  | =0.0159 |
|  | C | contralateral = 4~6 sections from at least 3 males  ipsilateral = 4~5 sections from at least 3 males |  | two-way ANOVA with  Sidak's multiple comparisons  post hoc test | F (1, 50) = 132.4  P<0.0001 |
|  |  |  | Day 1 |  | =0.0243 |
|  |  |  | Day 3 |  | =0.0109 |
|  |  |  | Day 7 |  | =0.0135 |
|  |  |  | Day 14 |  | =0.0009 |
|  |  |  | Day 28 |  | =0.1147 |
|  |  |  | Day 56 |  | =0.0617 |
|  |  |  | Day 91 |  | =0.0346 |
|  | E | contralateral = 4~5 sections from at least 3 males  ipsilateral = 4~5 sections from at least 3 males |  | two-way ANOVA with  Sidak's multiple comparisons  post hoc test | F (1, 38) = 2.837  P=0.1306 |
|  |  |  | Day 1 |  | =0.7030 |
|  |  |  | Day 3 |  | >0.9999 |
|  |  |  | Day 7 |  | =0.9768 |
|  |  |  | Day 14 |  | >0.9999 |
|  |  |  | Day 28 |  | =0.9631 |
|  |  |  | Day 56 |  | >0.9999 |
|  |  |  | Day 91 |  | =0.6790 |
|  | F | contralateral = 4~5 sections from at least 3 males  ipsilateral = 4~5 sections from at least 3 males |  | two-way ANOVA with  Sidak's multiple comparisons  post hoc test | F (1, 39) = 69.71  P<0.0001 |
|  |  |  | Day 1 |  | =0.0548 |
|  |  |  | Day 3 |  | =0.0210 |
|  |  |  | Day 7 |  | =0.0021 |
|  |  |  | Day 14 |  | =0.0630 |
|  |  |  | Day 28 |  | =0.0138 |
|  |  |  | Day 56 |  | =0.0268 |
|  |  |  | Day 91 |  | =0.0455 |
| Figure S13 | A-D | males |  | | |
| Figure S14 | A | contralateral = 7~9 sections from at least 3 males  ipsilateral = 7~9 sections from at least 3 males |  | two-way ANOVA with  Sidak's multiple comparisons  post hoc test | F (1, 71) = 37.60  P<0.0001 |
|  |  |  | Day 1 |  | =0.0014 |
|  |  |  | Day 3 |  | =0.0037 |
|  |  |  | Day 7 |  | =0.5871 |
|  |  |  | Day 14 |  | =0.7739 |
|  |  |  | Day 28 |  | =0.9915 |
|  | B | contralateral = 6~8 sections from at least 3 males  ipsilateral = 5~9 sections from at least 3 males |  | two-way ANOVA with  Sidak's multiple comparisons  post hoc test | F (1, 45) = 32.82  P<0.0001 |
|  |  |  | Day 1 |  | =0.0111 |
|  |  |  | Day 3 |  | =0.0501 |
|  |  |  | Day 7 |  | =0.1220 |
|  |  |  | Day 14 |  | =0.0013 |
|  |  |  | Day 28 |  | =0.7718 |
|  | C | contralateral = 7~9 sections from at least 3 males  ipsilateral = 7~9 sections from at least 3 males |  | two-way ANOVA with  Sidak's multiple comparisons  post hoc test | F (1, 55) = 61.35  P<0.0001 |
|  |  |  | Day 1 |  | =0.0005 |
|  |  |  | Day 3 |  | =0.0002 |
|  |  |  | Day 7 |  | =0.0034 |
|  |  |  | Day 14 |  | =0.0311 |
|  |  |  | Day 28 |  | =0.9017 |
|  | D | contralateral = 6~8 sections from at least 3 males  ipsilateral = 5~9 sections from at least 3 males |  | two-way ANOVA with  Sidak's multiple comparisons  post hoc test | F (1, 60) = 37.93  P<0.0001 |
|  |  |  | Day 1 |  | =0.0355 |
|  |  |  | Day 3 |  | =0.0318 |
|  |  |  | Day 7 |  | =0.2542 |
|  |  |  | Day 14 |  | =0.0162 |
|  |  |  | Day 28 |  | =0.8954 |
|  | E | PVP sham = 3 males + 3 females  PVP = 3 males + 3 females |  | two-way ANOVA with  Sidak's multiple comparisons  post hoc test | F (1, 10) = 28.40  P=0.0003 |
|  |  |  | Day 1 |  | =0.0022 |
|  |  |  | Day 3 |  | =0.0556 |
|  |  |  | Day 7 |  | =0.0290 |
|  |  |  | Day 14 |  | =0.0041 |
|  |  |  | Day 28 |  | =0.0067 |
|  |  |  | Day 56 |  | =0.0002 |
|  |  |  | Day 91 |  | =0.0003 |
|  | F | contralateral = 3 males + 2 females  ipsilateral = 3 males + 2 females |  | two-way ANOVA with  Sidak's multiple comparisons  post hoc test | F (1, 8) = 10.35  P=0.0123 |
|  |  |  | Day 1 |  | =0.2903 |
|  |  |  | Day 3 |  | =0.1926 |
|  |  |  | Day 7 |  | =0.6389 |
|  |  |  | Day 14 |  | =0.7175 |
|  |  |  | Day 28 |  | =0.6753 |
|  |  |  | Day 56 |  | =0.0506 |
|  |  |  | Day 91 |  | =0.5962 |
| Figure S15 | A | Sham = 13~15 sections from at least 5 males  PVP = 13~15 sections from at least 5 males |  | two-way ANOVA with  Sidak's multiple comparisons  post hoc test | F (1, 131) = 0.9510  P=0.3313 |
|  |  |  | 1 w |  | =0.3617 |
|  |  |  | 2 w |  | =0.7510 |
|  |  |  | 4 w |  | >0.9999 |
|  |  |  | 8 w |  | =0.9222 |
|  |  |  | 13 w |  | =0.3077 |
|  | B | contralateral = 6 sections from at least 3 males  ipsilateral = 6 sections from at least 3 males |  | two-way ANOVA with  Sidak's multiple comparisons  post hoc test | F (1, 60) = 102.3  P<0.0001 |
|  |  |  | Day 1 |  | =0.6654 |
|  |  |  | Day 3 |  | =0.0170 |
|  |  |  | Day 7 |  | =0.0215 |
|  |  |  | Day 14 |  | =0.0288 |
|  |  |  | Day 28 |  | =0.0230 |
|  |  |  | Day 56 |  | =0.0008 |
|  |  |  | Day 91 |  | =0.0095 |
|  | C | Sham = 8 males  PVP = 7 males |  | two-way ANOVA with  Sidak's multiple comparisons  post hoc test | F (1, 13) = 8.358  P=0.0126 |
|  |  |  | Baseline |  | >0.9999 |
|  |  |  | Pre |  | =0.9991 |
|  |  |  | 3 h |  | =0.4061 |
|  |  |  | 3 h |  | <0.0001 |
|  |  |  | 24 h |  | =0.9905 |
|  | D | Sham = 7 males  PVP = 7 males |  | two-way ANOVA with  Sidak's multiple comparisons  post hoc test | F (1, 12) = 19.53  P=0.0008 |
|  |  |  | Baseline |  | =0.9802 |
|  |  |  | Pre |  | >0.9999 |
|  |  |  | 3 h |  | =0.2405 |
|  |  |  | 3 h |  | =0.0003 |
|  |  |  | 24 h |  | =0.6426 |
|  | E | Sham = 7 males  PVP = 7 males |  | two-way ANOVA with  Sidak's multiple comparisons  post hoc test | F (1, 12) = 0.4777  P=0.5026 |
|  |  |  | Baseline |  | =0.9911 |
|  |  |  | Pre |  | =0.9911 |
|  |  |  | 3 h |  | >0.9999 |
|  |  |  | 3 h |  | =0.8514 |
|  |  |  | 3 h |  | =0.8514 |
|  | F | Sham = 6 males  PVP = 6 males |  | two-way ANOVA with  Sidak's multiple comparisons  post hoc test | F (1, 10) = 0.9568  P=0.3511 |
|  |  |  | Baseline |  | =0.7807 |
|  |  |  | Pre |  | =0.9897 |
|  |  |  | 3 h |  | =0.7807 |
|  |  |  | 3 h |  | =0.9897 |
|  |  |  | 3 h |  | =0.8192 |
| Figure S16 | B | Na_v_1.8^cre^/ETAR^f/f^ = 10 section from at least 3 males  Na_v_1.8^-^/ETAR^f/f^ = 12 section from at least 3 males |  | Unpaired two-tailed *t* test | T (20) = 16.57  P<0.0001 |
|  | C | WT sham = 4 males + 4 females  WT PVP = 4 males + 4 females  CKO PVP = 4 males + 4 females |  | two-way ANOVA with  Sidak's multiple comparisons  post hoc test | F (2, 21) = 57.55  P<0.0001 |
|  |  |  | Baseline |  | WT: sham *vs* PVP =0.9997  WT PVP *vs* CKO PVP =0.9979 |
|  |  |  | 2 w |  | WT: sham *vs* PVP =0.0059  WT PVP *vs* CKO PVP =0.0001 |
|  |  |  | 4 w |  | WT: sham *vs* PVP =0.0017  WT PVP *vs* CKO PVP =0.3024 |
|  |  |  | 6 w |  | WT: sham *vs* PVP =0.0006  WT PVP *vs* CKO PVP =0.2144 |
|  |  |  | 8 w |  | WT: sham *vs* PVP <0.0001  WT PVP *vs* CKO PVP =0.0022 |
|  |  |  | 10 w |  | WT: sham *vs* PVP <0.0001  WT PVP *vs* CKO PVP =0.0002 |
|  |  |  | 12 w |  | WT: sham *vs* PVP <0.0001  WT PVP *vs* CKO PVP =0.1256 |
|  |  |  | 14 w |  | WT: sham *vs* PVP <0.0001  WT PVP *vs* CKO PVP =0.0049 |
|  | D | WT sham = 4 males + 4 females  WT PVP = 4 males + 4 females  CKO PVP = 4 males + 4 females |  | two-way ANOVA with  Sidak's multiple comparisons  post hoc test | F (2, 21) = 93.07  P<0.0001 |
|  |  |  | 4 w |  | WT: sham *vs* PVP <0.0001  WT PVP *vs* CKO PVP <0.0001 |
|  |  |  | 8 w |  | WT: sham *vs* PVP <0.0001  WT PVP *vs* CKO PVP <0.0001 |
|  |  |  | 12 w |  | WT: sham *vs* PVP <0.0001  WT PVP *vs* CKO PVP <0.0001 |
|  |  |  | 14 w |  | WT: sham *vs* PVP <0.0001  WT PVP *vs* CKO PVP <0.0001 |
|  | E | males | | | |
|  | F | ET-1^f/f^ = 5 males + 4 females  Advillin^cre^/ET-1^f/f^ = 4 males + 1 female  SNS^cre^/ET-1^f/f^ = 2 males + 2 females |  | two-way ANOVA with  Sidak's multiple comparisons  post hoc test | F (2, 15) = 0.7025  P=0.5109 |
|  |  |  | Baseline |  | ET-1 *vs* SNS/ET-1 =0.8508  ET-1 *vs* Adv/ET-1 =0.8421 |
|  |  |  | 1 w |  | ET-1 *vs* SNS/ET-1 =0.2732  ET-1 *vs* Adv/ET-1 =0.5304 |
|  |  |  | 2 w |  | ET-1 *vs* SNS/ET-1 =0.4894  ET-1 *vs* Adv/ET-1 =0.9352 |
|  |  |  | 4 w |  | ET-1 *vs* SNS/ET-1 =0.9964  ET-1 *vs* Adv/ET-1 =0.7154 |
|  |  |  | 6 w |  | ET-1 *vs* SNS/ET-1 =0.8003  ET-1 *vs* Adv/ET-1 =0.9558 |
|  |  |  | 8 w |  | ET-1 *vs* SNS/ET-1 =0.8971  ET-1 *vs* Adv/ET-1 =0.5249 |
|  |  |  | 12 w |  | ET-1 *vs* SNS/ET-1 =0.8033  ET-1 *vs* Adv/ET-1 =0.9927 |
|  |  |  | 14 w |  | ET-1 *vs* SNS/ET-1 =0.3782  ET-1 *vs* Adv/ET-1 =0.9979 |
| Figure S17 | A | ET-1^f/f^ = 3 males + 6 females  Tek^cre^/ET-1^f/f^ = 3 males + 6 females |  | two-way ANOVA with  Sidak's multiple comparisons  post hoc test | F (1, 16) = 0.1520  P=0.7018 |
|  |  |  | Baseline |  | >0.9999 |
|  |  |  | Day 1 |  | >0.9999 |
|  |  |  | Day 3 |  | >0.9999 |
|  |  |  | Day 7 |  | >0.9999 |
|  |  |  | Day 14 |  | >0.9999 |
|  |  |  | Day 28 |  | =0.9003 |
|  |  |  | Day 42 |  | =0.9547 |
|  |  |  | Day 56 |  | >0.9999 |
|  |  |  | Day 98 |  | =0.8411 |
|  | B | males |  |  |  |
|  | C | ET-1^f/f^ = 6 males  Cdh5^cre^/ET-1^f/f^ = 6 males |  | two-way ANOVA with  Sidak's multiple comparisons  post hoc test | F (1, 10) = 567.1  P<0.0001 |
|  |  |  | Baseline |  | >0.9999 |
|  |  |  | Day 1 |  | =0.0016 |
|  |  |  | Day 3 |  | =0.0012 |
|  |  |  | Day 7 |  | <0.0001 |
|  |  |  | Day 14 |  | =0.0002 |
|  |  |  | Day 28 |  | =0.0069 |
|  |  |  | Day 42 |  | =0.0015 |
|  |  |  | Day 56 |  | =0.0005 |
|  |  |  | Day 91 |  | =0.0001 |
| Figure S18 | A | Sham = 11~17 sections from at least 5 males  PVP = 14~25 sections from at least 5 males |  | two-way ANOVA with  Sidak's multiple comparisons  post hoc test | F (1, 159) = 0.3438  P=0.5609 |
|  |  |  | 1 w |  | >0.9999 |
|  |  |  | 2 w |  | =0.6923 |
|  |  |  | 4 w |  | =0.9080 |
|  |  |  | 8 w |  | =0.5886 |
|  |  |  | 18 w |  | =0.7449 |
|  | B | Sham = 10~16 sections from at least 5 males  PVP = 13~15 sections from at least 5 males |  | two-way ANOVA with  Sidak's multiple comparisons  post hoc test | F (1, 50) = 0.2096  P=0.6491 |
|  |  |  | 1 w |  | =0.5170 |
|  |  |  | 4 w |  | =0.2658 |
|  | C | Sham = 15~21 sections from at least 5 males  PVP = 15~22 sections from at least 5 males |  | two-way ANOVA with  Sidak's multiple comparisons  post hoc test | F (1, 159) = 0.6545  P=0.4197 |
|  |  |  | 1 w |  | >0.9999 |
|  |  |  | 2 w |  | =0.9790 |
|  |  |  | 4 w |  | =0.9664 |
|  |  |  | 8 w |  | =0.7985 |
|  |  |  | 18 w |  | =0.8631 |
|  | D | Sham = 12 sections from at least 5 males  PVP = 12 sections from at least 5 males | | Unpaired two-tailed *t* test | T (22) = 1.313  P=0.2027 |
| Figure S19 | A | Sham = 11~17 sections from at least 5 males  PVP = 14~25 sections from at least 5 males |  | two-way ANOVA with  Sidak's multiple comparisons  post hoc test | F (1, 119) = 1.616  P=0.9939 |
|  |  |  | 1 w |  | =0.9993 |
|  |  |  | 2 w |  | =0.8385 |
|  |  |  | 4 w |  | =0.9952 |
|  |  |  | 8 w |  | =0.9542 |
|  |  |  | 18 w |  | =0.9895 |
|  | B | Sham = 15~21 sections from at least 5 males  PVP = 15~22 sections from at least 5 males |  | two-way ANOVA with  Sidak's multiple comparisons  post hoc test | F (1, 116) = 2.352  P=0.6303 |
|  |  |  | 1 w |  | =0.4405 |
|  |  |  | 2 w |  | =0.8454 |
|  |  |  | 4 w |  | =0.9453 |
|  |  |  | 8 w |  | =0.9996 |
|  |  |  | 18 w |  | =0.4162 |
| Figure S20 | A | Tek^-^/ChR2^+/-^ = 4 males + 4 females  Tek^cre^/ChR2^+/-^ = 5 males + 4 females |  | two-way ANOVA with  Sidak's multiple comparisons  post hoc test | F (1, 15) = 12138  P<0.0001 |
|  |  |  | 1 Hz |  | <0.0001 |
|  |  |  | 3 Hz |  | <0.0001 |
|  |  |  | 10 Hz |  | <0.0001 |
|  | B | Tek^-^/ChR2^+/-^ = 4 males + 4 females  Tek^cre^/ChR2^+/-^ = 4 males + 3 females | | Unpaired two-tailed *t* test |  |
|  | C | males | | | |
|  | D | Tek^-^/ChR2^+/-^=  5 males +  5 females  Tek^cre^/ChR2^+/-^= 4 males +  5 females |  | two-way ANOVA with  Sidak's multiple comparisons  post hoc test | F (3, 15) = 0.1751  P=0.9116 |
|  |  |  | Pre |  | M: Tek^-^ *vs* Tek^cre^ =0.9857  F: Tek^-^ *vs* Tek^cre^ =0.7827 |
|  |  |  | 0.4 h |  | M: Tek^-^ *vs* Tek^cre^ =0.6290  F: Tek^-^ *vs* Tek^cre^ =0.9969 |
|  |  |  | 24 h |  | M: Tek^-^ *vs* Tek^cre^ =0.7843  F: Tek^-^ *vs* Tek^cre^ =0.9904 |
|  | E |  |  | two-way ANOVA with  Sidak's multiple comparisons  post hoc test | F (3, 15) = 5.465  P=0.0097 |
|  |  |  | 1 h |  | M: Tek^-^ *vs* Tek^cre^ =0.2591  F: Tek^-^ *vs* Tek^cre^ =0.0196 |
|  |  |  | 6 h |  | M: Tek^-^ *vs* Tek^cre^ =0.3099  F: Tek^-^ *vs* Tek^cre^ =0.0932 |
|  |  |  | 24 h |  | M: Tek^-^ *vs* Tek^cre^ =0.1664  F: Tek^-^ *vs* Tek^cre^ =0.4162 |
| Figure S21 | B | Tek^-^/hM3Dq^+/-^:  5 males  5 females  Tek^cre^/ hM3Dq^+/-^:  5 males  4 females |  | two-way ANOVA with  Sidak's multiple comparisons  post hoc test | F (3, 15) = 2.888  P=0.0703 |
|  |  |  | Pre |  | M: Tek^-^ *vs* Tek^cre^ =0.9991  F: Tek^-^ *vs* Tek^cre^ =0.6849 |
|  |  |  | 0.4 h |  | M: Tek^-^ *vs* Tek^cre^ =0.0711  F: Tek^-^ *vs* Tek^cre^ =0.1373 |
|  |  |  | 24 h |  | M: Tek^-^ *vs* Tek^cre^ =0.3433  F: Tek^-^ *vs* Tek^cre^ =0.2618 |
|  | C | males | | two-way ANOVA with  Sidak's multiple comparisons  post hoc test |  |
|  | D | Tek^-^/hM3Dq^+/-^:  5 males  5 females  Tek^cre^/ hM3Dq^+/-^:  5 males  4 females |  |  | F (3, 15) = 17.49  P<0.0001 |
|  |  |  | Pre |  |  |
|  |  |  | 1 h |  | M: Tek^-^ *vs* Tek^cre^ =0.0131  F: Tek^-^ *vs* Tek^cre^ =0.1198  Tek^cre^: M *vs* F =0.1559 |
|  |  |  | 6 h |  | M: Tek^-^ *vs* Tek^cre^ =0.0680  F: Tek^-^ *vs* Tek^cre^ =0.7330 |
|  |  |  | 24 h |  | M: Tek^-^ *vs* Tek^cre^ =0.2328  F: Tek^-^ *vs* Tek^cre^ =0.2485 |
|  | E | Tek^-^/hM3Dq^+/-^:  5 males  5 females  Tek^cre^/ hM3Dq^+/-^:  5 males  4 females |  | two-way ANOVA with  Sidak's multiple comparisons  post hoc test | F (3, 18) = 28.06  P<0.0001 |
|  |  |  | Pre |  |  |
|  |  |  | 1 h |  | M: Tek^-^ *vs* Tek^cre^ <0.0001  F: Tek^-^ *vs* Tek^cre^ =0.0519  Tek^cre^: M *vs* F =0.9920 |
|  |  |  | 6 h |  | M: Tek^-^ *vs* Tek^cre^ =0.1664  F: Tek^-^ *vs* Tek^cre^ =0.3615 |
|  |  |  | 24 h |  | M: Tek^-^ *vs* Tek^cre^ =0.0826  F: Tek^-^ *vs* Tek^cre^ =0.2326 |
| Figure S22 | B | Tek^cre^-GFAP = 4 males + 4 females  Tek^cre^-hM3Dq = 4 males + 4 females | | Unpaired two-tailed *t* test | T (14) = 4.620  P=0.0004 |
|  | Figure S23B | Vehicle = 6 males  BQ-123 = 6 males |  | two-way ANOVA with  Sidak's multiple comparisons  post hoc test | F (1, 10) = 56.44  P<0.0001 |
|  |  |  | pre |  | =0.9979 |
|  |  |  | 1 h |  | =0.1487 |
|  |  |  | 3 h |  | <0.0001 |
|  |  |  | 3 h |  | <0.0001 |
|  |  |  | 3 h |  | =0.0004 |
|  |  |  | 24 h |  | =0.1668 |
|  | Figure S23C |  |  | Unpaired two-tailed *t* test | T (10) = 4.330  P=0.0015 |
| Figure S23 | Figure 6I | Tek^cre^/ChR2^+/-^  Yellow light = 4 males  Blue light = 3 males |  | two-way ANOVA with  Sidak's multiple comparisons  post hoc test | F (1, 5) = 44.82  P=0.0011 |
|  |  |  | Day 1 |  | =0.0245 |
|  |  |  | Day 7 |  | =0.0103 |
|  |  |  | Day 28 |  | =0.0399 |
|  |  |  | Day 56 |  | =0.6251 |
|  |  |  | Day 91 |  | =0.1920 |
|  | Figure S22D | Tek^cre^/ hM3Dq^+/-^:  Vehicle = 3~4 males  CNO = 3 males |  | two-way ANOVA with  Sidak's multiple comparisons  post hoc test | F (1, 29) = 16.77  P=0.0003 |
|  |  |  | Day 1 |  | =0.1012 |
|  |  |  | Day 7 |  | =0.1220 |
|  |  |  | Day 14 |  | =0.9511 |
|  |  |  | Day 28 |  | =0.8718 |
|  |  |  | Day 56 |  | =0.9126 |
|  |  |  | Day 91 |  | =0.8224 |
|  | Figure 6J | Tek^cre^/ChR2^+/-^  contralateral = 3 males  ipsilateral = 3 males |  | two-way ANOVA with  Sidak's multiple comparisons  post hoc test | F (1, 4) = 14.18  P=0.0197 |
|  |  |  | Day 1 |  | =0.0167 |
|  |  |  | Day 7 |  | =0.6525 |
|  |  |  | Day 28 |  | =0.0498 |
|  |  |  | Day 56 |  | =0.8684 |
|  |  |  | Day 91 |  | =0.4762 |
|  | Figure S22E | Tek^cre^/ hM3Dq^+/-^:  Vehicle = 3~4 males  CNO = 3 males |  | two-way ANOVA with  Sidak's multiple comparisons  post hoc test | F (1, 4) = 1.131  P=0.3474 |
|  |  |  | Day 1 |  | >0.9999 |
|  |  |  | Day 7 |  | =0.9998 |
|  |  |  | Day 14 |  | =0.9938 |
|  |  |  | Day 28 |  | =0.9990 |
|  |  |  | Day 56 |  | =0.9998 |
|  |  |  | Day 91 |  | =0.9976 |
|  | D | Cdh5^+^/hM3Dq^+/-^ = 6 males  Cdh5^cre^/ hM3Dq^+/-^/ET-1^f/f^ = 6 males |  | Unpaired two-tailed *t* test | T (10) = 7.944  P<0.0001 |
|  | E | Cdh5^+^/hM3Dq^+/-^ = 6 males  Cdh5^cre^/ hM3Dq^+/-^/ET-1^f/f^ = 6 males |  | two-way ANOVA with  Sidak's multiple comparisons  post hoc test | F (1, 10) = 59.02  P<0.0001 |
|  |  |  | Baseline |  | >0.9999 |
|  |  |  | Day 1 |  | =0.0335 |
|  |  |  | Day 3 |  | =0.0254 |
|  |  |  | Day 7 |  | =0.0031 |
|  |  |  | Day 14 |  | =0.0067 |
|  |  |  | Day 28 |  | =0.0165 |
|  |  |  | Day 42 |  | =0.1349 |
|  |  |  | Day 56 |  | =0.4438 |
|  |  |  | Day 91 |  | =0.1606 |
| Figure S24 | A | males | | | |
|  | B | Tek^-^/ChR2^+/-^=  10~14 sections from at least 5 males  Tek^cre^/ChR2^+/-^= 10~15 sections from at least 5 males |  | two-way ANOVA with  Sidak's multiple comparisons  post hoc test | F (1, 122) = 326.8  P<0.0001 |
|  |  |  | 1 w |  | =0.0006 |
|  |  |  | 2 w |  | <0.0001 |
|  |  |  | 4 w |  | <0.0001 |
|  |  |  | 8 w |  | <0.0001 |
|  |  |  | 13 w |  | <0.0001 |
|  | C | males | | | |
|  | D | Contralateral = 6 sections from at least 3 males  ipsilateral = 6 sections from at least 3 males |  | two-way ANOVA with  Sidak's multiple comparisons  post hoc test | F (1, 10) = 255.5  P<0.0001 |
|  |  |  | Day 1 |  | =0.0004 |
|  |  |  | Day 3 |  | =0.0012 |
|  |  |  | Day 7 |  | =0.0276 |
|  |  |  | Day 14 |  | =0.0003 |
|  |  |  | Day 28 |  | =0.0078 |
|  |  |  | Day 42 |  | <0.0001 |
|  |  |  | Day 56 |  | =0.0030 |
| Figure S25 | B | Vehicle = 3 males + 2 females  BQ-123 = 3 males + 3 females |  | two-way ANOVA with  Sidak's multiple comparisons  post hoc test | F (1, 9) = 0.2506  P=0.6287 |
|  |  |  | Baseline |  | =0.9953 |
|  |  |  | pre |  | =0.9994 |
|  |  |  | 3 h |  | =0.9994 |
|  |  |  | 3 h |  | =0.6409 |
|  |  |  | 3 h |  | =0.9994 |
|  | D | Vehicle = 5 males  BQ-123 = 5 males | | Unpaired two-tailed *t* test | T (8) = 2.345  P=0.0470 |
